# The new life of traditional water treatment flocculant polyaluminum chloride (PAC): a green and efficient micro–nano reactor catalyst in alcohol solvents†

**DOI:** 10.1039/d1ra08038e

**Published:** 2021-12-23

**Authors:** Gang Wang, Pengcheng Hao, Yanping Liang, Yuwang Liang, Wanyi Liu, Jiantong Wen, Xiang Li, Haijuan Zhan, Shuxian Bi

**Affiliations:** State Key Laboratory of High-efficiency Utilization of Coal and Green Chemical Engineering, National Demonstration Center for Experimental Chemistry Education, College of Chemistry and Chemical Engineering, Ningxia University Yinchuan 750021 P. R. China liuwy@nxu.edu.cn

## Abstract

Polyaluminum chloride (PAC) is an inorganic polymer material that has the advantages of a simple preparation process and special electronic structure. It is considered to be the most efficient and widely used flocculation material for water treatment. In this work, PAC has been used as a Lewis acid catalyst in interdisciplinary fields because of its polynuclear Al–O cation structure. Further, its catalytic mechanism in green organic synthesis has been studied in detail by using the multicomponent Biginelli reaction as the probe. The effect of solvent on the self-assembly and aggregation process of PAC materials was investigated using optical microscopy, UV-Vis spectrophotometry, particle size analysis, XPS, IR, SEM and HR-TEM. The results show that the PAC materials have different morphological characteristics in different solvents. The Al–O–Al cations were transformed in the ethanol solvent to form new multi-nuclear cation aggregates Al_b_, which could be used as inorganic micro–nano reactors with unique synergistic catalysis in catalytic reactions. This is the first time the role of PAC in the Biginelli reaction has been analyzed with a liquid *in situ* infrared instrument, which provided favorable evidence for the speculated reaction mechanism. The PAC–ethanol system is, therefore, considered to be a green, efficient (best yield >99%), economic and recyclable catalyst for catalyzing organic synthesis reactions. The development and utilization of PAC materials in organic synthesis will bring new vitality to this cheap material, which is widely used in industries.

## Abstract 2

The polyaluminum chloride–ethanol micro–nano reactor is a green, efficient, easy-to-handle and economical catalyst for catalyzing organic synthesis reactions.

## Graphical abstract

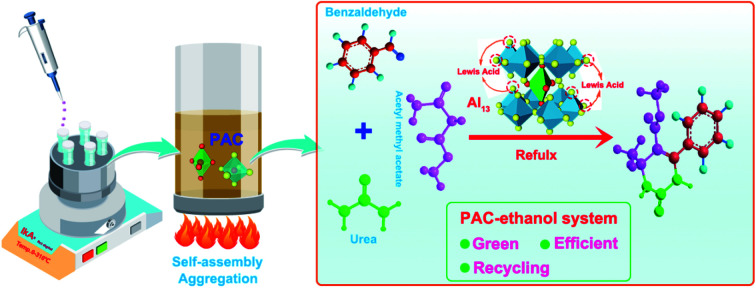

## Introduction

Polyaluminum chloride (Al_2_Cl_*n*_(OH)_6−*n*,_ PAC) is currently the most commonly used flocculant for water treatment in industrial production, with its usage reaching up to 3000–4000 tons per day.^1–5^ It has been reported that polyaluminum chloride can exist in three different forms in aqueous solutions: monomer (Al_a_), fast reaction colloid (Al_b_) and slow reaction colloid (Al_c_).^6^ Researchers believe that the highly charged polymerized ring chain of the Al_13_ Keggin structure (Al_13_O_4_(OH)_24_^7+^), which is mainly composed of octahedral aluminum sites (Al(O)_6_) and tetrahedral (Al(O)_4_) site structure,^7,8^ is the most stable and efficient cationic Lewis acid in the wastewater treatment process. In 2020,^9^ we first reported polyaluminum chloride as a highly efficient and green catalyst for the Friedel–Crafts alkylation of bis(indolyl)methane. However, the morphology of PAC materials during self-assembly and aggregation in different solvents (especially non-aqueous solvents) has not been studied in detail, and the changes before and after the use of PAC materials in ethanol have not been thoroughly revealed.^10–14^ Therefore, further development in the catalytic properties of the PAC–ethanol catalytic system in organic synthesis is of great significance to the sustainable development of chemistry and the chemical industry.

In the 21st century, green synthesis and catalysis have gradually become a topic of importance to chemists.^15,16^ For multicomponent reactions, the Biginelli reaction is the simplest way to construct dicyanopyridone/pyrimidinone (DHPMs) and its derivatives.^17–19^ The reaction product has been widely used as an anti-tumor, anti-bacterial, anti-inflammatory, and anti-viral agent, as well as in other medical fields.^20–22^ Since the discovery of the reaction more than 100 years ago, researchers have successively developed different catalytic systems and synthesis strategies;^23–28^ despite all the improvements toward better reaction conditions, many drawbacks associated with this transformation still exist. Problems, such as low yields, long reaction times, cost, unsustainable catalysts, and purification issues among others, still pose challenges to the scientific community; especially, when metal materials are used to catalyze organic synthesis reactions, trace metal residues are often detected in the compounds.^29–31^ In addition, there are many other disputes regarding the catalytic mechanism of this reaction.^32–34^ Researchers have successively proposed three different catalytic mechanisms, namely iminium, Knoevenagel and enamine pathways.^35^ For example, in 2007, when Maradur used HPVMo_11_ to catalyze the Biginelli reaction, it was speculated that the aldehyde and amine would react first.^36^ In 2017, Selvakumar used a supported heteropolyacid to catalyze the Biginelli reaction, and it was speculated that the aldehyde and the triethyl group reacted first.^37^ However, these studies lack an understanding of the kinetics of this classic multi-component tandem reaction. Hence, it is also of great significance to further explore the process mechanism of the reaction with instruments such as infrared *in situ* online instruments based on our previous work.^9,10^

Here, we have explored in detail the self-assembly and aggregation states of polyaluminum chloride in different solvents (CH_3_CN, DMSO, CH_2_Cl_2_, THF, EtOH, MeOH, isopropanol, ethylene glycol, and glycerin). Moreover, the particle size, morphology and nano-catalytic mechanism of PAC materials in ethanol as a representative non-aqueous solvent were studied using the one-pot multicomponent Biginelli reaction as a model. In addition, we have also provided kinetic information about the surface interaction of PAC materials at the molecular scale and analyzed the catalytic properties of the colloidal solution formed by PAC and ethanol. These research conclusions based on particle size analysis, optical microscopy, UV spectrophotometry, XPS, IR, SEM and HR-TEM provide new ideas for the development and utilization of cationic inorganic polymer materials (represented by PAC) in organic synthesis.

## Results and discussion

To optimize the reaction conditions, a series of control experiments were conducted to study the influence of various parameters on this reaction, and the results are summarized in Table 1. The effect of different temperatures on the reaction yield was analyzed (Table 1, entries 1–3), and it was found that the yield increased while the temperature increased. When the temperature reached the reflux temperature, a yield of 88% could be obtained (Table 1, entry 3). It is evident that the amount of catalyst used also had a great impact on the reaction yield (Table 1, entries 4–6). When 0.07 g PAC was used as the catalyst, the best yield of 99% was obtained after five hours (Table 1, entry 6). It is worth noting that the catalytic performance of anhydrous AlCl_3_ was better than that of AlCl_3_·6H_2_O (entries 7 and 8). This indicated that anhydrous AlCl_3_ had stronger Lewis acidity. However, it cannot be recycled, and most anhydrous metal chloride salts can easily explode, which is not conducive to storage and use. In addition, Al(OH)_3_ was not a suitable catalyst for this reaction (Table 1, entry 9). The possible reason is that the colloidal form of aluminum hydroxide in the solution limits the activity of Al atoms. In contrast, the inorganic polymer PAC exhibited higher catalytic performance and is more convenient for storage; hence, it is of great significance to continue to develop PAC materials.

**Table 1:** Optimization of the Biginelli reaction conditions catalyzed by PAC^a^

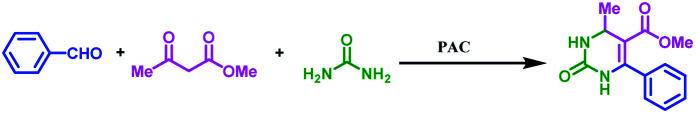				
Entry	Solvent (mL)	Catalyst (g)	Temp. (°C)	Yield^b^ (%)
1	EtOH	0.05	40	53
2	EtOH	0.05	60	78
3	EtOH	0.05	Reflux	88
4	EtOH	0.03	Reflux	78
5	EtOH	0.06	Reflux	90
6	EtOH	0.07	Reflux	99
7^c^	EtOH	0.07	Reflux	89
8^c^	EtOH	0.07	Reflux	73
9^c^	EtOH	0.07	Reflux	10

a Reaction conditions: benzaldehyde (1.0 mmol), acetyl methyl acetate (1.0 mmol), urea (1.5 mmol), solvent (3.0 mL), and PAC catalysts stirred at reflux temperature in the air for 5 h.

b Isolated yields.

c The catalysts were replaced with: AlCl_3_, AlCl_3_·6H_2_O, Al(OH)_3_, respectively.

It is well known that catalysts exhibit different behaviour in different solvents, but there are few detailed discussions in published papers. Therefore, the effect of different solvents on the synthesis of 3,4-dihydropyrimidine-2(1*H*)-one using PAC catalysts is discussed here. It was found that the catalytic effect of PAC in the aqueous solution was very poor, and the reaction yield was only 30% (Table 2, entry 1). However, it showed good catalytic performance in non-aqueous solvents (Table 2, entries 2–8), including both strongly polar solvents, such as alcohol, acetonitrile, DMSO, DMF, and ethyl acetate, and the weak polar solvents THF and CH_2_Cl_2_. The huge difference in the catalytic activity of PAC materials in different solvents may be related to the dispersion state and catalytically active species.^38^ PAC materials can appear in three different states in a solvent: monomer Al_a_ (*e.g.*: Al^3+^, Al(OH)^2+^, Al(OH)^+^, Al(OH)_3_, Al(OH)_4_^−^), rapid reaction polymer Al_b_ (*e.g.*: Al_2_(OH)^5+^, Al_2_(OH)_2_^4+^, Al_3_(OH)_4_^5+^, Al_4_(OH)_8_^4+^, Al_6_(OH)_12_^6+^, Al_13_O_4_(OH)_24_^7+^, Al_8_(OH)_20_^4+^, *etc.*), and slow reaction colloid Al_c_ (*e.g.*: Al(OH)_3_ (s)). Among these, the Al(OH)_3_ colloids had almost no catalytic effect (Table 1, entry 9). The dissolution state of PAC materials in different solvents can reasonably explain the differences in their catalytic performance (Fig.S1 and S2†). According to the photographs showing dissolution at different time periods, it can be seen that PAC dissolved the fastest in water, reaching complete dissolution after about 3 minutes (Fig. S1, a†). The Tyndall effect appeared in the aqueous solution, which proves that the Al(OH)_3_ colloid was produced after the PAC material was dissolved in the aqueous solution (Fig. S2, f†). The rapid hydrolysis of PAC in aqueous solutions has great advantages in the sedimentation process of water pollutants, but this characteristic is not conducive to its catalytic organic reaction in aqueous solutions because it is well-known that Al(OH)_3_ has a poor catalytic effect. However, PAC is basically insoluble in aprotic solvents (like CH_3_CN, DMSO, CH_2_Cl_2_, THF, DMF, ethyl acetate), and ultrasound or stirring can only play a role in dispersion. In other words, PAC materials can self-assemble and aggregate in aprotic solvents but not dissolve (Fig. S1, b and c†). At this time, the Al species present in the solution would mainly be Al_a_ monomers and a small amount of Al_b_. Therefore, a moderate yield could be obtained in the catalytic reaction (Table 2, entries 2–7). However, in protic organic solvents (ethanol, methanol and isopropanol), PAC materials would also undergo hydroxylation due to the presence of hydroxyl groups; however, it could be clearly seen that the dissolution rate of PAC in alcoholic solvents was relatively slow, and partial precipitation could be observed at the bottom of the tube even after 120 minutes (Fig. S1, d†). Therefore, we speculated that PAC was relatively stable in ethanol because it could self-assemble and aggregate in ethanol to form a new active species Al_b_, which remained unchanged in ethanol for a long time; thus, the PAC–ethanol catalytic system displayed the highest catalytic ability (Table 2, entry 8, yield = 99%). Although PAC undergoes hydroxylation in ethylene glycol and glycerol, the catalytic results were not ideal, which may be related to solvent viscosity. Since the high viscosity of ethylene glycol and glycerol is not conducive to the self-assembly of PAC materials, the catalytic performance was poor (Table 2, entries 11 and 12). Although PAC has good stability in aprotic solvents (CH_3_CN, DMSO, CH_2_Cl_2_, THF, DMF, ethyl acetate), the use of this type of solvents for the catalytic process causes environmental pollution, which is not in line with the concept of green chemistry. At the same time, considering the cost of solvents and relative stability, the PAC–ethanol catalytic system can finally be considered a green and economical choice.

**Table 2:** The effect of different solvents on the synthesis of 3,4-dihydropyrimidine-2(1*H*)-one using PAC as the catalyst^a^

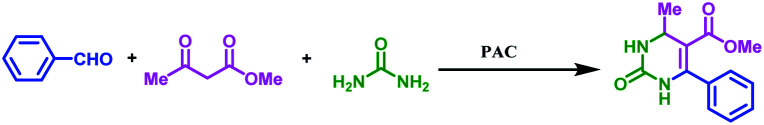				
Entry	Solvent (mL)	Catalyst (g)	Temp. (°C)	Yield^b^ (%)
1	H_2_O	0.07	Reflux	30
2	CH_3_CN	0.07	Reflux	70
3	DMSO	0.07	110	75
4	CH_2_Cl_2_	0.07	Reflux	73
5	THF	0.07	Reflux	71
6	DMF	0.07	110	69
7	Ethyl acetate	0.07	Reflux	65
8	EtOH	0.07	Reflux	99
9	CH_3_OH	0.07	Reflux	88
10	Isopropanol	0.07	Reflux	77
11	Ethylene glycol	0.07	110	43
12	Glycerol	0.07	110	Trace

a Reaction conditions: benzaldehyde (1.0 mmol), acetyl methyl acetate (1.0 mmol), urea (1.5 mmol), solvent (3.0 mL), and PAC catalysts stirred at reflux temperature or 110 °C in the air for 5 h.

b Isolated yields.

After determining the optimal reaction conditions, we explored the universality of green and cheap PAC with different reaction substrates and obtained a series of pyrimidinone or thione derivatives; the results are shown in Table 3. It can be seen from Table 3 that all the substrates could complete the reaction with a good yield. When the electron-donating group was introduced in benzaldehyde, the yield of the product reduced (4a–4c). This proves that the introduction of the electron-donating group was not conducive to the progress of the Biginelli reaction. When halogen groups were introduced in benzaldehyde, the yields of the reactions were in the following order: –F < –Cl < –Br < –I (4e–4h). This may be because the electron-withdrawing groups lead to a decrease in the electron density of the entire benzene ring, which increases the positive charge on the carbonyl group of benzaldehyde, thus facilitating the production of pyrimidinone products. However, when there are more sterically hindering groups on the benzene ring of benzaldehyde, the yield of the reaction would decrease. For example, the yields of 2,4-dichloro (4i) and 4-phenyl (4j) were 55% and 75%, respectively. In addition, when ethyl acetoacetate (4l, 4m, 4n) and benzyl acetoacetate (4o) were used as the reaction substrates, the corresponding reaction products were obtained with yields higher than 75%. The biological and medicinal value of sulfur-containing compounds is far greater than general compounds. Therefore, we tried the Biginelli reaction involving thiourea. The results showed that the PAC materials could catalyze this type of reaction and obtain a moderate yield (4p, 60%).

**Table 3:** The scope of the Biginelli reaction substrates catalyzed by PAC^a^^b^

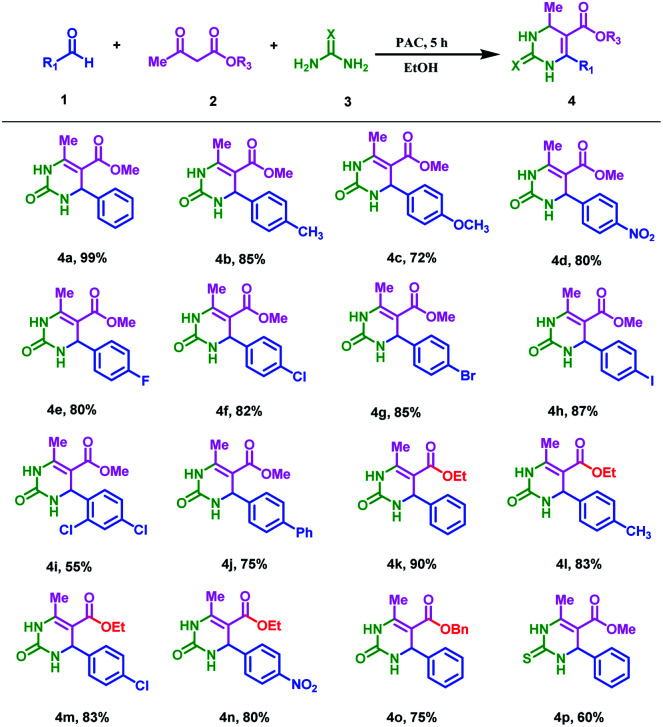

a Reaction conditions: benzaldehyde (1.2 mmol), acetyl methyl acetate (1.0 mmol), urea (1.5 mmol), EtOH (3.0 mL), and 0.07 g PAC catalysts stirred at reflux temperature in the air for 5 h.

b Isolated yields.

**Scheme 1:**
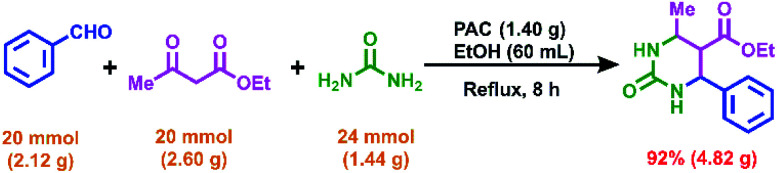
The gram-scale Biginelli reaction using the PAC catalyst.

**Scheme 2:**
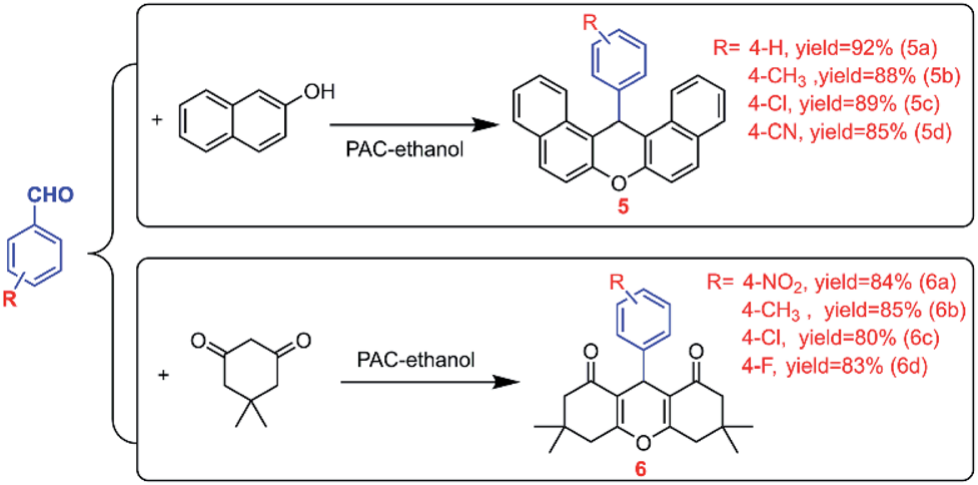
Synthesis of xanthene compounds catalyzed by PAC.

The catalytic activity of the PAC catalyst was investigated in the Biginelli reaction when scaled up to 20 mmol, and the result is shown in Scheme 1. After 8 hours of reaction, the target compound was obtained with a yield of 92%. This gratifying result, with the excellent activity of the PAC material in the gram-scale reaction, suggests the possibility of its industrial production. In addition, the application range of the PAC–ethanol catalytic system was expanded (Scheme 2), and it was found that xanthene compounds could be obtained with moderate yields. When 2-naphthol (Scheme 2, 5a–5d) or 1,3-cyclohexanedione (Scheme 2, 6a–6d) was used as the reactant, the PAC–ethanol catalytic system showed high catalytic activity. Therefore, the low-cost PAC once again proved to be a green, efficient and economic catalyst for organic synthesis reactions.

**Fig. 1:**
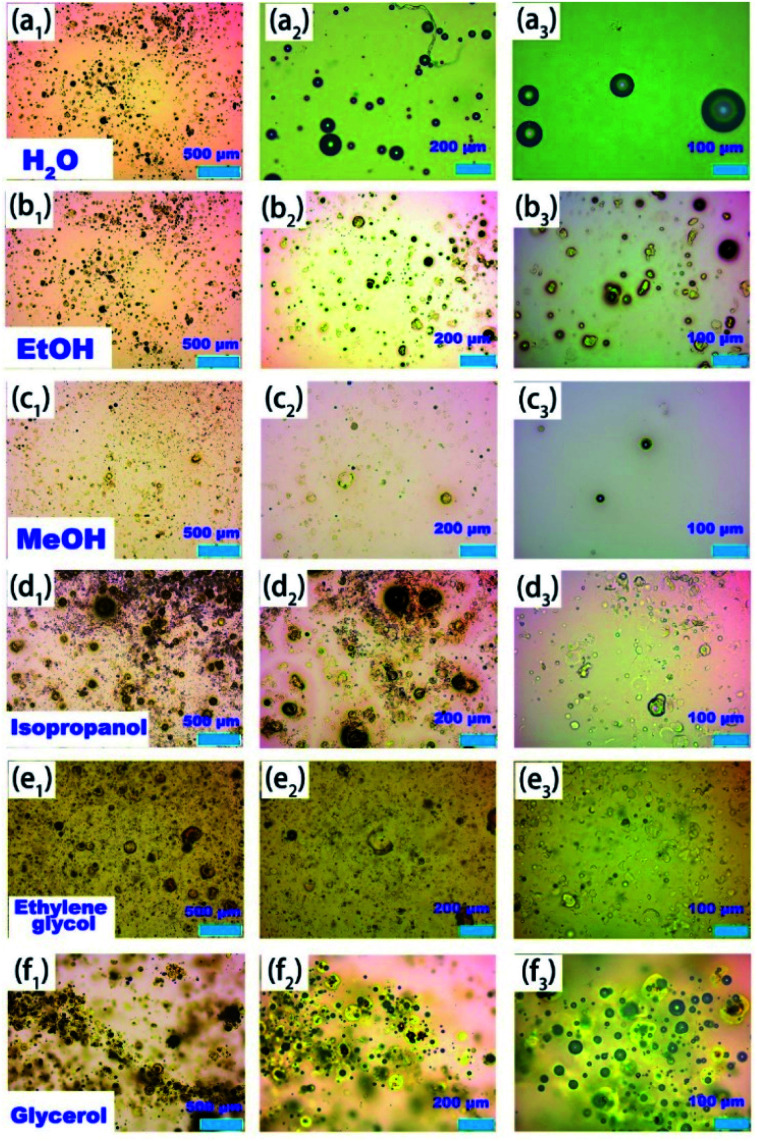
Optical micrographs of PAC in different protic solvents.

It is worth mentioning that the Al–O polynuclear cations represented by PAC exist everywhere in our lives, and we have little quantitative understanding of the way they form, dissolve, and react with other solutes. Usually, catalyst activity is closely related to the state and morphology of the catalyst in the reaction system.^39^ First of all, in order to explore the microscopic mechanism of the self-assembled Al_b_ aggregates and other particles in the PAC–ethanol system, we used an optical microscope and particle size analyzer to study the morphology of PAC in different solvents, and the results are shown in Fig. 1 and S3.†Fig. 1(a_1_–a_3_) is the optical image of PAC material in an aqueous solution; it can be seen that the PAC material first formed round vesicles after coming in contact with water, and then quickly dissolved in water. This is because PAC is hydrolyzed quickly in water, forming monomeric Al_a_ first, and then, Al_a_ self-assemble and aggregate to form Al_b_ due to the hydroxyl groups in water. However, Al_b_ is unstable in water and quickly hydrolyses to form the Al(OH)_3_ colloidal solution, and thus the active species Al_b_ would only exist for a short time. Fig. 1(b_1_–b_3_) shows the optical image of the irregular-shaped PAC material in an ethanol solution. Unlike in aqueous solutions, the dissociation rate of PAC materials in non-aqueous solvents (ethanol) is very slow. It can maintain the original structure for a long time; in other words, the active species Al_b_ can exist in the ethanol solution for a long duration, which may be one of the reasons for the higher catalytic performance of PAC materials in ethanol. During this period, the PAC material will also be connected to the alcoholic hydroxyl groups of the ethanol molecules through the Al–O bridge, thereby constructing a self-assembled supramolecular micro–nano reactor system formed *in situ* by hydrogen bonding. This way, the Al species in the micro–nano reactor can maintain high positive charges in the form of supramolecular aggregates, which can be better dispersed and stably exist in organic solvents, while also exposing more l-acid sites. In order to further explore the effect of alcoholic solvents, we studied in detail the morphology of PAC materials in different alcoholic solvents; the results are shown in Fig. 1(c–f). When ethylene glycol and glycerol were employed as solvents, the viscosity of the solution affected the self-assembly and aggregation of PAC materials. When isopropanol was selected as the solvent, unlike other alcohol solvents, the PAC materials hardly underwent hydroxylation, which meant that the main active species in the isopropanol solution was Al_a_. If only stability and environment-friendliness are considered, isopropanol may be a good choice, but its catalytic activity is still far lower than the PAC–ethanol catalytic system. Fig. S3† shows the micrographs of PAC in other aprotic solvents. It can be seen that PAC was well-dispersed in polar solvents, showing a round vesicular structure. At this time, the PAC material was basically insoluble, exhibiting only self-assembly and aggregation behaviors, and then the micro–nano reactors were formed in the solution. However, the main active species of the PAC material at this time was the monomer Al_a_. Compared with the main active species Al_b_ in the ethanol system, the catalytic activity of Al_a_ is poor. In other words, the micro–nano structure formed by the self-assembly and aggregation of PAC materials in these solvents is similar to a microcapsule reactor, which provides a favorable reactive site and localized space for catalytic reactions, especially in the PAC–ethanol catalytic system.

**Fig. 2:**
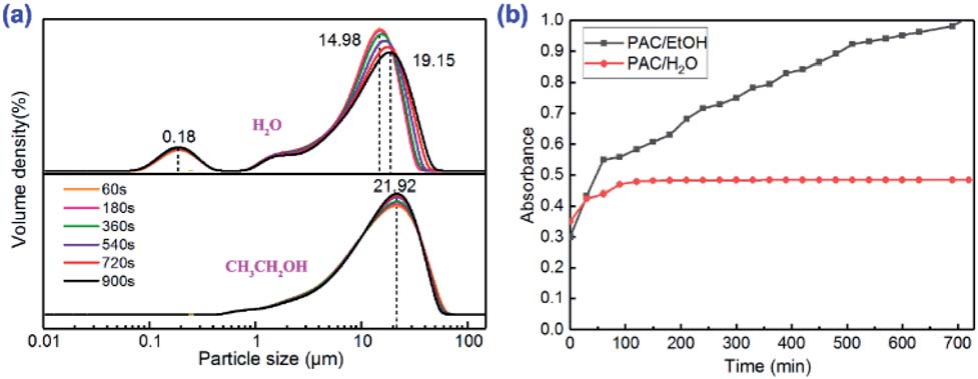
Morphological analysis of the PAC catalyst in water and the ethanol solution: (a) particle size analysis; (b) UV-Vis spectrophotometry.

Fig. 2(a) shows the results of the particle size analysis test; we can see that the particle size of the hydrolysate of PAC at the initial stage of hydrolysis was mainly around 14.98 μm, while a small number of other hydrolyzed forms of size around 0.18 μm also appeared. In the aqueous solution, with the passage of time (60–900 s), the particle size of the PAC hydrolyzate increased from 14.98 μm to 19.15 μm, which proves that PAC was extremely unstable in water.^40,41^ Due to the alcoholysis process of PAC materials in absolute ethanol, the dissolution rate of PAC in ethanol was lower than that in water; thus, in the early stage of alcoholysis, 50% of the particle size was concentrated at around 21.92 μm. As time progressed, the rate of alcoholysis slowly increased, but the particle size did not change. This phenomenon indicated that the PAC material had undergone hydroxylation and self-assembled to form a micro–nano reactor in an ethanol system and that the micro–nano reactor existed in the ethanol solution for a longer time than in the aqueous solution. Therefore, the particles formed by the self-assembly and aggregation of PAC molecules in the ethanol solution were relatively stable, which also explains the better catalytic performance of PAC in the Biginelli reaction in ethanol than that in water.

Then, we also used an ultraviolet-visible spectrophotometer to analyze the morphology of the PAC catalyst in water and ethanol by the Al–Ferron reaction,^39,42^ and the results are shown in Fig. 2(b). When water was used as the solvent, the curve became very smooth after 100 minutes of reaction. At this time, the PAC molecules in the solution no longer reacted with the Ferron reagent. It is generally believed that the form of PAC in water at this time would be Al_c_ (polymerized macromolecules or colloidal precipitates), which has poor catalytic performance. However, when PAC was first dispersed and then partially dissolved in the ethanol solution, it was found that PAC and the Ferron reagent continued to react for an extended period of time, forming a PAC–ethanol supramolecular nanoreactor through *in situ* self-assembly and aggregation under the action of hydrogen bonding. According to the literature, the PAC exists mainly in the form of Al_13_ (Al_13_O_4_(OH)_24_^7+^) during this process. Generally, Al_b_ has a much higher nuclear charge than Al_c_, and hence the catalytic effect of PAC in ethanol is much higher than that in aqueous solutions. Furthermore, we also tried to use the same method to test the morphology of PAC in other non-aqueous solvents. But unfortunately, since some of the reagents required for the reaction could not be dissolved in solvents, such as acetonitrile and DMSO, ideal experimental data were not obtained. However, based on the activity test results presented in Table 2 and the phenomenon observed by microscopy in Fig. S1,† it was inferred that PAC had different effects in different solvents. In aprotic solvents, PAC materials aggregate and self-assemble to form micro–nano reactors, but basically do not dissociate. Thus, the main active species in aprotic solvents is monomer Al_a_, and the content of polynuclear Al–O cation Al_b_ is very small. In protic solvents, PAC materials do not only form the micro–nano reactor but also dissociate to varying degrees. During this period, the new Al_b_ active species with high nuclear charges are formed, and in this state, the material has higher catalytic activity. In contrast, PAC can maintain the Al_b_ form for a long time in ethanol, due to which the PAC–ethanol catalytic system can be used as a green, cheap and efficient catalyst in the field of organic synthesis.

**Fig. 3:**
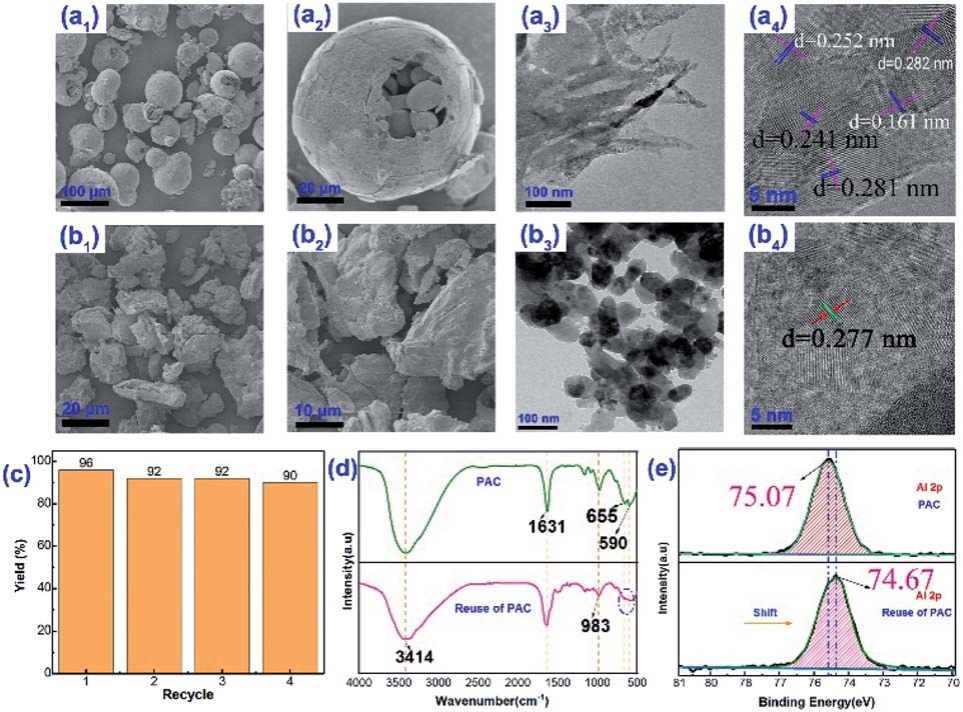
Repeated experiments and the characterization of the catalyst after use: (a) electron microscopic image of the catalyst (PAC) before use; (b) electron microscopic image of the catalyst (PAC–EtOH) after use. (c) Repeated experiments; (d) IR analysis of catalysis; (e) XPS spectra of catalysis.

In addition, the micro and nanoscale morphology of the aggregates formed by PAC and ethanol through molecular self-assembly was also revealed. The fresh PAC material presented spheres with a diameter of about 50–100 μm in the microstructure (Fig. 3a_1_), and the insides of the spheres were filled with many particles of smaller diameters. At the scale of 20 μm (Fig. 3a_2_), the sphere surface was relatively smooth. HR-TEM showed that the PAC material not only had multiple interfaces at the nanoscale (100 nm), and the active components were not only closely cross-linked together but also evenly distributed on the surface, forming lattice fringes with different crystal plane spacings (*d* = 0.161–0.282 nm). This is consistent with the literature that Al_13_ is a highly charged species formed by the dissociation of PAC material, with a molecular size of 1–2 nm.^43^ However, in the PAC–ethanol system, the shape of the PAC material had changed significantly, from spheres to a denser irregular layered block with many folds on the surface (Fig. 3b_1_ and b_2_). This meant that the hydrogen-bond-dominated PAC–ethanol supramolecules were generated through *in situ* self-assembly. In addition, HR-TEM showed that the ethanol molecules had a certain dissociation effect on the PAC material, and many small spheres with diameters between 20–50 nm appeared. At the same time, due to the solvation of alcohols (rather than hydrolysis), the degree of crosslinking between the PAC materials was relatively weak. This was caused by the partial hydroxylation of the Al–O–Al bridge structure; the bound water molecules in the PAC material were replaced by partial alcohol molecules.^4^ Nevertheless, various aluminum species were still uniformly distributed on the surface of the PAC material (Fig. 3b_4_), showing a single lattice fringe (*d* = 0.277 nm).^44^ The above changes were attributed to the self-assembly and aggregation of PAC in the ethanol system. In summary, after the alcoholysis of PAC material in the ethanol system, a cationic polymer-based solid acid catalyst was *in situ* generated. The PAC–ethanol nanoreactor was composed of Al_b_ with a high nuclear charge due to hydrogen bonding, and Al_b_ was composed of particles with a molecular size of 1–2 nm, such as Al_2_(OH)^5+^, Al_2_(OH)_2_^4+^, Al_3_(OH)_4_^5+^, Al_4_(OH)_8_^4+^, Al_6_(OH)_12_^6+^, Al_13_O_4_(OH)_24_^7+^, Al_8_(OH)_20_^4+^, *etc.* It could have a variety of active species and reaction sites and is relatively stable in the ethanol solution because of the partial hydroxylation of the Al–O–Al bridge structure.

After confirming that the PAC materials mainly exist in the form of Al_b_ in non-aqueous solvents, we began to focus on the reusability of PAC materials by observing the changes before and after use. From Fig. 3(c), it can be seen that the PAC catalyst could be reused at least 4 times in the Biginelli reaction, and the yield was still maintained at 90% of the original, which once again confirmed that the PAC material had good stability in non-aqueous solvents and could be used for a long time. Unfortunately, a very small amount of PAC material still underwent alcoholysis in the ethanol solvent, with a loss of 5–10 wt% each time. But PAC is usually used as a water purifier because it is a green, non-toxic, and environment-friendly reagent.^45^ Hence, a small amount of loss in the solution will not cause environmental pollution. Fig. 3(d) shows the infrared spectra of the catalyst before and after use.^46^ The peaks at 3414 cm^−1^ and 1631 cm^−1^ represented the vibration of the –OH structures in the PAC material, and the PAC material did not change significantly before and after the reaction.^47^ The absorption peaks of the material at 655 cm^−1^ and 590 cm^−1^ after use were not as sharp as before, and the two peaks even overlapped. This may be caused by the partial hydroxylation of the Al–O–Al structure during use.^42^

The changes in the surface elemental composition of the catalytically active species also proved the above conclusions. Fig. 3(e) shows the XPS spectrum of the Al 2p orbital in the PAC material; it can be seen that the Al 2p orbital shifted after use, with the peak center moving from 75.07 eV to 74.67 eV. The possible reason is the bridging of the PAC material with ethanol molecules during the reaction. The alcohol molecules partially replaced the hydroxyl groups in the original structure, and the Al 2p orbital electron density slightly increased. This indicated that the crystalline structure of the PAC material had changed during use. In other words, the PAC materials formed supramolecular nanoreactors *in situ* through alcoholysis, self-assembly and aggregation.

The above findings show that the PAC–ethanol system facilitates the easy *in situ* construction of a micro–nano structure reactor when catalyzing organic synthesis reactions. This structure is mainly composed of Al–O cations with different hydroxylated Al_b_ structures in the ethanol solution, including Al_2_(OH)^5+^, Al_2_(OH)_2_^4+^, Al_3_(OH)_4_^5+^, Al_4_(OH)_8_^4+^, Al_6_(OH)_12_^6+^, Al_13_O_4_(OH)_24_^7+^, Al_8_(OH)_20_^4+^, *etc.* These hydroxylated cationic aggregates of different structures have a special synergistic catalytic effect on the synthesis of pyrimidinone, bis-indole methane and xanthene compounds. Although the morphology and surface nanostructure of the PAC material changes during use, it does not change significantly as the key active component of solid acid catalysis. Therefore, the PAC catalyst still maintains high catalytic activity after repeated use four times. The stability and exceptional catalytic performance of PAC materials in non-aqueous solvents (ethanol) may provide unlimited possibilities for their applications in the field of organic synthesis.

**Fig. 4:**
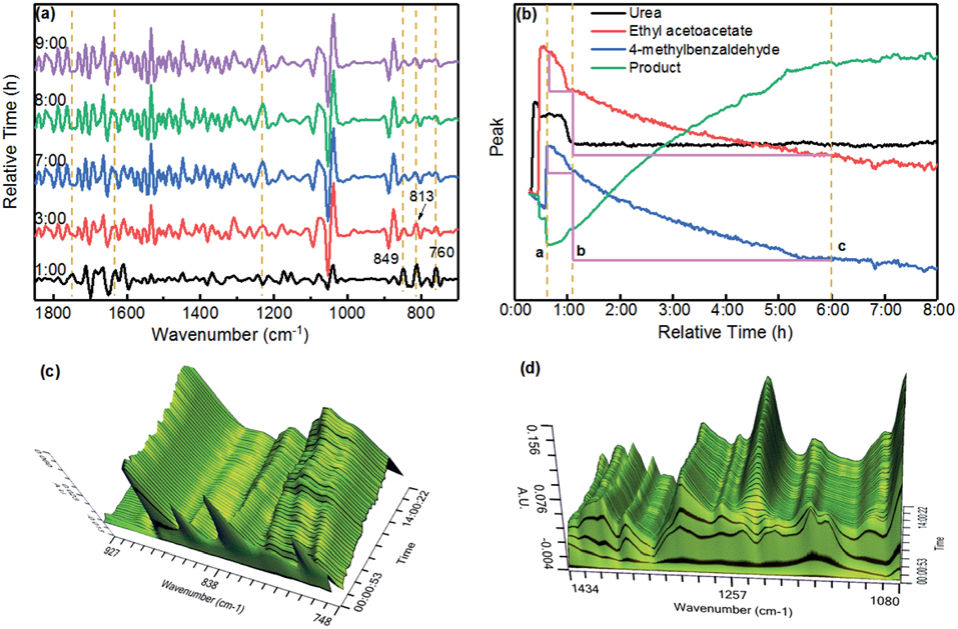
Infrared *in situ* monitoring of the Biginelli reaction in the PAC–ethanol catalytic system.

It is important to note that, at present, there is some controversy about the mechanism of the Biginelli reaction; because different types of catalysts and reaction substrates are combined in different ways, the sequence and progress of the reaction may be different. In order to get a clearer understanding of the catalytic mechanism of the PAC catalyst in the Biginelli reaction, we used an *in situ* infrared monitoring instrument to monitor the reaction process, and the results are shown in Fig. 4. Fig. 4(a) shows the *in situ* online infrared spectra of the Biginelli reaction at different time periods. It was found that the characteristic peaks of the three reaction substrates gradually weakened with the extension of the reaction time (ethyl acetoacetate: 1631 cm^−1^, urea: 1748 cm^−1^, 4-methylbenzaldehyde: 849–760 cm^−1^). At the same time, the characteristic peak of the reaction product gradually increased (1231 cm^−1^).

As shown in Fig. 4(b), after transforming the data, the change curves of the reaction substrates and product were obtained. At the time (a) in the figure, all the raw materials had been added and heated to the reflux temperature. It can be seen from the figure that the product was formed immediately. During the reaction, the content of urea first decreased (a–b) and then remained steady (b–c). This proved that urea was first rapidly consumed during the reaction. Because the amount of urea was excessive (1.5 eq.), the content did not decrease in the late stage of the reaction. However, the content of 4-methylbenzaldehyde and ethyl acetoacetate continuously decreased and stabilized after about 5 hours of reaction, which meant that the reaction was over. In addition, we could more intuitively observe the changing trend of the peaks at 748–927 cm^−1^, 1080 cm^−1^ and 434 cm^−1^ with time from the three-dimensional spectra shown in Fig. 4(c) and (d). They proved that in this reaction system, urea was first activated to participate in the reaction. In addition, through linear simulation in the METTLER TOLEDO ReactIRTM iC^10^ software, we found that 4-methylbenzaldehyde had a faster reaction rate than ethyl acetoacetate (the peak value of 4-methylbenzaldehyde decreased more during the b–c period), and the above reaction process conformed to the Iminium mechanism. In a nutshell, the *in situ* infrared apparatus provided an intuitive and reliable basis for reasonably inferring the mechanism of the Biginelli reaction. It could be determined that the reaction process involved urea reacting with 4-methylbenzaldehyde first to form an intermediate, which then undergoes cyclization with ethyl acetoacetate.

**Fig. 5:**
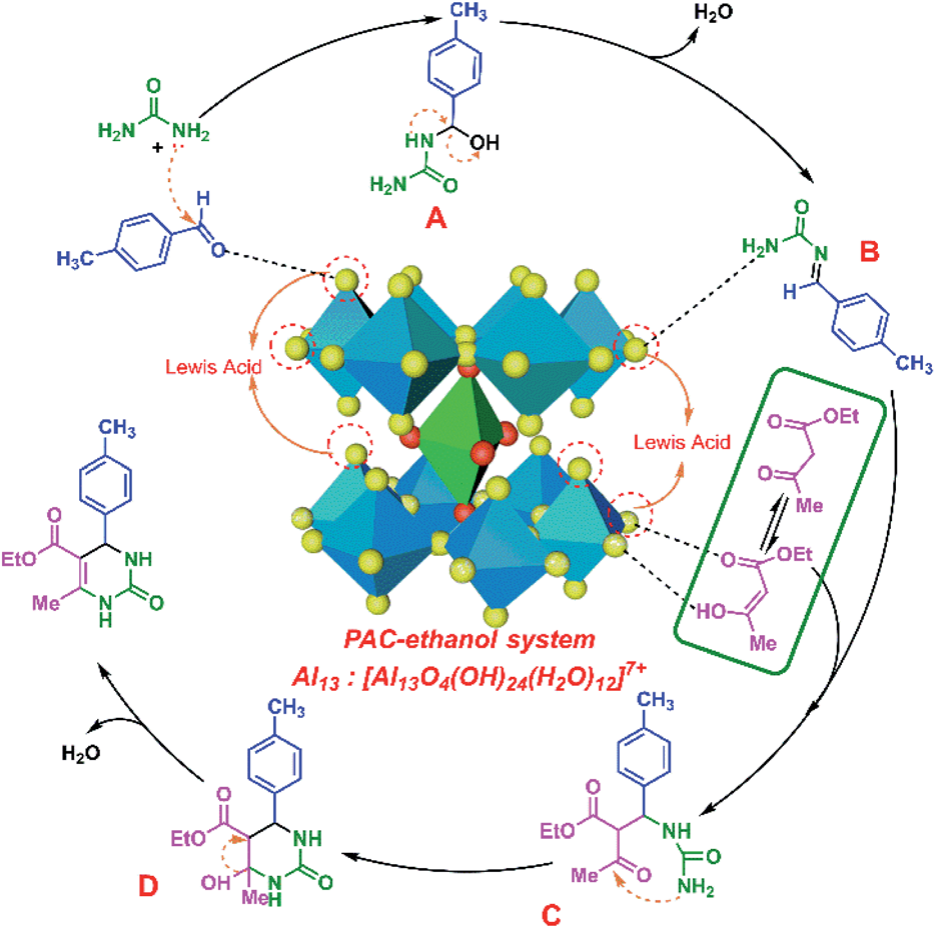
Mechanism of the Biginelli reaction in the PAC–ethanol catalytic system.

On the basis of the above catalysis and structural analyses, a possible cooperative catalytic mechanism for the Biginelli reaction has been proposed, as illustrated in Fig. 5. Firstly, urea and 4-methylbenzaldehyde undergo a nucleophilic addition reaction to form intermediate A. Then, a molecule of water is removed from intermediate A to form intermediate B. At this point, the protons in urea are captured by the active species Al_b_, while the carbonyl group of ethyl acetoacetate is also activated effectively by the metal Al ions in the catalyst, and the two react to form intermediate C. The amino group in intermediate C undergoes intramolecular electrophilic addition with the carbonyl group to form intermediate D. The hydroxyl group in molecule D is structurally unstable and reacts with adjacent carbon atoms to remove one molecule of H_2_O. After that, the final target compound pyrimidine is obtained.^35^

Overall, a comparative study of PAC with some of the reported catalysts for the synthesis of the pyrimidinone compound was performed (Table 4). Compared with the same type of Al catalysts and other Lewis acid metal salt catalysts, the PAC–ethanol system showed some advantages, such as (i) a wide range of sources, low price, and environment-friendliness; (ii) ease of handling and product separation; (iii) *in situ* generation of the metal-containing micro–nano reactor catalyst by a simple, green process (with ethanol as the only solvent); (iv) negligible negative effects despite the PAC material showing a small amount of metal loss in the ethanol system; (v) ease of visualization of the reaction. Therefore, we believe that PAC, as a green, new and efficient Lewis acid catalyst, will have very extensive applications in the field of organic synthesis in the future.

**Table 4:** Comparative study of the results of the PAC catalyst in the Biginelli reaction with those of other catalysts

Entry	Catalyst	Condition	Reuse (times)	Yield (%)	Ref.
1	PANI-AlCl_3_	MeOH, reflux	4	97	48
2	Al-MCM-41	Octane, 110 °C	5	92	49
3	ZrO_2_–Al_2_O_3_–Fe_3_O_4_	Ethylene glycol, 140 °C	6	82	50
4	Bi_2_ZnAl_2_O_9_	Solvent-free, 80 °C	3	94	51
5	Nb_2_O_5_	Solvent-free, 130 °C	—	70	52
6	Chymotrypsin	EtOH, 55 °C	—	92	53
7	ompg-C_3_N_4_/SO_3_H	EtOH, reflux	4	98	54
8	Mag@MorPh-AIL	EtOH, reflux	8	93	55
9	Co/Al-SG	Solvent-free, 100 °C	3	71	56
10	3D printed-Al_2_O_3_	Solvent-free, MW, 120 °C	10	94	57
11	PANI-FeCl_3_	CH_3_CN, reflux	—	90	58
12	PAC	EtOH, reflux	4	99	This work

## Experimental details

### The typical procedure for the Biginelli reactions using PAC catalysts

To a 15 mL reaction tube, benzaldehyde (1.0 mmol), acetyl methyl acetate (1.0 mmol), urea (1.5 mmol), ethanol (3.0 mL) and 0.07 g PAC were added. The reaction mixture was stirred at 100 °C, and TCL was used to monitor the progress of the reaction. After the reaction, the catalyst was isolated by filtration with a 0.45 μm membrane, washed with hot ethyl acetate, vacuum-dried at 40 °C and then reused in the next round of reaction. The organic phase was collected, and the pure Biginelli products were obtained after recrystallization in ethanol.

### Method for determination of PAC morphology in different solvents

Al–Ferron reaction:^37^ to a 250 mL volumetric flask, Ferron (0.2500 g), 1,10-phenanthroline (0.0125 g), sodium acetate (17.500 g), hydroxylamine hydrochloride (5.0000 g), and HCl (2.0 mL, 6 mol L^−1^) were added, and buffer solution A was obtained by diluting to a constant volume. Then, 0.400 g PAC was accurately weighed, dissolved and diluted in a 100 mL volumetric flask, which was marked as solution B.

To a 200 mL volumetric flask, solution A (40 mL) and solution B (8 mL) were added, and the pH was adjusted to 5.2 to get the test solution C. Every 25 minutes, 2 mL of solution C was withdrawn and tested in a UV-Vis spectrophotometer (H_2_O: 370 nm, EtOH: 245 nm). The change in absorbance value was recorded over time, and a curve was drawn.

### Experimental method for the *in situ* infrared monitoring reaction

A 100 mL three-necked flask was charged with absolute ethanol (20 mL). With magnetic stirring turned on, the *in situ* infrared instrument started collecting data from the solution. When heated to 100 °C, 4-methylbenzaldehyde (10 mmol), acetyl methyl acetate (10 mmol), urea (15 mmol) and the catalyst (700 mg) were added, respectively. After the reaction was completed, data collection was stopped, and the data were processed using the METTLER software and the Origin software provided with the instrument.

## Conclusions

In summary, the morphological characteristics of PAC materials in different solvents have been investigated in detail by using optical microscopy, UV-Vis spectrophotometry, particle size analysis, XPS, IR, SEM and HR-TEM. First of all, in the ethanol solvent, PAC mainly transformed *in situ* into a new solvation polynuclear cationic polymer molecule Al_b_ (made of Al_2_(OH)^5+^, Al_2_(OH)_2_^4+^, Al_3_(OH)_4_^5+^, Al_4_(OH)_8_^4+^, Al_6_(OH)_12_^6+^, Al_13_O_4_(OH)_24_^7+^, Al_8_(OH)_20_^4+^, *etc.*). Then, these cationic ethanoate aggregates formed a self-assembled supramolecular micro–nano reactor system *in situ via* hydrogen bonding. Afterward, the polyaluminum chloride micro–nano reactor exhibited excellent catalytic activity (in 3 types of reactions, the best yield = 99%) and stability (yield >90% after repeated use for 4 times) in organic synthesis reactions. In addition, the Biginelli reaction catalyzed by the PAC–ethanol system was monitored by an *in situ* infrared apparatus, and the kinetic changes in the reaction were analyzed. The catalytic mechanism of the PAC–ethanol system in the Biginelli reaction was confirmed by the rate of raw material consumption, and it is believed that this process is more consistent with the Iminium mechanism pathway. Compared with other types of transition metal salt catalysts, PAC materials are easier to handle, more efficient, green, environment-friendly and less priced. The catalytic application of the PAC material in green organic synthesis, as reported in this article, will bring a new life to this cheap inorganic polymer material, which is widely used in sewage treatment.

## Conflicts of interest

There are no conflicts to declare.

## Supplementary Material

RA-012-D1RA08038E-s001
