# Supplementary material for: The new life of traditional water treatment flocculant polyaluminum chloride (PAC): a green and efficient micro–nano reactor catalyst in alcohol solvents
Source: RSC Adv. 2021 Dec 23;12(2):655–63. doi: 10.1039/d1ra08038e (PMC8696963; doi:10.1039/d1ra08038e)
Supplement: RA-012-D1RA08038E-s001 [file RA-012-D1RA08038E-s001.pdf]

Electronic Supplementary Material (ESI) for RSC Advances.
This journal is © The Royal Society of Chemistry 2021

Supporting Information

A new life of traditional water treatment flocculant polyaluminum chloride (PAC): a green and efficient micro-nano reactor catalyst in alcohol solvents

Gang Wang, Pengcheng Hao, Yanping Liang, Yuwang Liang, Wanyi Liu*, Jiantong Wen, Xiang Li, Haijuan Zhan, Shuxian Bi

State Key Laboratory of High-efficiency Utilization of Coal and Green Chemical Engineering, National Demonstration Center for Experimental Chemistry Education, College of Chemistry and Chemical Engineering, Ningxia University, Yinchuan 750021, P. R. China

Corresponding author:

Wanyi Liu, State Key Laboratory of High-efficiency Utilization of Coal and Green Chemical Engineering, College of Chemistry and Chemical Engineering, Ningxia University, Yinchuan 750021, People's Republic of China. Email: liuwy@nxu.edu.cn.

1 General experimental condition

1.1 Chemicals and instruments

All reagents are more than 98% pure and the solvent is A.R. It is used directly after purchase. Polyaluminium chloride (PAC) is golden yellow powder, the content of alumina is about 30%, in accordance with the GB15892-2003 standard, China henan runquan purification materials co, LTD. The samples are produced by the calcium aluminate adjustment method. Scanning electron microscope (SEM) image recorded on the Hitachi S4800. The morphology of the materials was obtained on JEOL JEM-2010F High Resolution Transmission Electron Microscope (HRTEM). FTIR spectral analysis by Bruker TENSOR 27Two03040404. 1H and 13C NMR were obtained by Bruker 400 MHz spectrometer. The powder X-ray diffraction (XRD) patterns were recorded by a D/max 2200 PC03030502 X-ray diffractometer using Cu-Karadiation. The X-ray photoemission (XPS) spectra were performed at room temperature using a Thermo ESCALAB 250xi hemispherical electron energy analyzer. The online infrared spectrum was obtained by METTLER TOLEDO ReactIRTM iC10.

1.2 Typical procedure for the Biginelli reactions by PAC catalyst

To a 15 mL reaction tube, Benzaldehyde (1.0 mmol), Acetyl methyl acetate (1.0 mmol), Urea (1.5 mmol), Ethanol (3.0 mL) and 0.07 g PAC were added. The reaction mixture was stirred at 100℃, use TCL to monitor the progress of the reaction. After the reaction, the catalyst was isolated by filtration with 0.45μm membrane, washed by hot ethyl acetate, vacuum dried at 40℃ and then reused in the next round of reaction. Organic phase was collected, the pure Biginelli products were obtained after recrystallization in ethanol.

1.3 Typical procedure for synthesis of 5a and 5b compounds by PAC catalyst

To a 15 mL reaction tube, 2-naphthol (1 mmol)，aromatic aldehyde(0.5 mmol). Ethanol (3.0 mL) and 0.07 g PAC were added. The reaction mixture was stirred at 90℃, use TCL to monitor the progress of the reaction. After the reaction, the catalyst was isolated by filtration with 0.45μm membrane, washed by hot ethyl acetate, vacuum dried at 40℃. Organic phase was collected, the pure products were obtained after recrystallization in ethanol.

1.4 Typical procedure for synthesis of 6a, 6b and 6c compounds by PAC catalyst

To a 15 mL reaction tube, 4-nitrobenzaldehyde (1.0 mmol), 5,5-Dimethyl-1,3-

cyclohexanedione (2.0 mmol). Ethanol (3.0 mL) and 0.07 g PAC were added. The reaction mixture was stirred at 70℃, use TCL to monitor the progress of the reaction. After the reaction, the catalyst was isolated by filtration with 0.45μm membrane, washed by hot ethyl acetate (3*5 mL), vacuum dried at 40℃. Organic phase was collected, the pure products were obtained after recrystallization in ethanol.

1.4 Method for determination of PAC morphology in different solvents

To a 250 mL volumetric flask, Ferron (0.2500 g), 1,10-Phenanthroline (0.0125 g), Sodium acetate (17.500 g), Hydroxylamine hydrochloride (5.0000 g), HCl (2.0 mL, 6 mol/L) were added, the buffer solution A was obtained by diluting and constant volume. Then the 0.400 g PAC was accurately weighed, dissolved and diluted into a 100 mL volumetric flask, marked as solution B. To a 200 mL volumetric flask, solution A (40 mL) and solution B (8 mL) were added, adjust pH to 5.2 to get test solution C. Take 2 mL of solution C every 25 minutes and test in UV-Vis spectrophotometer (H_2O: 370 nm, EtOH: 245 nm), Record the change of absorbance value over time and draw a curve.

1.5 Experimental method for in situ infrared monitoring reaction

A 100 mL three-necked flask was charged with absolute ethanol (20 mL). Turn on magnetic stirring and the in-situ infrared instrument and start collecting data. When heat to 100℃, 4-methylbenzaldehyde (10 mmol), Acetyl methyl acetate (10 mmol), Urea (15 mmol) and Catalyst (700 mg) were began to added respectively. After the reaction was completed, the data collection was stopped and the data was processed using the METTLER software and the Origin software that came with the instrument.

2. Experimental data

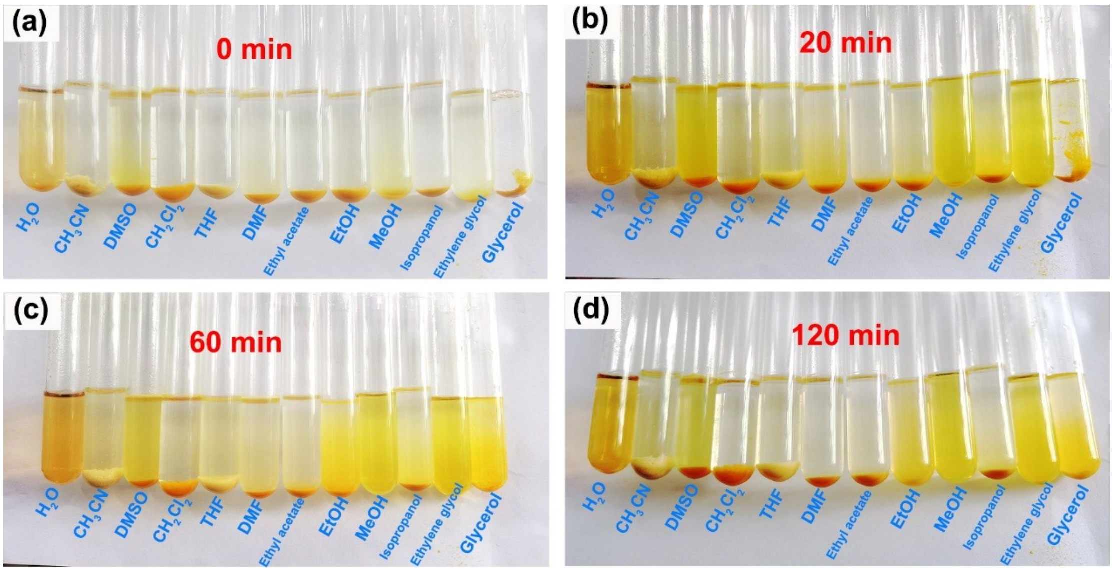

Figure S1. The Dissolution image of PAC in different solvents

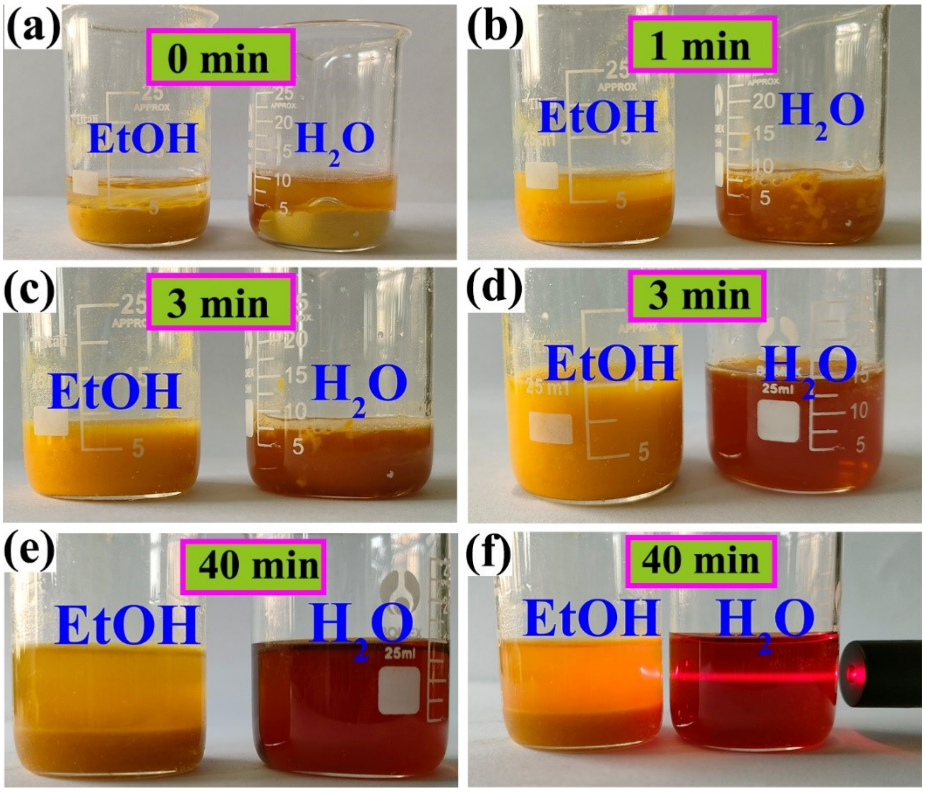

Figure S2. The dissolution images of PAC material in ethanol and water

The above figure shows the comparison pictures of the dissolution status of PAC materials in water and ethanol within 0-3min. Add the same amount of PAC material (5 cm^3) in the two beakers, add an equal volume of solution (5 mL), and observe the dissolution status. As shown in the figure (a-c), it can be found that the PAC material is almost completely dissolved in the aqueous solution within 3 minutes, but only dispersed (suspended) in the ethanol solution. In order to get a more obvious result, add 5 mL of the solution to the beaker again, as shown in Figure (d). At this time, it can be clearly seen that the solution in the water is basically translucent, and the ethanol is still very turbid. After that, the solution was allowed to stand for 40 minutes (Figure (e)). Obvious precipitate appeared in the ethanol, which was divided into two layers of solid and liquid, but the aqueous solution was still transparent. Through the detailed observation, it can be found that the state of the PAC material in ethanol has changed, and the material has become fluffier and more cross-linked. This indicates that the PAC material acts on the solvent ethanol molecules to form multi-nuclear Al-O Cation ($Al_2(OH)^{5+}$, $Al_2(OH)_2{}^{4+}$, $Al_3(OH)_4{}^{5+}$, $Al_4(OH)_8{}^{4+}$, $Al_6(OH)_{12}{}^{6+}$, $Al_{13}O_4(OH)_{24}{}^{7+}$, $Al_8(OH)_{20}{}^{4+}$) through self-organization and aggregation, the same result is confirmed

in XPS and SEM. In addition, using infrared laser to irradiate the solution, the Tyndall effect appeared in the aqueous solution, but it did not appear in the ethanol (Figure (f)). This indicates that PAC is dissolved in water to produce a colloidal solution, while in ethanol it will undergo complex processes such as self-assembly and aggregation. The polynuclear Al-O cation formed in ethanol is completely different from the hydrolysis in aqueous solution. In ethanol solution, PAC material can better maintain the characteristics of Lewis acid, so it has high catalytic activity in ethanol solution.

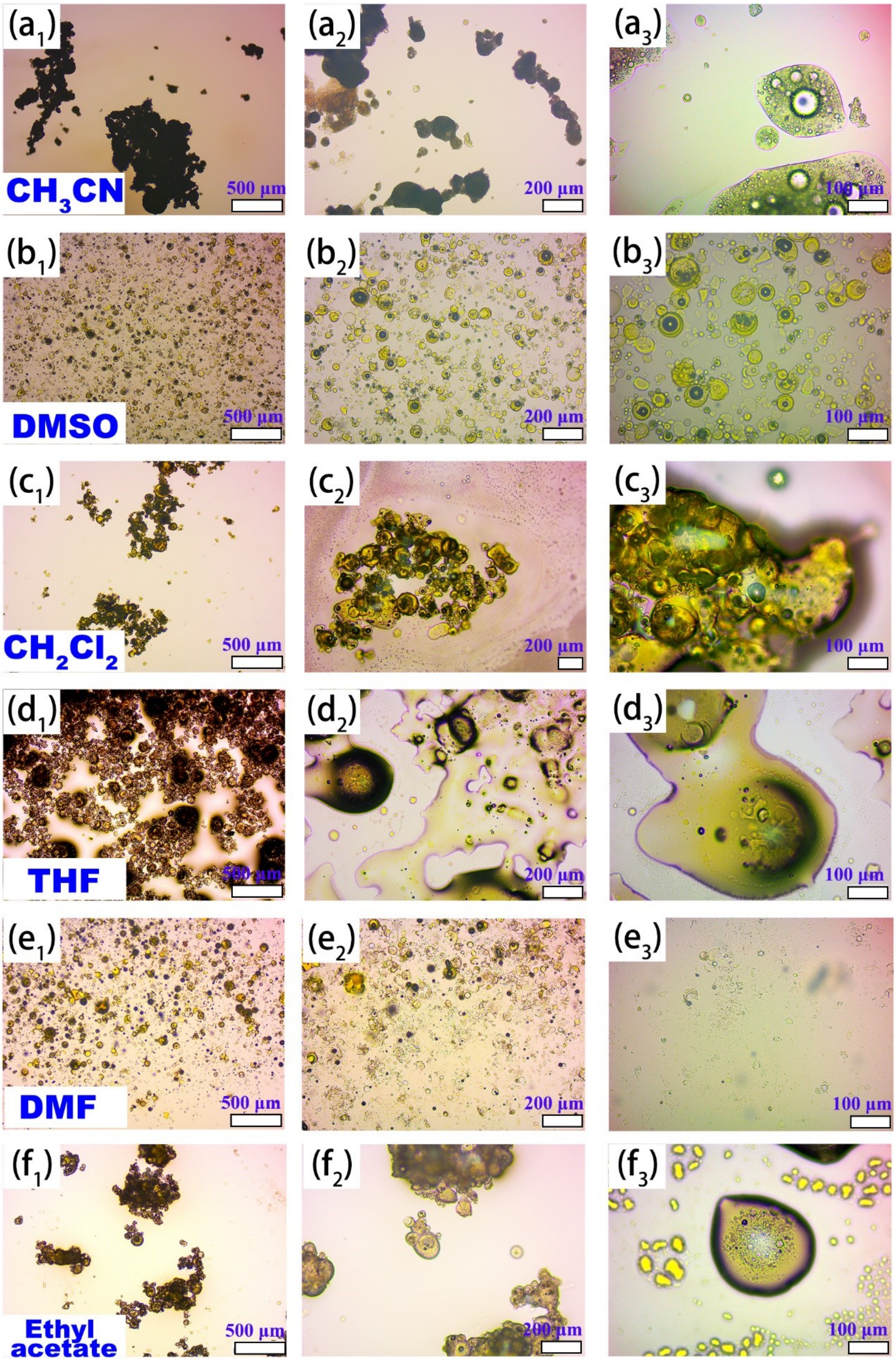

Figure S3. Optical micrographs of PAC in different aprotic solvents

3. Characterization data of the products

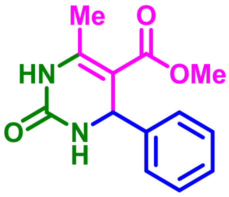

methyl 6-methyl-2-oxo-4-phenyl-1,2,3,4-tetrahydropyrimidine-5-carboxylate (Table 2, 4a): White solid, 99%. ^{1}H NMR (400 MHz, DMSO) δ9.27, 7.81, 7.81, 7.81, 7.79, 7.78, 7.35, 7.34, 7.33, 7.33, 7.32, 7.32, 7.31, 7.30, 7.25, 7.25, 7.24, 7.23, 7.23, 5.34, 5.33, 5.16, 5.15, 3.55, 3.52, 2.26. ^{13}C NMR (101 MHz, DMSO) δ 166.00, 152.38, 148.86, 144.82, 128.61, 127.46, 126.34, 99.15, 53.93, 50.95, 18.00. known compound [1].

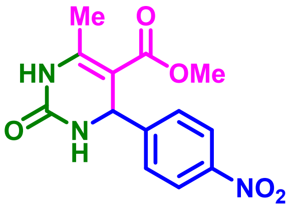

methyl 6-(2-chloro-4-nitrophenyl)-4-methyl-2-oxo-1,2,3,4-tetrahydropyrimidine-5-carboxylate (Table 2, 4b): Yellow solid, 80%. ^{1}H NMR (400 MHz, DMSO-d6) δ 9.42 (s, 1H), 8.22 (m, 2H), 7.93 (m, 1H), 7.51 (m, 2H), 5.27 (d, J = 3.4 Hz, 1H), 3.53 (s, 2H), 2.27 (s, 3H). ^{13}C NMR (101 MHz, DMSO) δ 165.73, 151.98, 151.96, 149.82, 146.88, 127.75, 124.05, 98.11, 53.68, 51.09, 18.09. known compound [1].

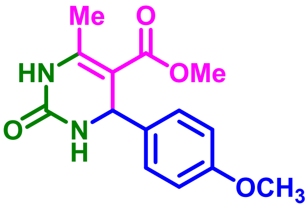

methyl 6-(2-chloro-4-methoxyphenyl)-4-methyl-2-oxo-1,2,3,4-tetrahydropyrimidine-5-carboxylate (Table 2, 4c): White solid, 72%.^{1}H NMR (400 MHz, DMSO-d6) δ 9.21 (s, 1H), 7.71 (s, 1H), 7.13 (d, J = 8.1 Hz, 2H), 6.87 (d, J = 8.1 Hz, 2H), 5.08 (d, J = 3.4 Hz, 1H), 3.71 (s, 3H), 3.52 (s, 3H), 2.24 (s, 3H). ^{13}C NMR (100 MHz, DMSO) δ 166.31, 158.89, 152.62, 148.84, 137.28, 127.85, 127.79, 114.21, 114.14, 99.68, 55.51, 53.61, 51.26, 18.28. known compound [2].

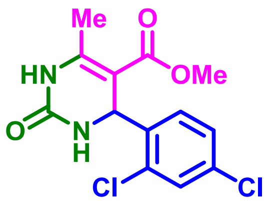

methyl 6-(2,4-dichlorophenyl)-4-methyl-2-oxo-1,2,3,4-tetrahydropyrimidine-5-carboxylate (Table 2, 4d): White solid, 55%. ^{1}H NMR (400 MHz, DMSO-d6) δ 9.38 (s, 1H), 7.80 (s, J = 2.6 Hz, 1H), 7.57 (d, J = 2.2 Hz, 1H), 7.41 (dd, J = 8.4, 2.1 Hz, 1H), 7.31 (dd, J = 8.4, 2.4 Hz, 1H), 5.58 (d, J = 3.1 Hz, 1H), 3.45 (s, 3H), 2.29 (s, 3H). ^{13}C NMR (100 MHz, DMSO-d6) δ 165.79, 151.68, 151.60, 150.17, 141.24, 133.05, 132.99, 130.62, 129.24, 129.12, 128.45, 97.76, 51.50, 51.24, 18.24, 18.15. known compound [1].

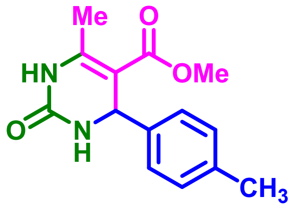

methyl 4-methyl-2-oxo-6-(p-tolyl)-1,2,3,4-tetrahydropyrimidine-5-carboxylate (Table 2, 4e): White solid, 85%. ^{1}H NMR (400 MHz, DMSO-d6) δ 9.22 (s, 1H), 7.74 (p, J = 1.9 Hz, 1H), 7.11 (s, 4H), 5.10 (t, J = 2.5 Hz, 1H), 3.51 (s, 3H), 2.25 (d, J = 3.2 Hz, 6H). ^{13}C NMR (100 MHz, DMSO) δ 166.37, 152.74, 149.01, 142.23, 136.97, 129.49, 126.60, 99.62, 53.98, 51.31, 18.33. known compound [1].

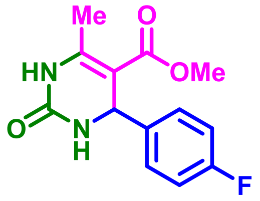

methyl 6-(4-fluorophenyl)-4-methyl-2-oxo-1,2,3,4-tetrahydropyrimidine-5-carboxylate (Table 2, 4f): White solid, 80%. ^{1}H NMR (400 MHz, DMSO-d6) δ 9.28 (s, 1H), 7.80 (dd, J = 3.5, 2.0 Hz, 1H), 7.25 (m, 2H), 7.15 (m, 2H), 5.14 (d, J = 3.4 Hz, 1H), 3.52 (s, 3H), 2.25 (s, 3H). ^{13}C NMR (100 MHz, DMSO) δ 166.21, 152.49, 149.32, 141.38, 141.35, 128.68, 128.60, 115.78, 115.57, 99.30, 53.59, 51.33, 18.32. known compound [2].

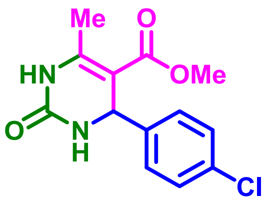

ethyl 6-(4-chlorophenyl)-4-methyl-2-oxo-1,2,3,4-tetrahydropyrimidine-5-carboxylate (Table 2, 4g): White solid, 85%. ^{1}H NMR (400 MHz, DMSO-d6) δ 9.28 (s, 1H), 7.81 (m, 1H), 7.39 (m, 2H), 7.26 (m, 2H), 5.14 (d, J = 3.3 Hz, 1H), 3.97 (q, J = 7.1 Hz, 2H), 2.25 (s, 3H), 1.09 (t, J = 7.1 Hz, 3H). ^{13}C NMR (100 MHz, DMSO) δ 165.64, 152.39, 149.20, 144.22, 132.23, 128.85, 128.64, 99.23, 59.73, 18.26, 14.52. known compound [2].

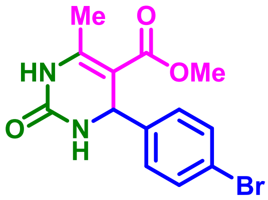

methyl 6-(4-bromophenyl)-4-methyl-2-oxo-1,2,3,4-tetrahydropyrimidine-5-carboxylate (Table 2, 4h): White solid, 82%. ^{1}H NMR (400 MHz, DMSO-d6) δ 9.31 (s, 1H), 7.82 (dd, J = 3.5, 2.0 Hz, 1H), 7.57 – 7.48 (m, 2H), 7.24 – 7.14 (m, 2H), 5.13 (d, J = 3.4 Hz, 1H), 3.52 (s, 3H), 2.25 (s, 3H). ^{13}C NMR (100 MHz, DMSO) δ 166.12, 152.40, 149.46, 144.42, 131.81, 128.91, 120.80, 98.93, 53.76, 51.28, 18.30. known compound [3].

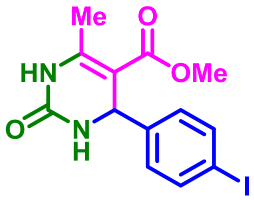

methyl 4-(4-iodophenyl)-6-methyl-2-oxo-1,2,3,4-tetrahydropyrimidine-5-carboxylate (Table 2, 4i): White solid, 87%. ^{1}H NMR (400 MHz, DMSO-d6) δ 9.30 (s, 1H), 7.84 – 7.78 (m, 1H), 7.69 (d, J = 8.2 Hz, 2H), 7.05 (d, J = 8.3 Hz, 2H), 5.11 (d, J = 3.4 Hz, 1H), 3.52 (s, 3H), 2.25 (s, 3H). ^{13}C NMR (100 MHz, DMSO) δ 165.83, 152.14, 149.14, 144.53, 137.38, 128.77, 98.62, 93.48, 53.60, 51.02, 18.04. known compound [2]

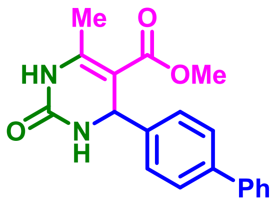

methyl 6-([1,1'-biphenyl]-4-yl)-4-methyl-2-oxo-1,2,3,4-tetrahydropyrimidine-5-carboxylate (Table 2, 4j): White solid, 75%. ^{1}H NMR (400 MHz, DMSO-d6) δ 9.30 (s, 1H), 7.84 (dd, J = 3.5, 2.0 Hz, 1H), 7.67 – 7.58 (m, 4H), 7.45 (t, J = 7.5 Hz, 2H), 7.38 – 7.35 (m, 1H), 7.32 (d, J = 8.3 Hz, 2H), 5.19 (d, J = 3.4 Hz, 1H), 3.55 (s, 3H), 2.28 (s, 3H). ^{13}C NMR (100 MHz, DMSO) δ 166.28, 152.64, 149.27, 144.19, 140.30, 139.70, 129.35, 127.84, 127.31, 127.27, 127.21, 127.08, 99.29, 53.90, 51.32, 18.34. known compound [4].

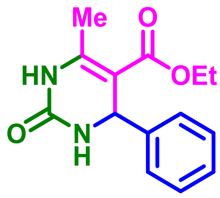

ethyl 4-methyl-2-oxo-6-phenyl-1,2,3,4-tetrahydropyrimidine-5-carboxylate (Table 2, 4k): White solid, 90%. ^{1}H NMR (400 MHz, DMSO-d6) δ 9.23 (s, 1H), 7.77 (dd, J = 3.5, 2.0 Hz, 1H), 7.36 – 7.29 (m, 2H), 7.24 (ddt, J = 7.9, 3.2, 1.8 Hz, 3H), 5.14 (d, J = 3.3 Hz, 1H), 3.98 (q, J = 7.1 Hz, 2H), 2.25 (s, 3H), 1.09 (t, J = 7.1 Hz, 3H). ^{13}C NMR (100 MHz, DMSO) δ 165.77, 152.59, 148.83, 145.30, 128.85, 127.72, 126.69, 99.65, 59.65, 54.39, 18.24, 14.52. known compound [1].

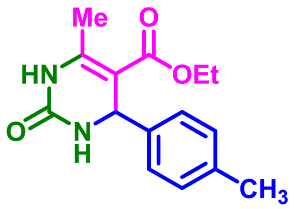

ethyl 4-methyl-2-oxo-6-(p-tolyl)-1,2,3,4-tetrahydropyrimidine-5-carboxylate (Table 2, 4l): White solid, 83%. ^{1}H NMR (400 MHz, DMSO-d6) δ 9.20 (s, 1H), 7.73 (dt, J = 3.4, 1.8 Hz, 1H), 7.12 (s, 4H), 5.11 (d, J = 3.3 Hz, 1H), 3.97 (q, J = 7.1 Hz, 2H), 2.25 (d, J = 5.0 Hz, 6H), 1.10 (t, J = 7.1 Hz, 3H). ^{13}C NMR (100 MHz, DMSO) δ 165.81, 152.68, 148.64, 142.40, 136.85, 129.36, 126.62, 99.84, 59.65, 54.08, 21.11, 18.24, 14.56. known compound [1].

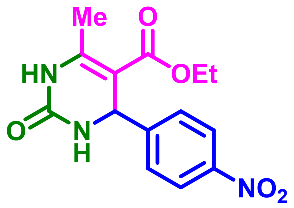

ethyl 4-methyl-6-(4-nitrophenyl)-2-oxo-1,2,3,4-tetrahydropyrimidine-5-carboxylate (Table 2, 4n): Yellow solid, 80%. ^{1}H NMR (400 MHz, DMSO-d6) δ 9.37 (s, 1H), 8.25 – 8.17 (m, 2H), 7.91 (dd, J = 3.5, 2.0 Hz, 1H), 7.54 – 7.47 (m, 2H), 5.27 (d, J = 3.3 Hz, 1H), 3.98 (q, J = 7.1 Hz, 2H), 2.26 (s, 3H), 1.09 (t, J = 7.1 Hz, 3H). ^{13}C NMR (100 MHz, DMSO) δ 165.46, 152.40, 152.16, 149.82, 147.11, 128.07, 124.26, 98.56, 59.82, 54.08, 18.30, 14.47. known compound [4].

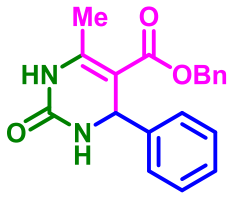

1-(4-methyl-6-phenyl-2-thioxo-1,2,3,4-tetrahydropyrimidin-5-yl)-2-phenylethan-1-one (Table 2, 4o): White solid, 75%. ^{1}H NMR (400 MHz, DMSO-d6) δ 9.32 (s, 1H), 7.81 (q, J = 2.7 Hz, 1H), 7.36 – 7.12 (m, 10H), 5.19 (d, J = 3.1 Hz, 1H), 5.04 (qd, J = 12.8, 2.5 Hz, 2H), 2.28 (d, J = 2.5 Hz, 3H). ^{13}C NMR (100 MHz, DMSO) δ 165.53, 152.47, 149.80, 145.11, 136.96, 128.93, 128.75, 128.17, 128.01, 127.80, 126.79, 99.14, 65.28, 54.39, 18.35. known compound [4]

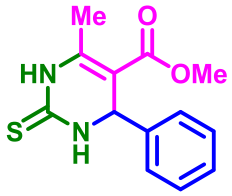

methyl 4-methyl-6-phenyl-2-thioxo-1,2,3,4-tetrahydropyrimidine-5-carboxylate (Table 2, 4o): White solid, 60%. ^{1}H NMR (400 MHz, DMSO-d6) δ 10.40 (s, 1H), 9.74 – 9.68 (m, 1H), 7.35 (t, J = 7.4 Hz, 2H), 7.28 (d, J = 7.1 Hz, 1H), 7.25 – 7.19 (m, 2H), 5.18 (d, J = 3.9 Hz, 1H), 3.55 (s, 3H), 2.30 (s, 3H). ^{13}C NMR (100 MHz, DMSO) δ 166.31, 158.89, 152.62, 148.84, 137.28, 127.79, 114.21, 99.68, 55.51, 53.61, 51.26, 18.28. known compound [5].

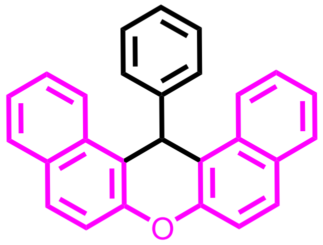

14-phenyl-14H-dibenzo[a,j]xanthenes (5a): White powder. ^{1}H NMR (400 MHz, DMSO-d_6) δ 8.69 (d, J = 8.5 Hz, 2H), 7.93 (dd, J = 8.7, 2.1 Hz, 4H), 7.62 (ddd, J = 8.3, 6.5, 1.4 Hz, 4H), 7.56 (d, J = 8.9 Hz, 2H), 7.50 – 7.41 (m, 2H), 7.14 (t, J = 7.7 Hz, 2H), 6.96 (s, 1H), 6.72 (s, 1H). ^{13}C NMR (100 MHz, DMSO-d_6) δ 147.94, 145.55, 130.87, 130.62, 129.00, 128.59, 128.37, 127.94, 126.93, 126.24, 124.53, 123.42, 117.68, 117.42, 36.47. known compound [6].

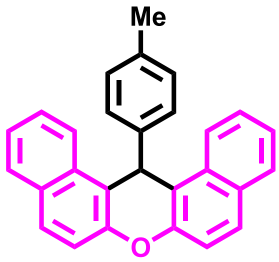

14-(p-tolyl)-14H-dibenzo[a,j]xanthene (5b): White powder.^{1}H NMR (400 MHz, DMSO-d_6) δ 8.66 (d, J = 8.5 Hz, 2H), 8.02 – 7.79 (m, 4H), 7.62 (ddd, J = 8.4, 6.8, 1.4 Hz, 2H), 7.55 (d, J = 8.9 Hz, 2H), 7.52 – 7.45 (m, 2H), 7.45 (ddd, J = 8.0, 6.8, 1.0 Hz, 2H), 6.92 (d, J = 7.9 Hz, 2H), 6.66 (s, 1H). ^{13}C NMR (100 MHz, DMSO-d_6) δ 147.84, 142.65, 135.34, 130.87, 130.62, 128.88, 128.56, 127.81, 126.86, 124.47, 123.45, 117.65, 117.48, 36.10, 20.37. known compound [6].

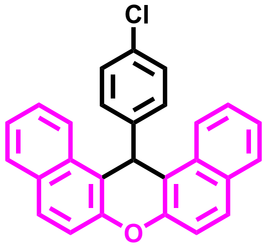

14-(4-chlorophenyl)-14H-dibenzo[a,j]xanthenes (5c): White powder. ^{1}H NMR (400 MHz, DMSO-d_6) δ 8.66 (d, J = 8.5 Hz, 2H), 7.92 (d, J = 8.6 Hz, 4H), 7.63 (t, J = 7.6 Hz, 4H), 7.55 (d, J = 8.9 Hz, 2H), 7.45 (t, J = 7.5 Hz, 2H), 7.19 (d, J = 8.1 Hz, 2H), 6.75 (s, 1H). ^{13}C NMR (100 MHz, DMSO-d_6) δ 147.91, 144.41, 130.89, 130.75, 130.61, 129.64, 129.17, 128.61, 128.33, 127.00,

124.57, 123.26, 117.65, 116.91, 35.81. known compound [6].

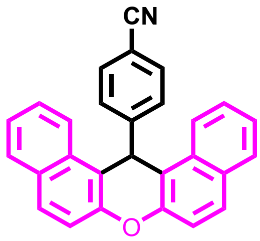

4-(14H-dibenzo[a,j]xanthen-14-yl)benzonitrile (5d): White powder. ^{1}H NMR (400 MHz, DMSO-d_6) δ 8.68 (d, J = 8.5 Hz, 2H), 7.94 (dd, J = 8.6, 5.0 Hz, 4H), 7.83 (d, J = 8.1 Hz, 2H), 7.70 – 7.53 (m, 6H), 7.46 (t, J = 7.5 Hz, 2H), 6.86 (s, 1H). ^{13}C NMR (100 MHz, DMSO-d_6) δ 150.70, 148.01, 132.44, 130.73, 130.62, 129.50, 128.79, 128.69, 127.18, 124.72, 123.18, 118.47, 117.72, 116.34, 109.26, 36.47. known compound [6].

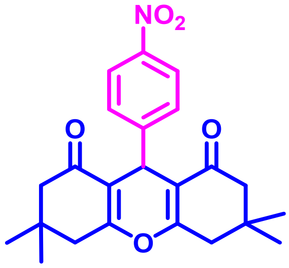

3,3,6,6-tetramethyl-9-(4-nitrophenyl)-3,4,5,6,7,9-hexahydro-1H-xanthene-1,8(2H)-dione (6a): White solid. ^{1}H NMR (400 MHz, $CDCl_3$) δ 8.11 – 8.04 (m, 2H), 7.50 – 7.43 (m, 2H), 4.81 (s, 1H), 2.49 (s, 4H), 2.29 – 2.07 (m, 4H), 1.10 (s, 6H), 0.98 (s, 6H); ^{13}C NMR (100 MHz, $CDCl_3$) δ 196.17, 162.92, 151.49, 146.43, 129.31, 123.33, 114.46, 50.57, 40.79, 32.33, 32.16, 29.16, 27.22. known compound [7] .

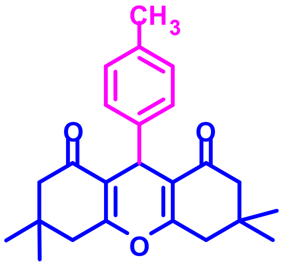

9-(p-tolyl)- 3,3,6,6-tetramethyl-3,4,5,6,7,9-hexahydro-1H-xanthene-1,8(2H)-dione(6b): White solid, ^{1}H NMR (400 MHz, $CDCl_3$) δ 7.17 (d, J = 7.9 Hz, 2H), 7.01 (d, J = 7.8 Hz, 2H), 4.71 (s, 1H),

2.45 (s, 4H), 2.27 – 2.11 (m, 7H), 1.09 (s, 6H), 0.99 (s, 6H); ^{13}C NMR (100 MHz, $CDCl_3$) δ 196.41, 162.06, 141.13, 135.70, 128.72, 128.17, 115.67, 50.70, 40.80, 32.15, 31.37, 29.22, 27.31, 21.02. known compound [7] .

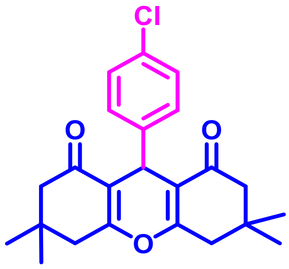

9-(4-chlorophenyl)-3,3,6,6-tetramethyl-3,4,5,6,7,9-hexahydro-1H-xanthene-1,8(2H)-dione(6c): White solid, ^1H NMR (400 MHz, $CDCl_3$) δ 7.23 (d, J = 8.3 Hz, 2H), 7.17 (d, J = 8.3 Hz, 2H), 4.71 (s, 1H), 2.46 (s, 4H), 2.28 – 2.11 (m, 4H), 1.10 (s, 6H), 0.99 (s, 6H); ^{13}C NMR (100 MHz, $CDCl_3$) δ 196.23, 162.37, 142.69, 132.03, 129.75, 128.19, 115.28, 50.71, 40.87, 32.18, 31.47, 29.24, 27.30. known compound [7] .

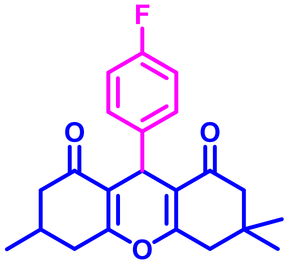

9-(4-fluorophenyl)-3,3,6,6-tetramethyl-3,4,5,6,7,9-hexahydro-1H-xanthene-1,8(2H)-dione (6d): White solid. ^1H NMR (400 MHz, $CDCl_3$) δ 7.24 (dd, J = 8.3, 5.4 Hz, 2H), 6.94 – 6.84 (m, 2H), 4.72 (s, 1H), 2.46 (s, 4H), 2.27 – 2.11 (m, 4H), 1.10 (s, 6H), 0.98 (s, 6H); ^{13}C NMR (100 MHz, CDCl3) δ 196.29, 162.57, 162.28, 160.14, 139.94, 139.91, 129.84, 129.76, 115.50, 114.89, 114.67, 50.72, 40.85, 32.15, 31.20, 29.21, 27.27. known compound [7] .

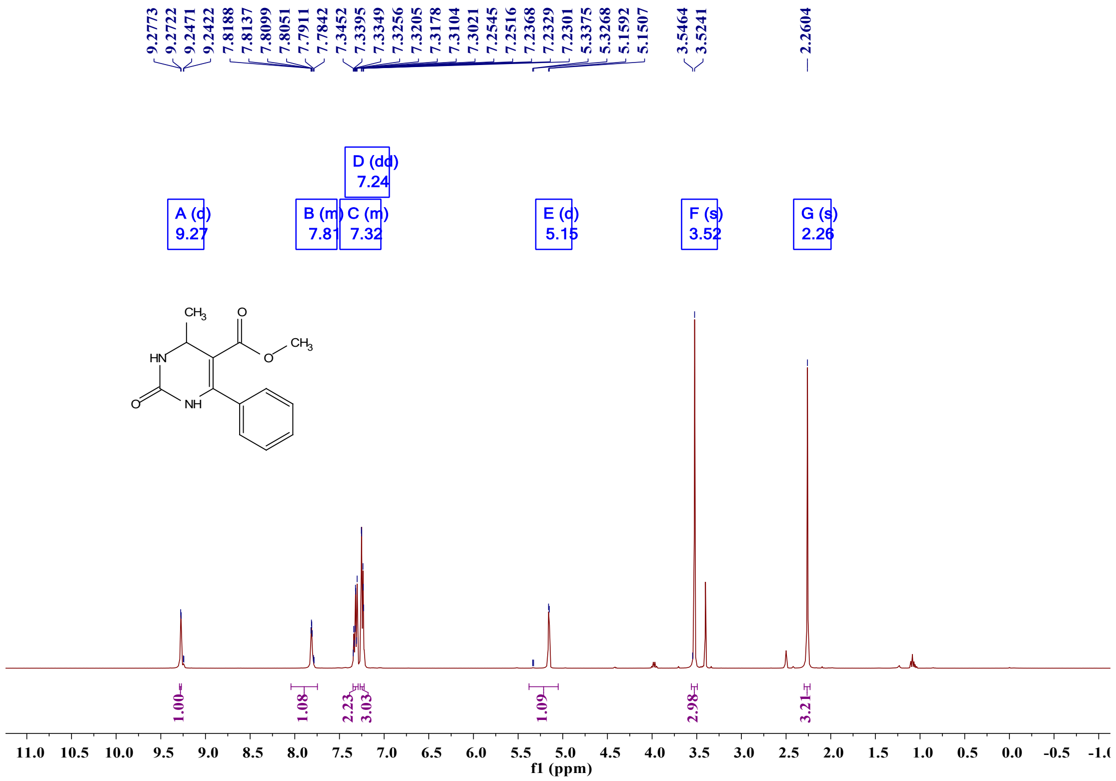
9.2773
9.2722
9.2471
9.2422
7.8188
7.8137
7.8099
7.8051
7.7911
7.7842
7.3452
7.3395
7.3349
7.3256
7.3205
7.3178
7.3104
7.3021
7.2545
7.2516
7.2368
7.2329
7.2301
5.3375
5.3268
5.1592
5.1507
3.5464
3.5241
2.2604
D (dd) 7.24
A (d) 9.27
B (m) 7.81
C (m) 7.32
E (d) 5.15
F (s) 3.52
G (s) 2.26
CH3
O
HN
O
CH3
O
NH
1.00
1.08
2.23
3.03
1.09
2.98
3.21
f1 (ppm)

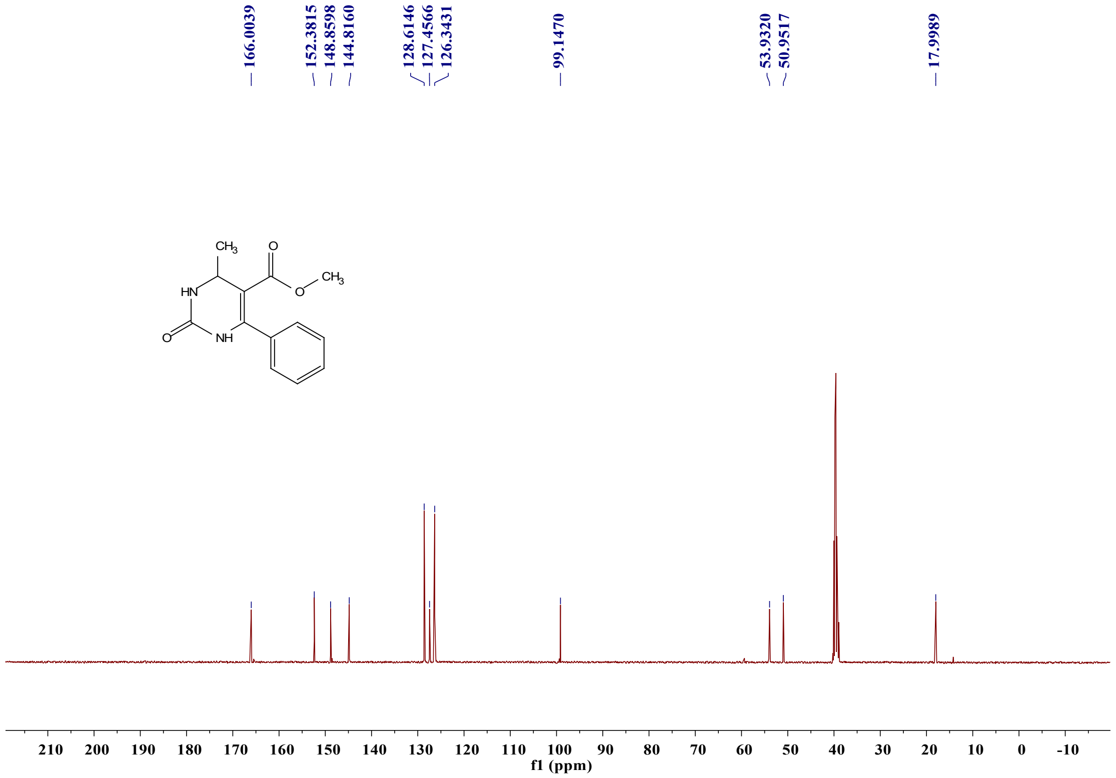
166.0039
152.3815
148.8598
144.8160
128.6146
127.4566
126.3431
99.1470
53.9320
50.9517
17.9989
CH3
O
HN
O
CH3
O
NH
f1 (ppm)

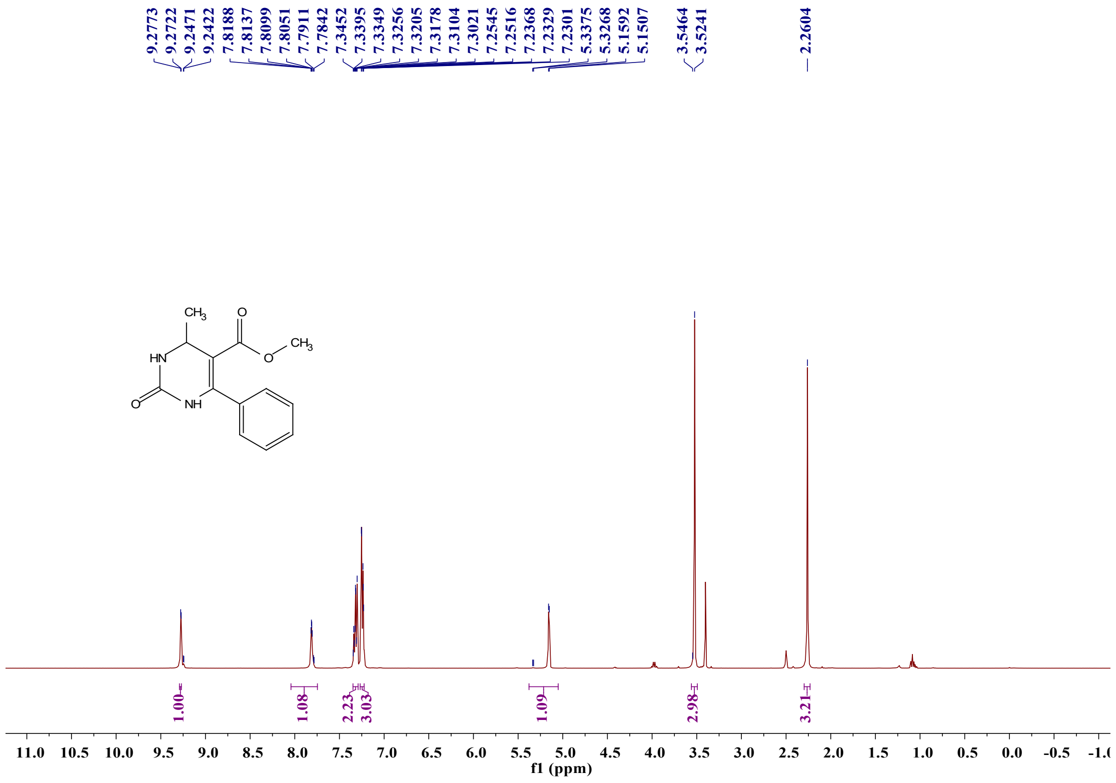
9.2773
9.2722
9.2471
9.2422
7.8188
7.8137
7.8099
7.8051
7.7911
7.7842
7.3452
7.3395
7.3349
7.3256
7.3205
7.3178
7.3104
7.3021
7.2545
7.2516
7.2368
7.2329
7.2301
5.3375
5.3268
5.1592
5.1507
3.5464
3.5241
2.2604
CH3
O
HN
O
CH3
O
NH
1.00
1.08
2.23
3.03
1.09
2.98
3.21
11.0 10.5 10.0 9.5 9.0 8.5 8.0 7.5 7.0 6.5 6.0 5.5 5.0 4.5 4.0 3.5 3.0 2.5 2.0 1.5 1.0 0.5 0.0 -0.5
f1 (ppm)

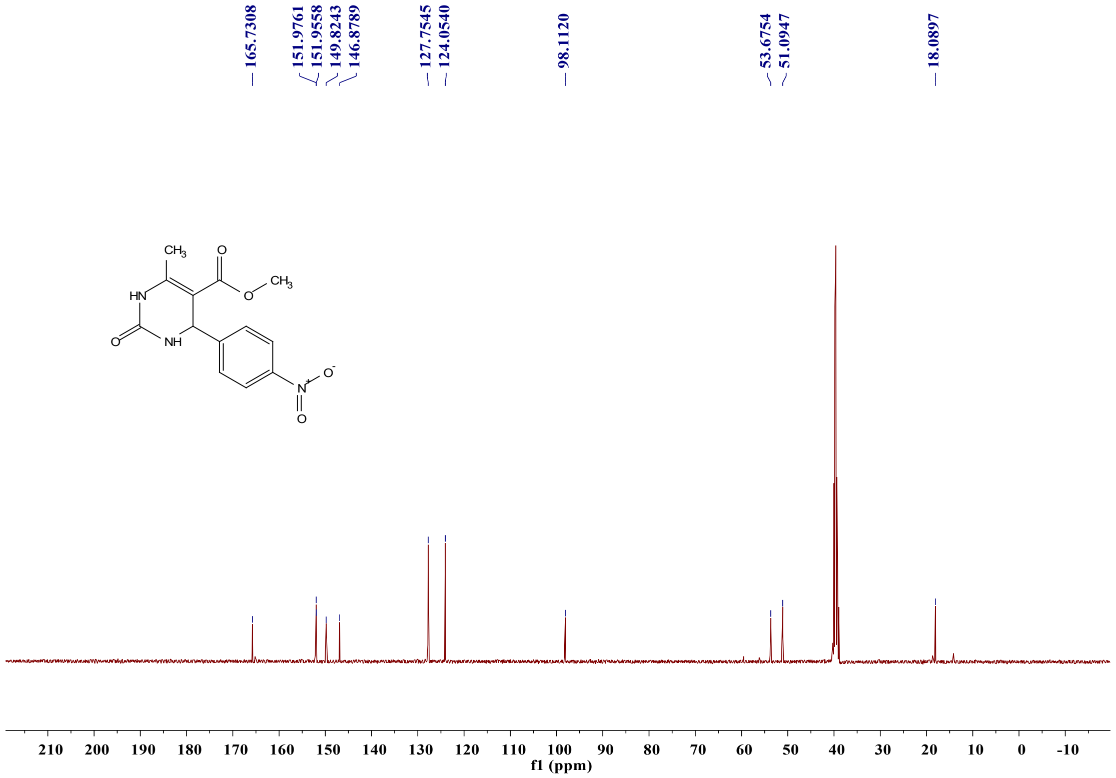
165.7308
151.9761
151.9558
149.8243
146.8789
127.7545
124.0540
98.1120
53.6754
51.0947
18.0897
CH3
O
HN
O
CH3
O
NH
N+
O-
O
210 200 190 180 170 160 150 140 130 120 110 100 90 80 70 60 50 40 30 20 10 0 -10
f1 (ppm)

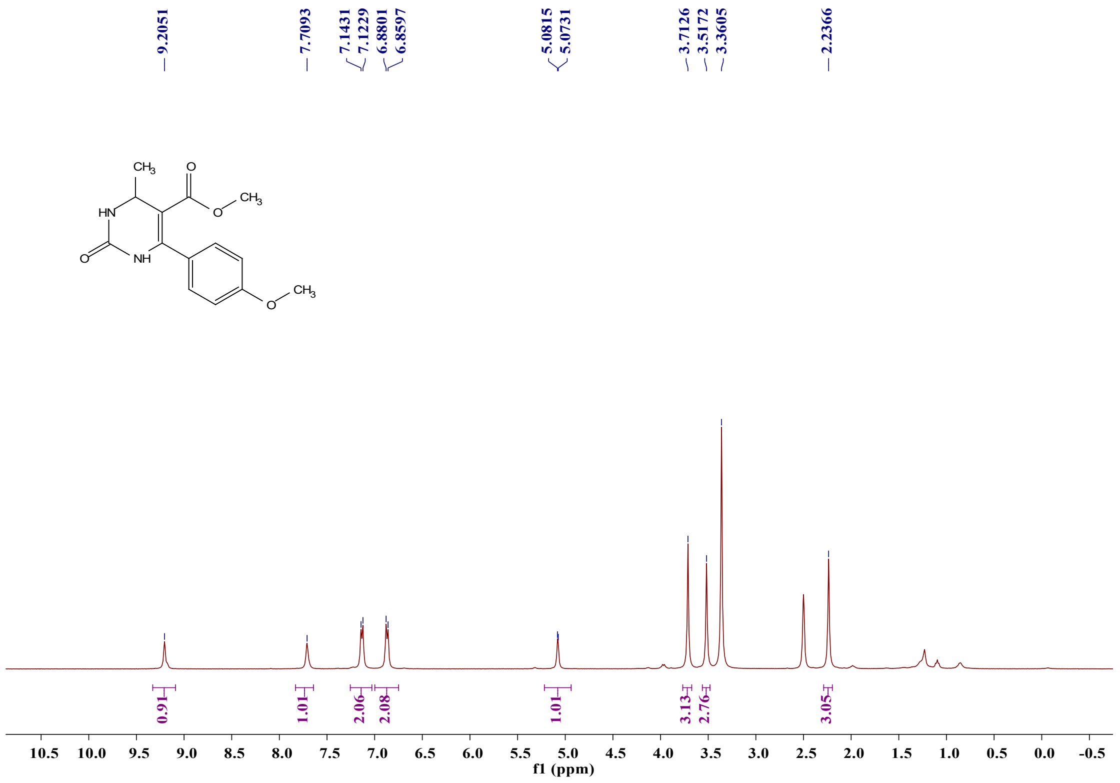
9.2051
7.7093
7.1431
7.1229
6.8801
6.8597
5.0815
5.0731
3.7126
3.5172
3.3605
2.2366
CH3
HN
O
NH
O
CH3
O
CH3
0.91
1.01
2.06
2.08
1.01
3.13
2.76
3.05
f1 (ppm)

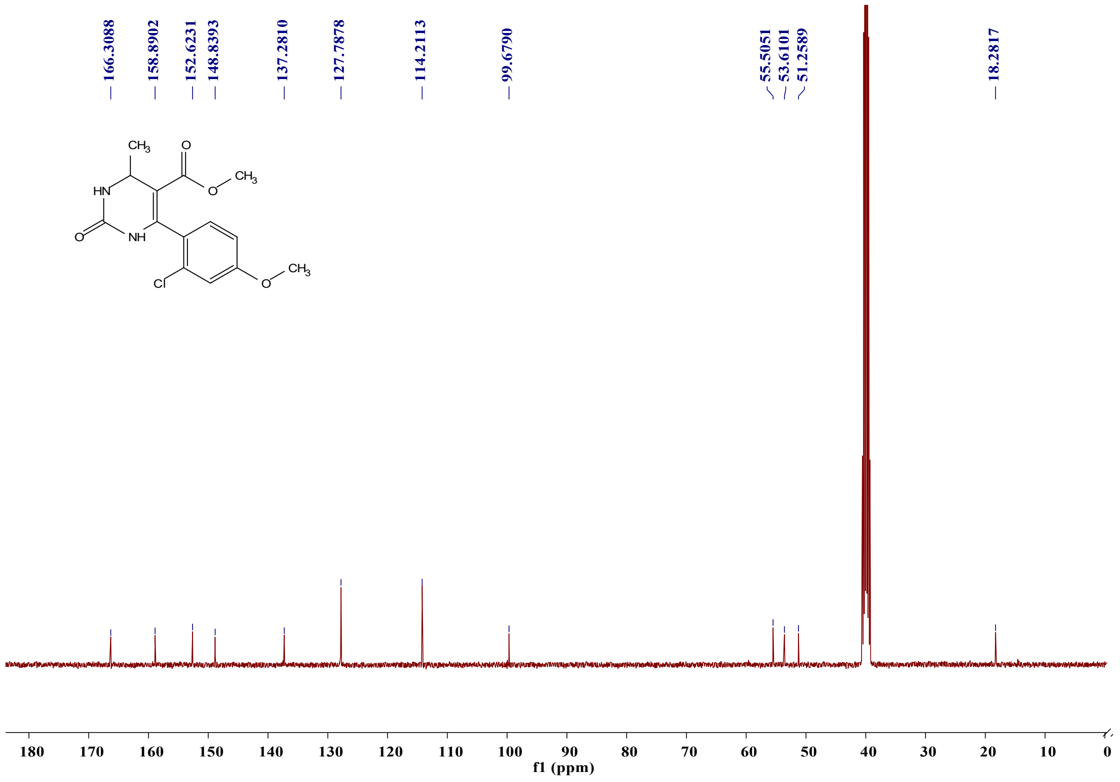
166.3088
158.8902
152.6231
148.8393
137.2810
127.7878
114.2113
99.6790
55.5051
53.6101
51.2589
18.2817
CH3
HN
O
NH
O
CH3
Cl
O
CH3
f1 (ppm)

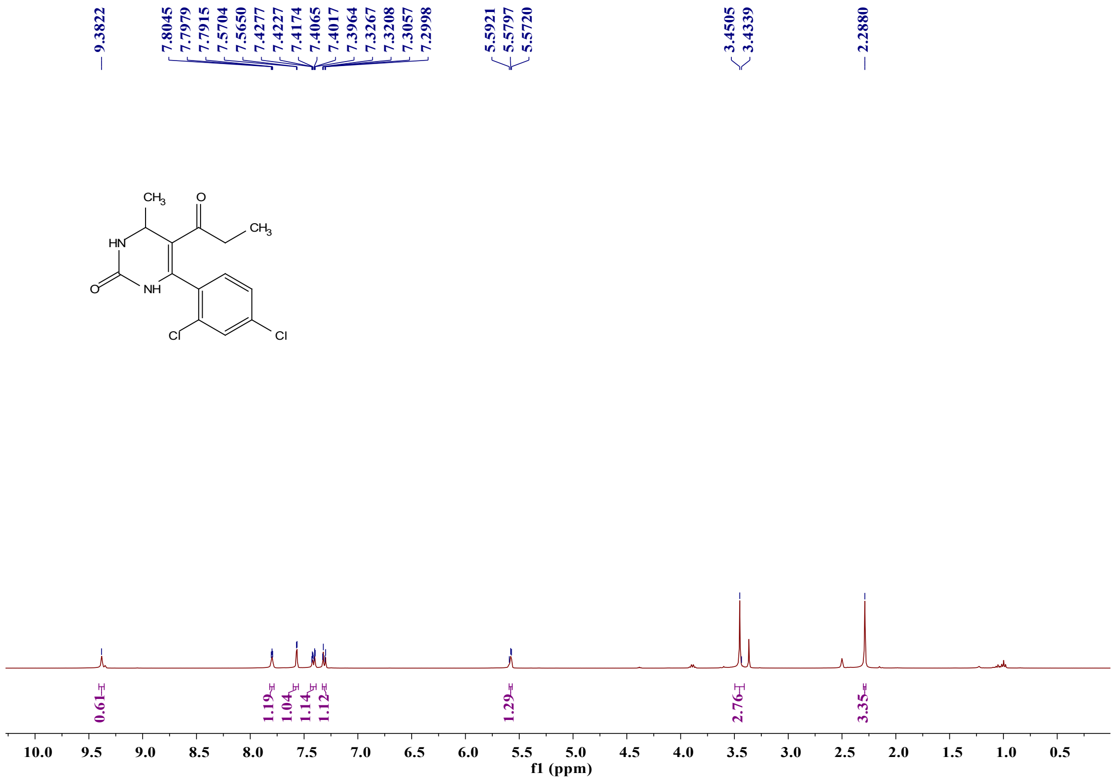
9.3822
7.8045
7.7979
7.7915
7.5704
7.5650
7.4277
7.4227
7.4174
7.4065
7.4017
7.3964
7.3267
7.3208
7.3057
7.2998
5.5921
5.5797
5.5720
3.4505
3.4339
2.2880
CH3
O
CH3
HN
O
NH
Cl
Cl
0.61
1.19
1.04
1.14
1.12
1.29
2.76
3.35
10.0 9.5 9.0 8.5 8.0 7.5 7.0 6.5 6.0 5.5 5.0 4.5 4.0 3.5 3.0 2.5 2.0 1.5 1.0 0.5
f1 (ppm)

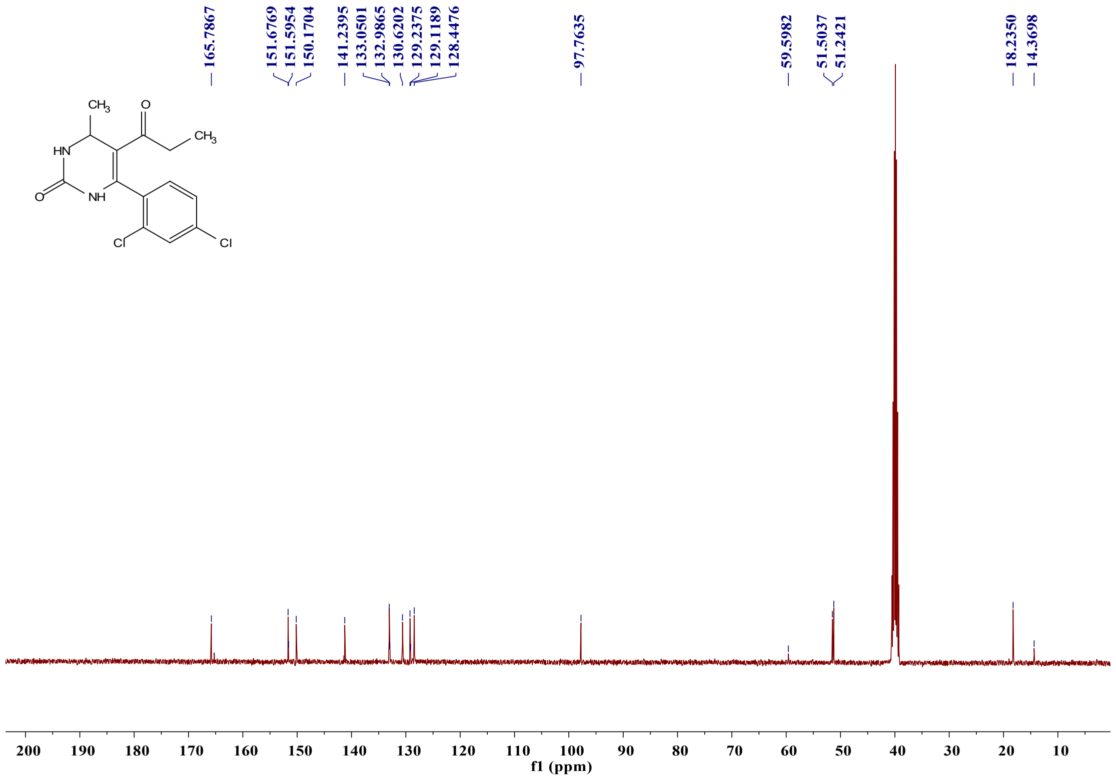
165.7867
151.6769
151.5954
150.1704
141.2395
133.0501
132.9865
130.6202
129.2375
129.1189
128.4476
97.7635
59.5982
51.5037
51.2421
18.2350
14.3698
CH3
O
CH3
HN
O
NH
Cl
Cl
200 190 180 170 160 150 140 130 120 110 100 90 80 70 60 50 40 30 20 10
f1 (ppm)

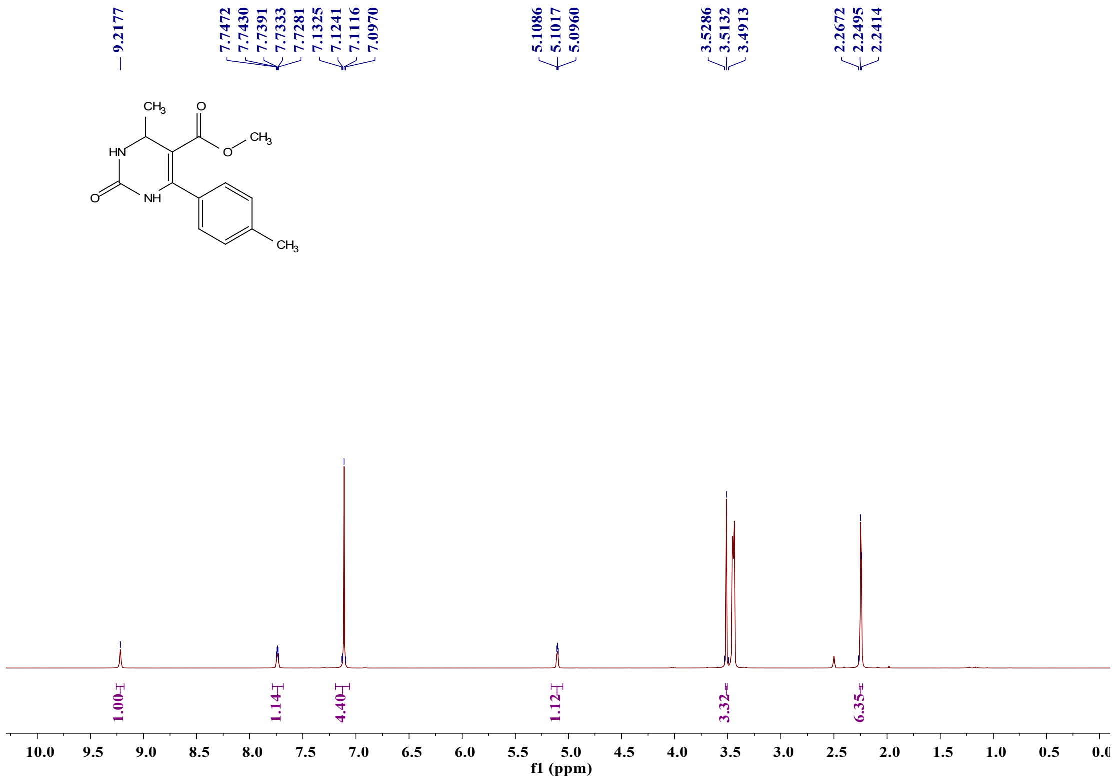
9.2177
7.7472
7.7430
7.7391
7.7333
7.7281
7.1325
7.1241
7.1116
7.0970
5.1086
5.1017
5.0960
3.5286
3.5132
3.4913
2.2672
2.2495
2.2414
CH3
O
CH3
HN
O
O
NH
CH3
1.00
1.14
4.40
1.12
3.32
6.35
10.0 9.5 9.0 8.5 8.0 7.5 7.0 6.5 6.0 5.5 5.0 4.5 4.0 3.5 3.0 2.5 2.0 1.5 1.0 0.5 0.0
f1 (ppm)

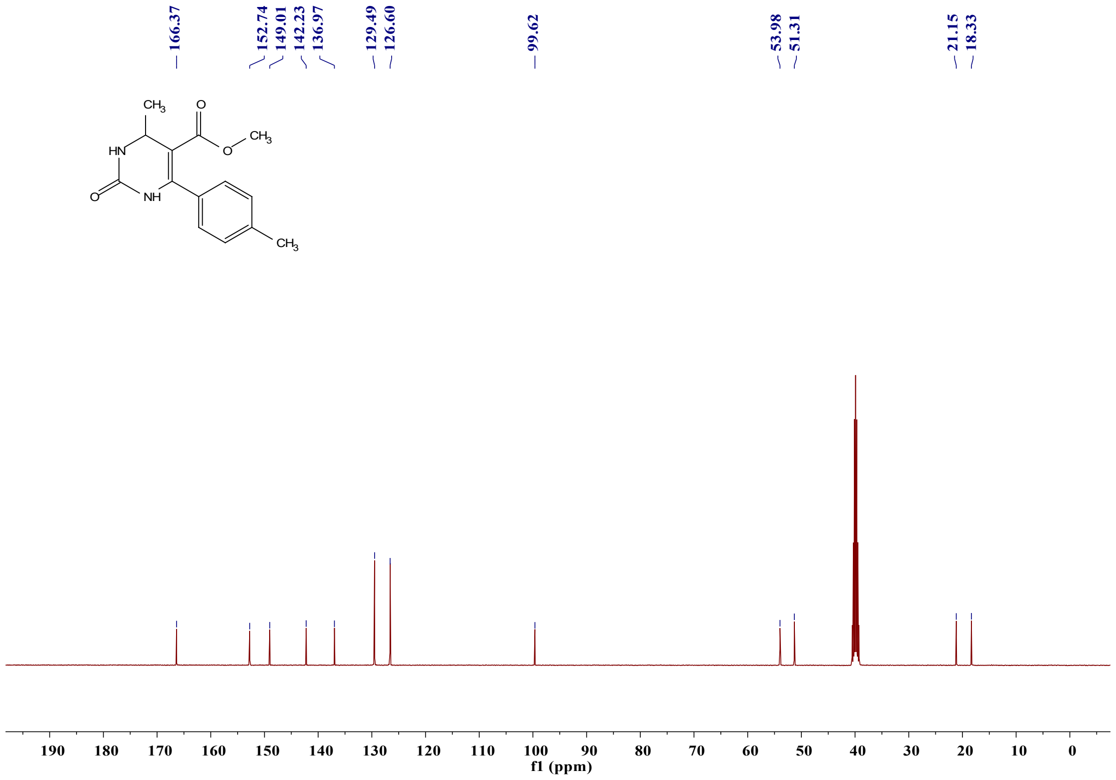
166.37
152.74
149.01
142.23
136.97
129.49
126.60
99.62
53.98
51.31
21.15
18.33
CH3
O
CH3
HN
O
O
NH
CH3
190 180 170 160 150 140 130 120 110 100 90 80 70 60 50 40 30 20 10 0
f1 (ppm)

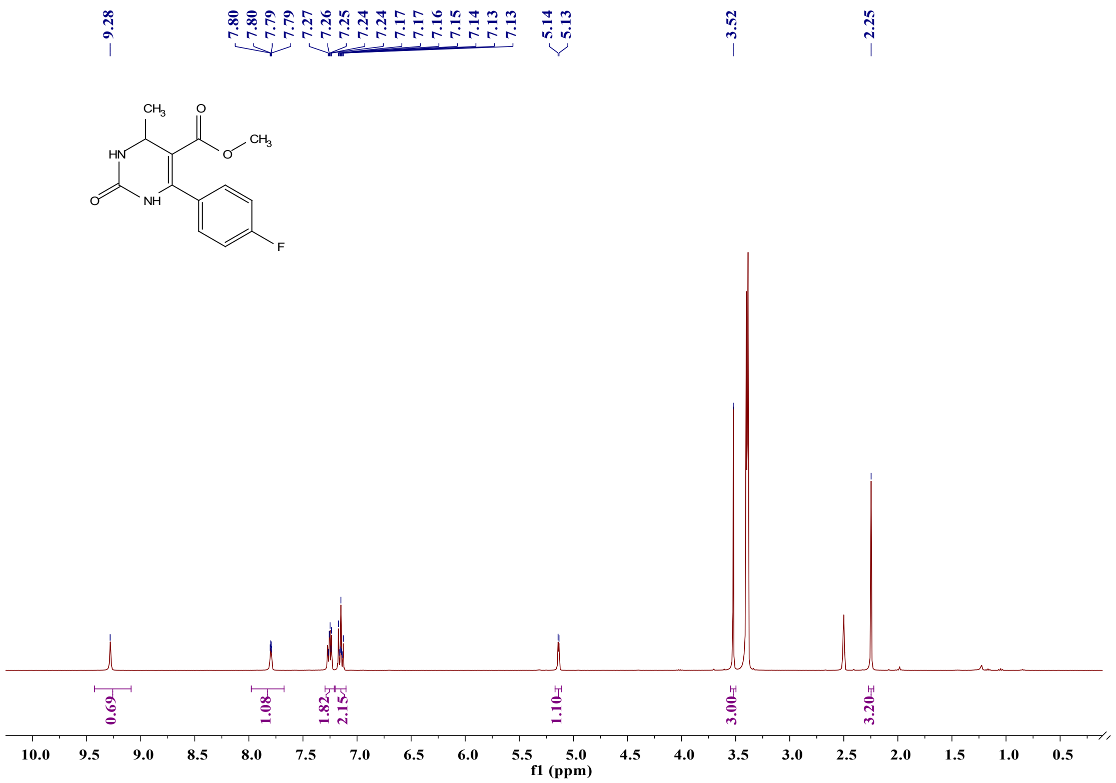
9.28
7.80
7.80
7.79
7.79
7.27
7.26
7.25
7.24
7.24
7.17
7.17
7.16
7.15
7.14
7.13
7.13
5.14
5.13
3.52
2.25
CH3
O
CH3
HN
O
O
NH
F
0.69
1.08
1.82
2.15
1.10
3.00
3.20
10.0 9.5 9.0 8.5 8.0 7.5 7.0 6.5 6.0 5.5 5.0 4.5 4.0 3.5 3.0 2.5 2.0 1.5 1.0 0.5
f1 (ppm)

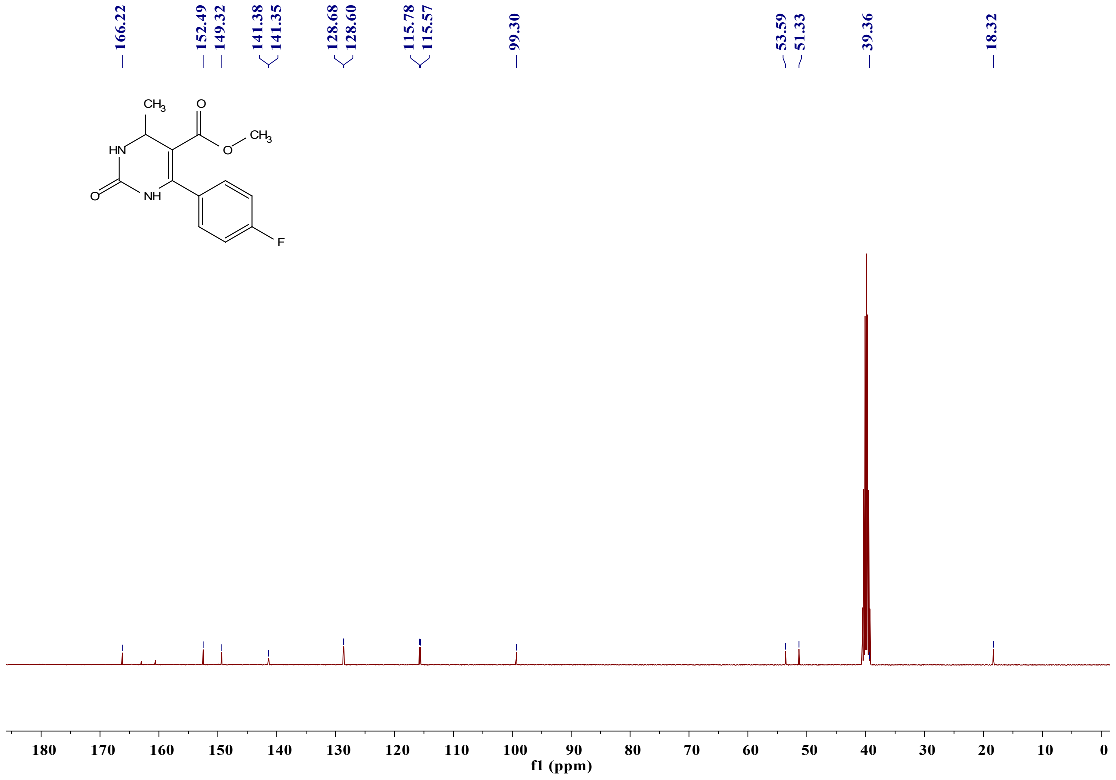
166.22
152.49
149.32
141.38
141.35
128.68
128.60
115.78
115.57
99.30
53.59
51.33
39.36
18.32
CH3
O
CH3
HN
O
O
NH
F
180 170 160 150 140 130 120 110 100 90 80 70 60 50 40 30 20 10 0
f1 (ppm)

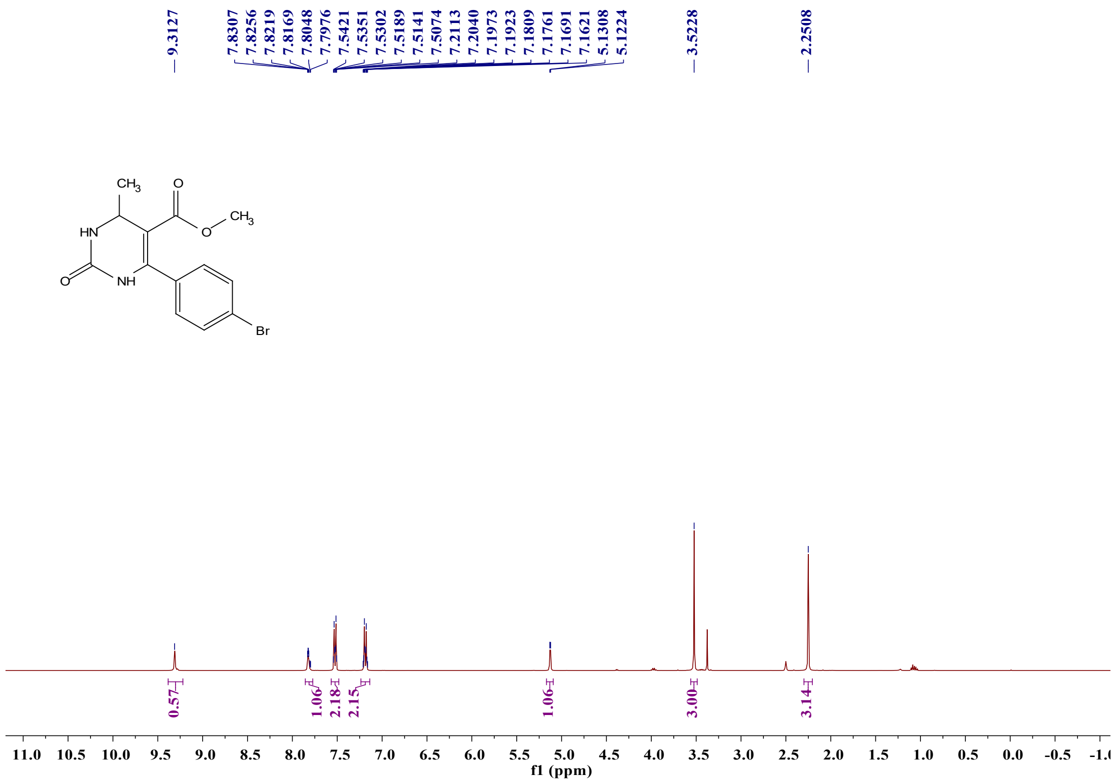
9.3127
7.8307
7.8256
7.8219
7.8169
7.8048
7.7976
7.5421
7.5351
7.5302
7.5189
7.5141
7.5074
7.2113
7.2040
7.1973
7.1923
7.1809
7.1761
7.1691
7.1621
5.1308
5.1224
3.5228
2.2508
CH3
O
HN
O
CH3
O
NH
Br
0.57
1.06
2.18
2.15
1.06
3.00
3.14
11.0 10.5 10.0 9.5 9.0 8.5 8.0 7.5 7.0 6.5 6.0 5.5 5.0 4.5 4.0 3.5 3.0 2.5 2.0 1.5 1.0 0.5 0.0 -0.5
f1 (ppm)

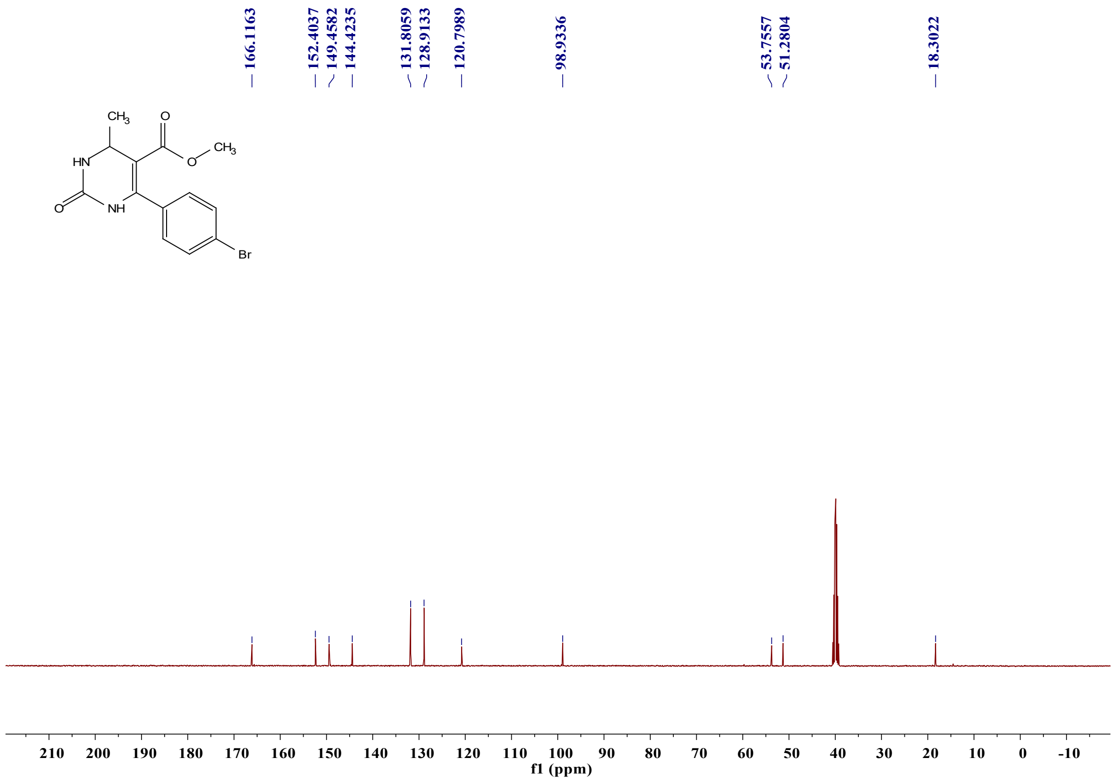
166.1163
152.4037
149.4582
144.4235
131.8059
128.9133
120.7989
98.9336
53.7557
51.2804
18.3022
CH3
O
HN
O
CH3
O
NH
Br
210 200 190 180 170 160 150 140 130 120 110 100 90 80 70 60 50 40 30 20 10 0 -10
f1 (ppm)

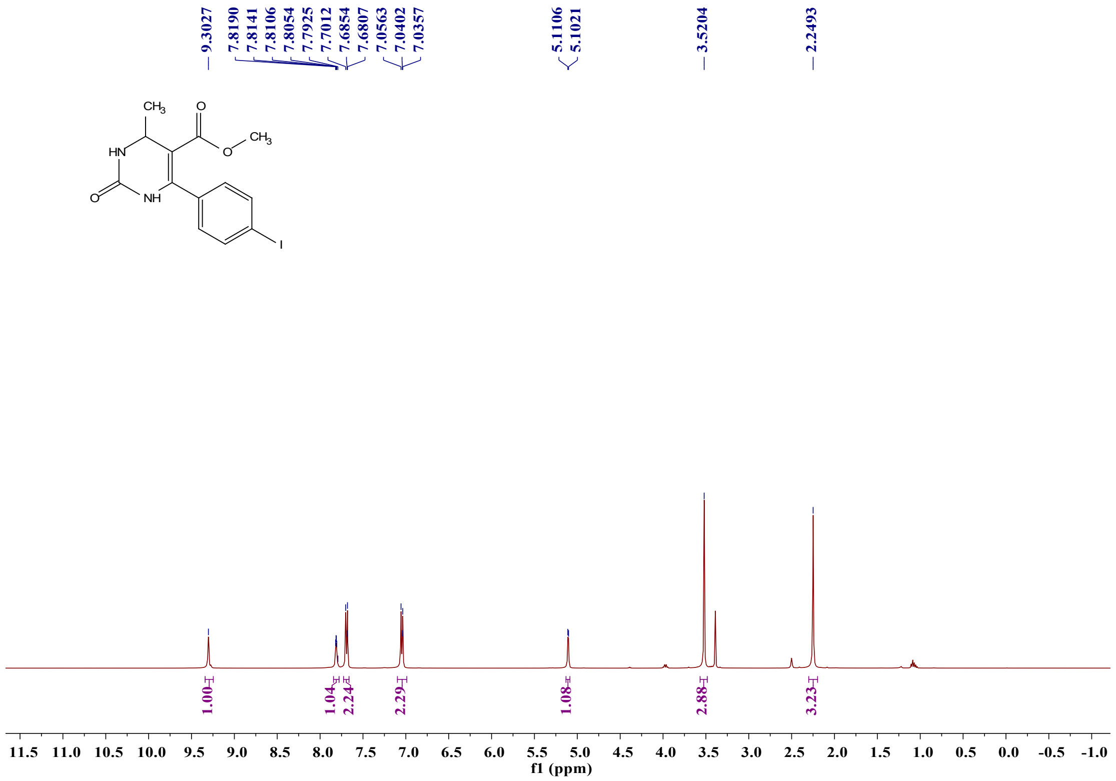
9.3027
7.8190
7.8141
7.8106
7.8054
7.7925
7.7012
7.6854
7.6807
7.0563
7.0402
7.0357
5.1106
5.1021
3.5204
2.2493
1.00
1.04
2.24
2.29
1.08
2.88
3.23
11.5 11.0 10.5 10.0 9.5 9.0 8.5 8.0 7.5 7.0 6.5 6.0 5.5 5.0 4.5 4.0 3.5 3.0 2.5 2.0 1.5 1.0 0.5 0.0 -0.5 -1.0
f1 (ppm)

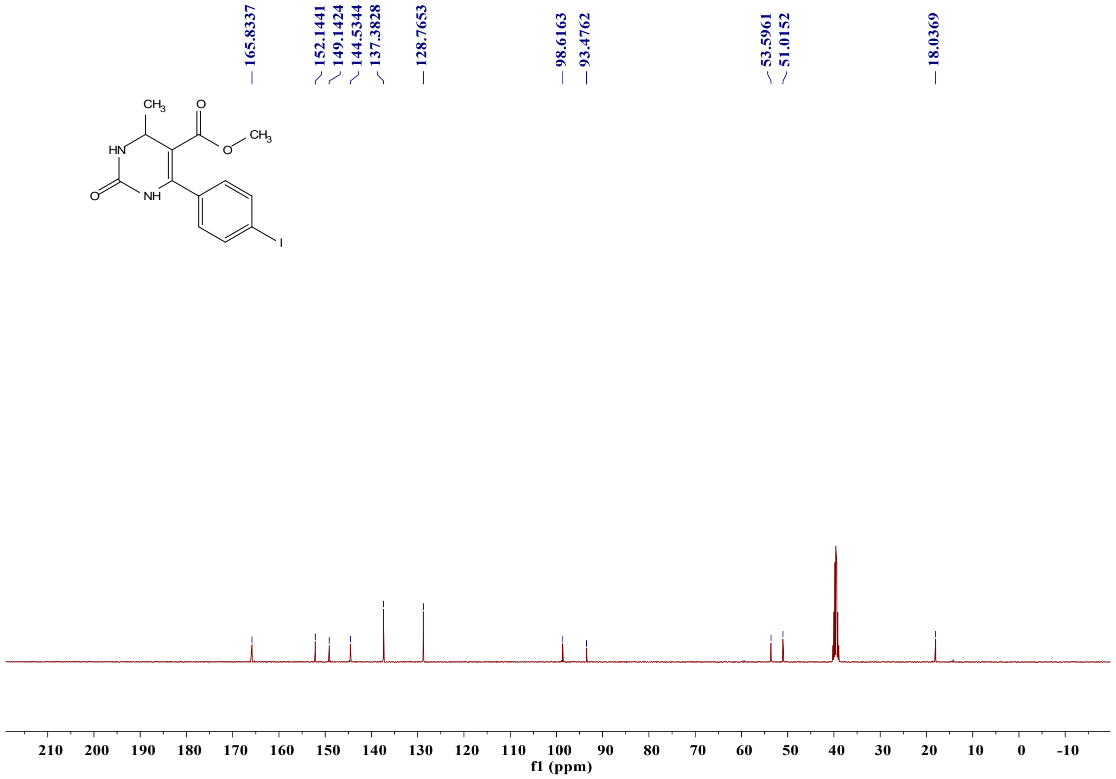
165.8337
152.1441
149.1424
144.5344
137.3828
128.7653
98.6163
93.4762
53.5961
51.0152
18.0369
210 200 190 180 170 160 150 140 130 120 110 100 90 80 70 60 50 40 30 20 10 0 -10
f1 (ppm)

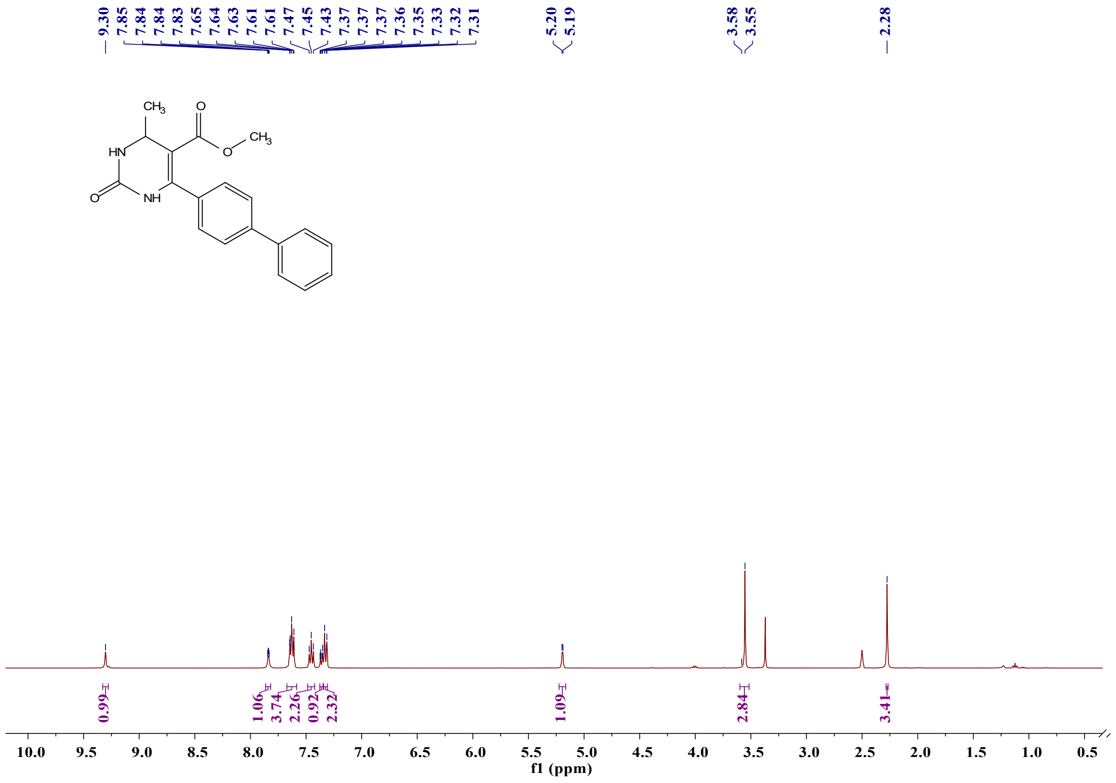
9.30
7.85
7.84
7.84
7.83
7.65
7.64
7.63
7.61
7.61
7.47
7.45
7.43
7.37
7.37
7.37
7.36
7.35
7.33
7.32
7.31
5.20
5.19
3.58
3.55
2.28
CH3
O
O
CH3
HN
O
NH
0.99
1.06
3.74
2.26
0.92
2.32
1.09
2.84
3.41
10.0 9.5 9.0 8.5 8.0 7.5 7.0 6.5 6.0 5.5 5.0 4.5 4.0 3.5 3.0 2.5 2.0 1.5 1.0 0.5
f1 (ppm)

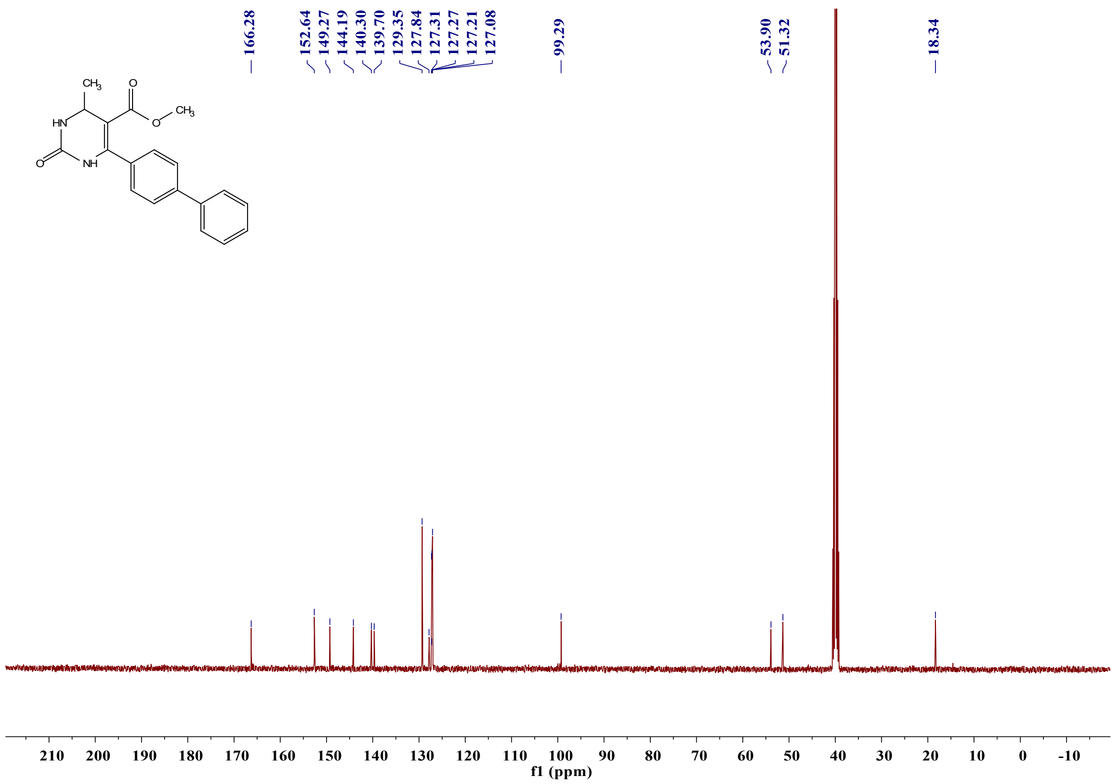
166.28
152.64
149.27
144.19
140.30
139.70
129.35
127.84
127.31
127.27
127.21
127.08
99.29
53.90
51.32
18.34
CH3
O
O
CH3
HN
O
NH
210 200 190 180 170 160 150 140 130 120 110 100 90 80 70 60 50 40 30 20 10 0 -10
f1 (ppm)

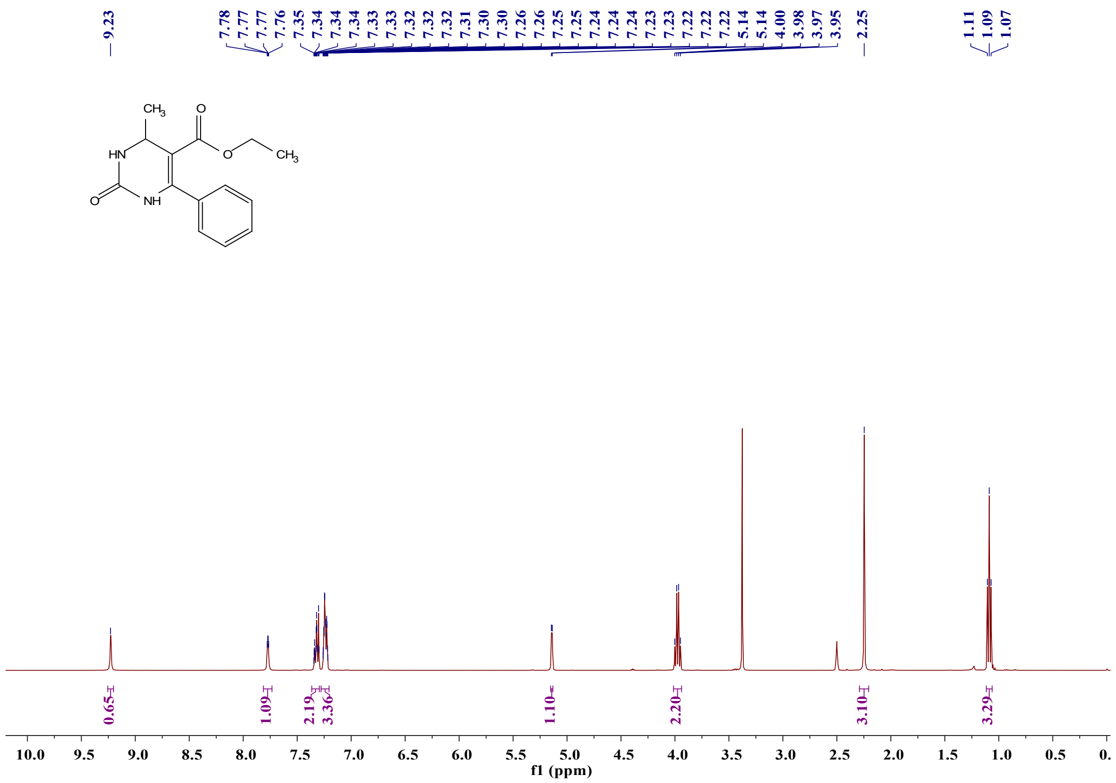
9.23
7.78 7.77 7.77 7.76 7.35 7.34 7.34 7.34 7.33 7.33 7.32 7.32 7.32 7.31 7.30 7.30 7.26 7.26 7.25 7.25 7.24 7.24 7.24 7.23 7.23 7.22 7.22 7.22 7.22 5.14 5.14 4.00 3.98 3.97 3.95
2.25
1.11 1.09 1.07
CH_3
O
HN
O
CH_3
O
NH
0.65
1.09
2.19
3.36
1.10
2.20
3.10
3.29
10.0 9.5 9.0 8.5 8.0 7.5 7.0 6.5 6.0 5.5 5.0 4.5 4.0 3.5 3.0 2.5 2.0 1.5 1.0 0.5 0.
f1 (ppm)

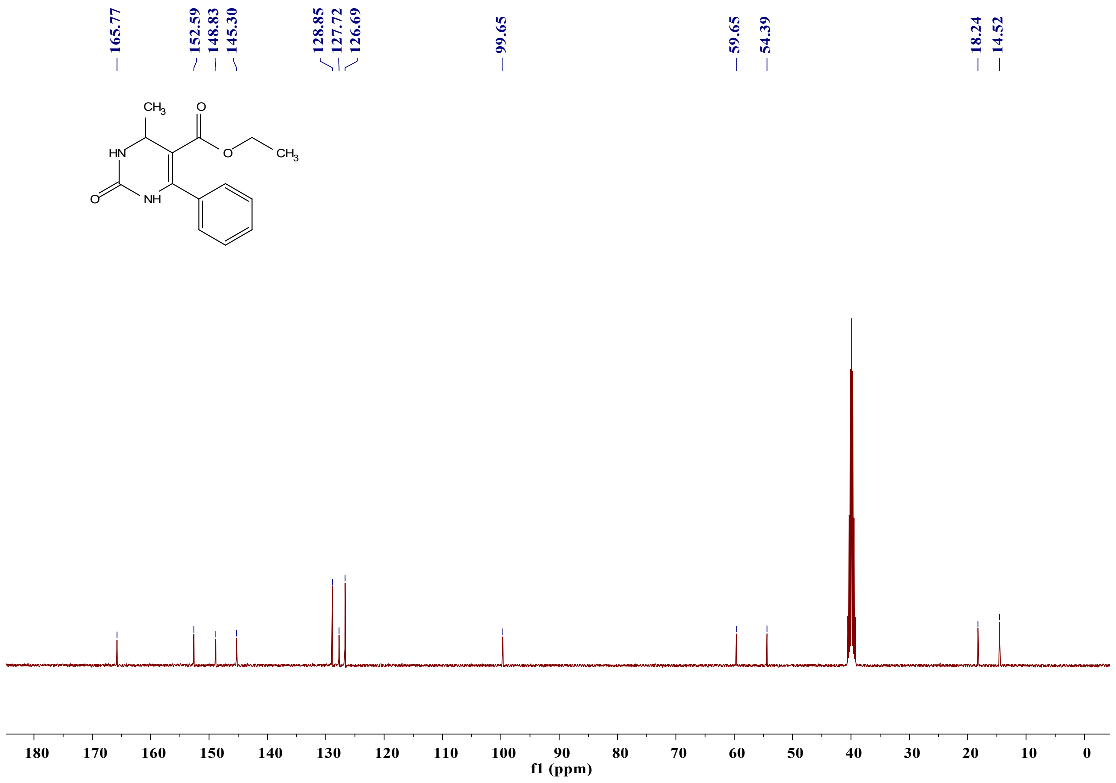
165.77
152.59 148.83 145.30
128.85 127.72 126.69
99.65
59.65 54.39
18.24 14.52
CH_3
O
HN
O
CH_3
O
NH
180 170 160 150 140 130 120 110 100 90 80 70 60 50 40 30 20 10 0
f1 (ppm)

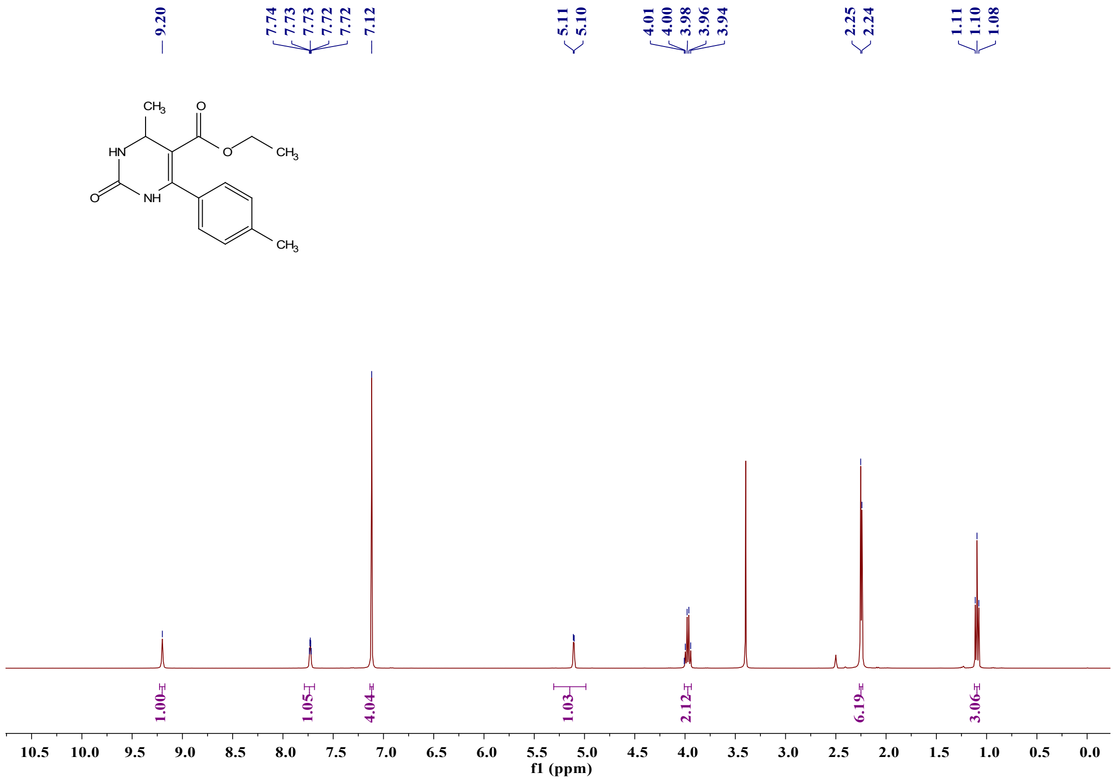
9.20
7.74
7.73
7.73
7.72
7.72
7.12
5.11
5.10
4.01
4.00
3.98
3.96
3.94
2.25
2.24
1.11
1.10
1.08
1.00
1.05
4.04
1.03
2.12
6.19
3.06
10.5 10.0 9.5 9.0 8.5 8.0 7.5 7.0 6.5 6.0 5.5 5.0 4.5 4.0 3.5 3.0 2.5 2.0 1.5 1.0 0.5 0.0
f1 (ppm)

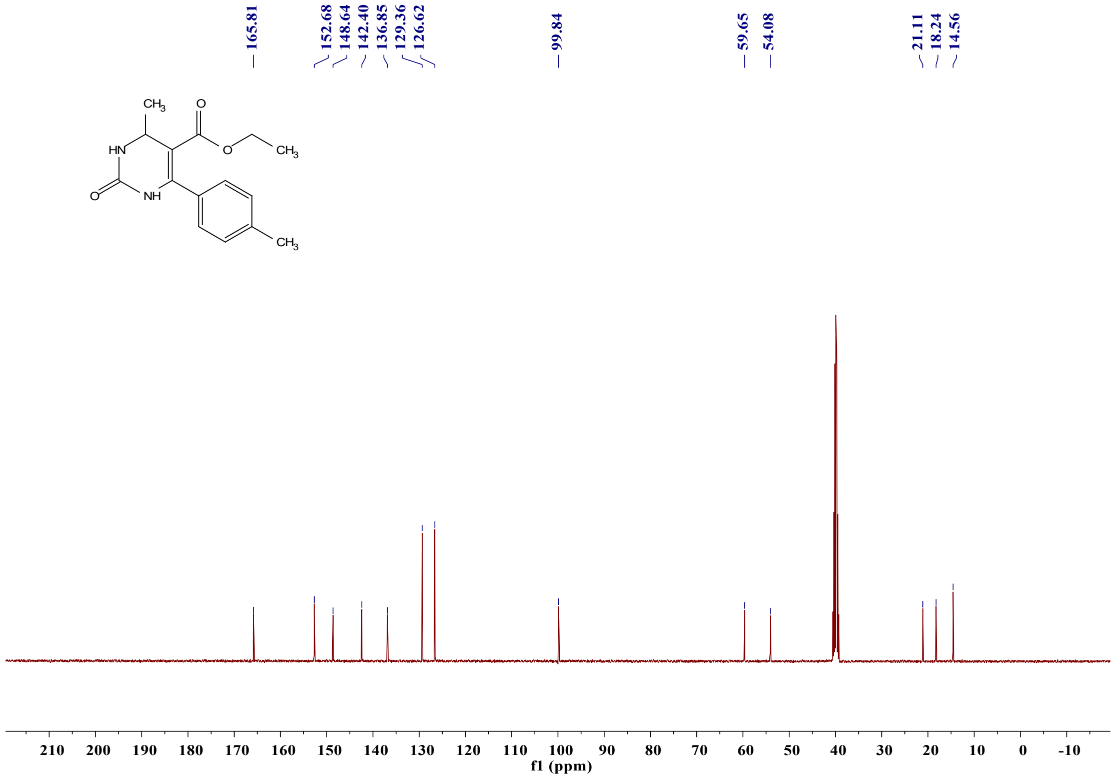
165.81
152.68
148.64
142.40
136.85
129.36
126.62
99.84
59.65
54.08
21.11
18.24
14.56
210 200 190 180 170 160 150 140 130 120 110 100 90 80 70 60 50 40 30 20 10 0 -10
f1 (ppm)

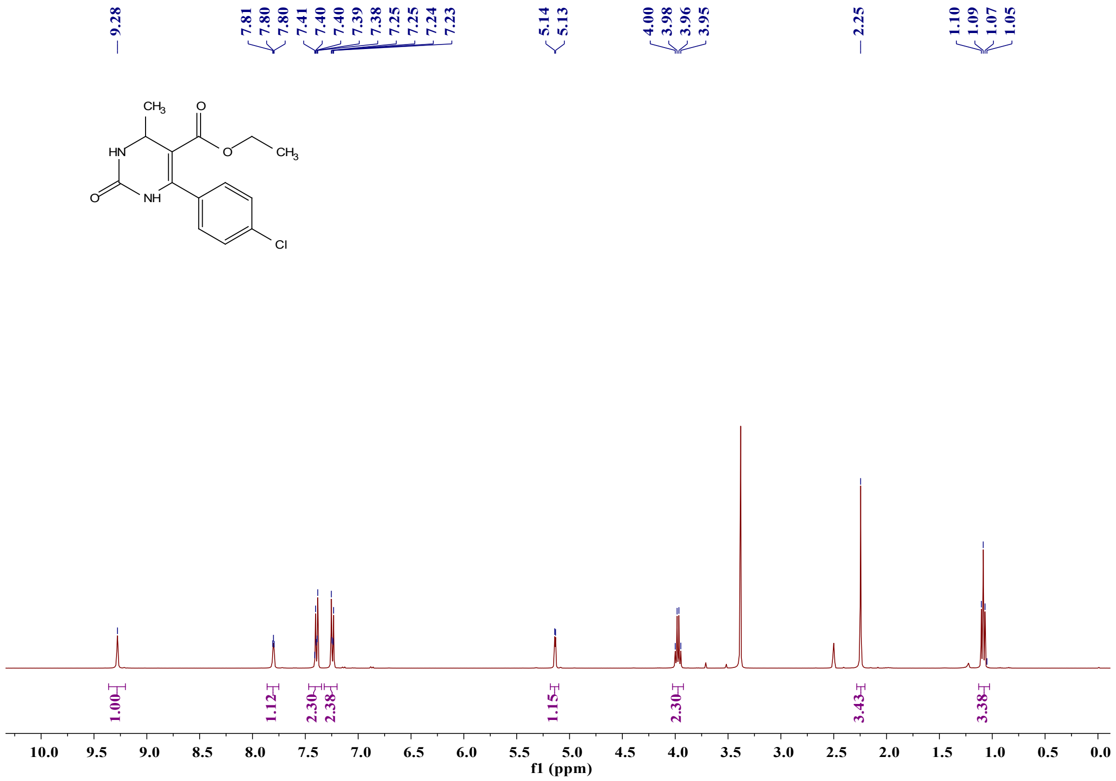
9.28
7.81 7.80 7.80 7.41 7.40 7.40 7.39 7.38 7.25 7.25 7.24 7.23
5.14 5.13
4.00 3.98 3.96 3.95
2.25
1.10 1.09 1.07 1.05
CH3
O
HN
O
CH3
O
NH
Cl
1.00
1.12
2.30
2.38
1.15
2.30
3.43
3.38
10.0 9.5 9.0 8.5 8.0 7.5 7.0 6.5 6.0 5.5 5.0 4.5 4.0 3.5 3.0 2.5 2.0 1.5 1.0 0.5 0.0
f1 (ppm)

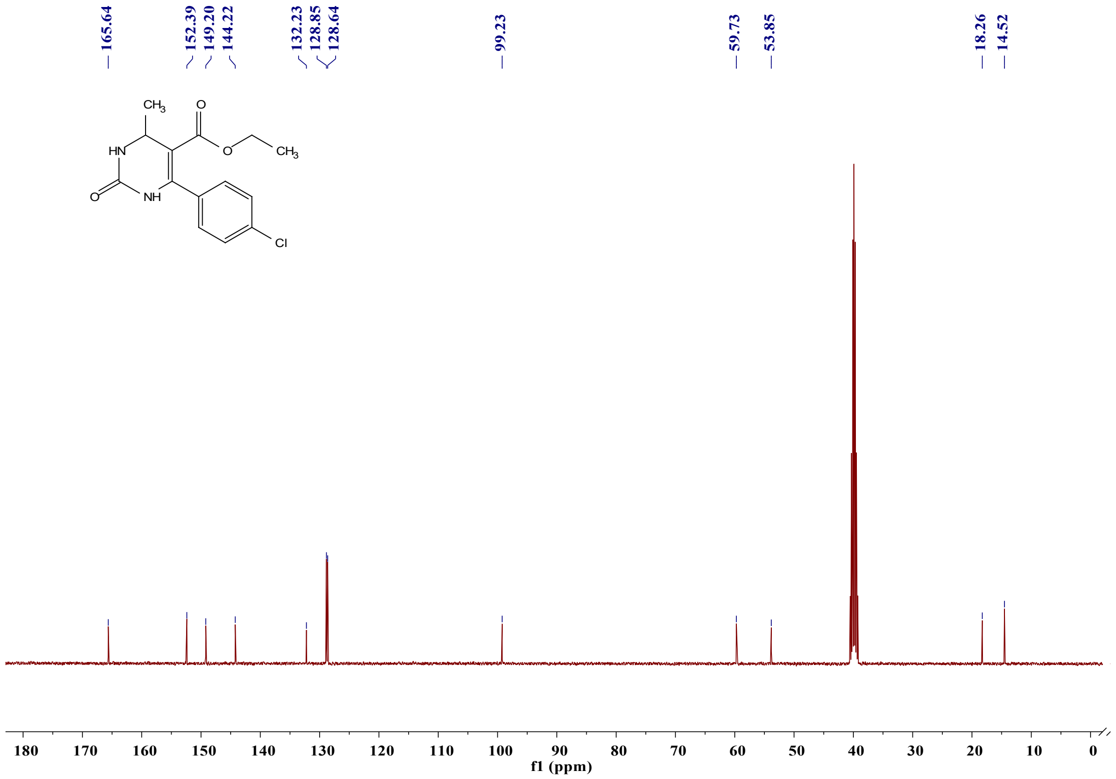
165.64
152.39 149.20 144.22
132.23 128.85 128.64
99.23
59.73
53.85
18.26
14.52
CH3
O
HN
O
CH3
O
NH
Cl
180 170 160 150 140 130 120 110 100 90 80 70 60 50 40 30 20 10 0
f1 (ppm)

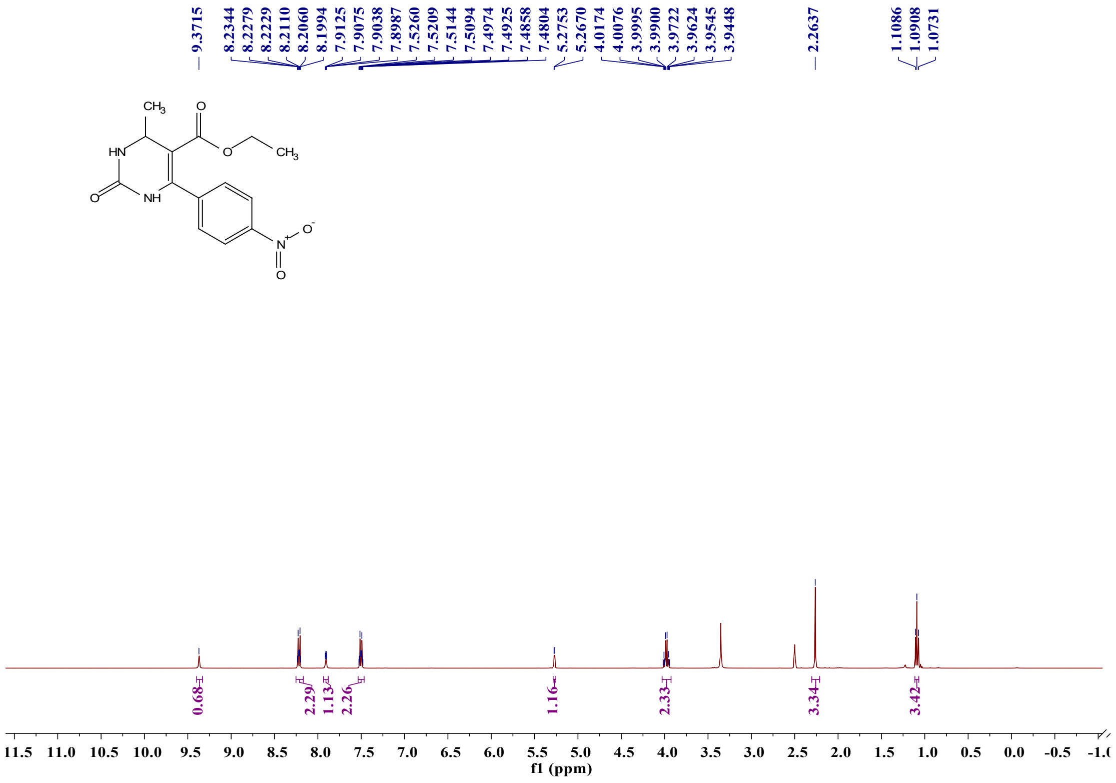
9.3715
8.2344
8.2279
8.2229
8.2110
8.2060
8.1994
7.9125
7.9075
7.9038
7.8987
7.5260
7.5209
7.5144
7.5094
7.4974
7.4925
7.4858
7.4804
5.2753
5.2670
4.0174
4.0076
3.9995
3.9900
3.9722
3.9624
3.9545
3.9448
2.2637
1.1086
1.0908
1.0731
0.68
2.29
1.13
2.26
1.16
2.33
3.34
3.42
f1 (ppm)

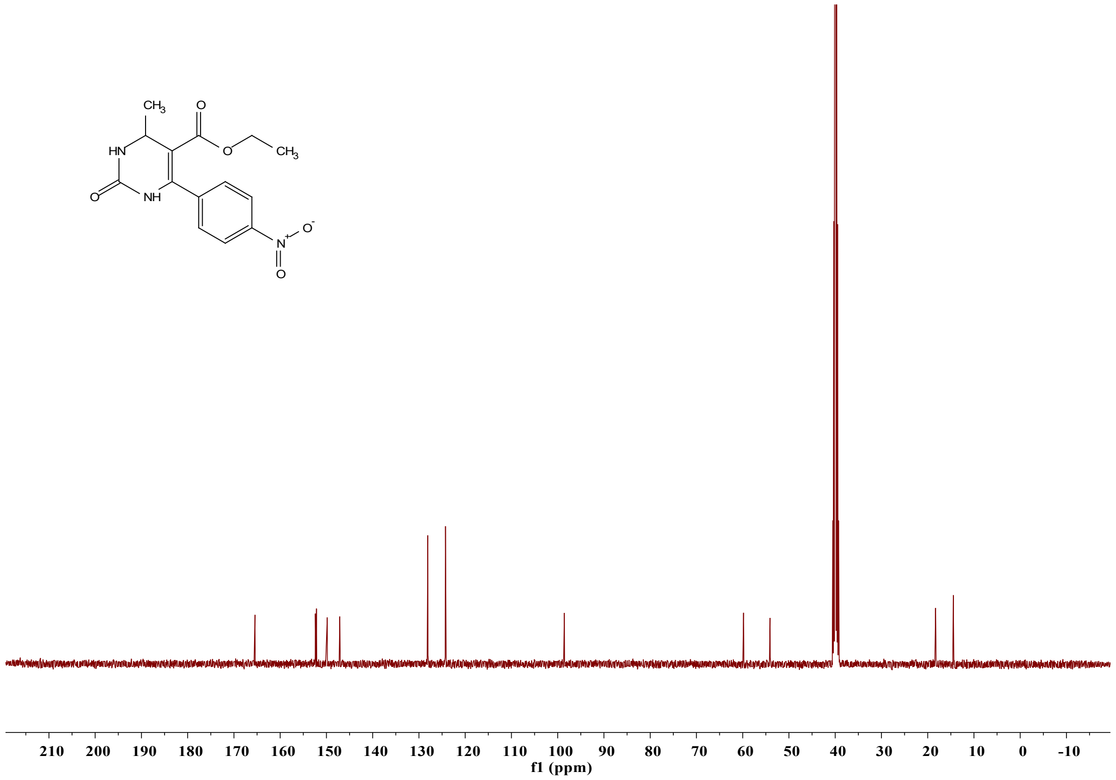
f1 (ppm)

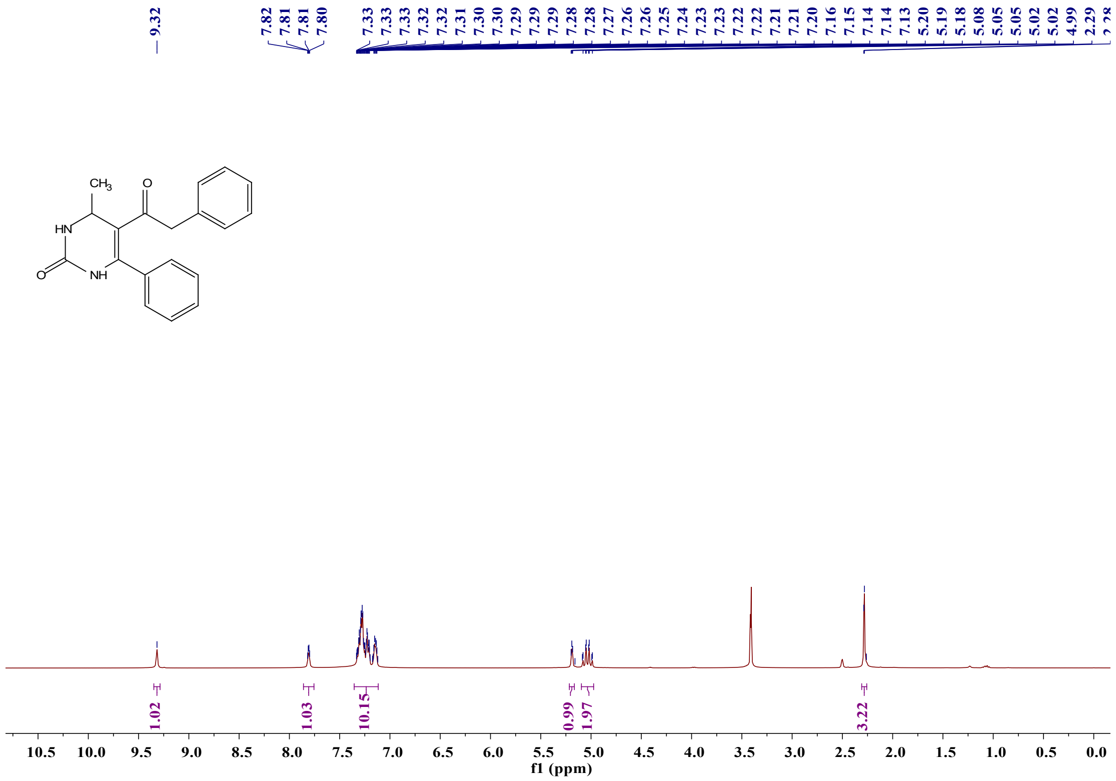
9.32
7.82 7.81 7.81 7.80
7.33 7.33 7.33 7.32 7.32 7.31 7.30 7.29 7.29 7.29 7.28 7.28 7.27 7.26 7.26 7.25 7.24 7.23 7.23 7.22 7.22 7.21 7.21 7.20 7.16 7.15 7.14 7.14 7.13
5.20 5.19 5.18 5.08 5.05 5.05 5.02 5.02 4.99 2.29
CH3
O
HN
NH
O
1.02
1.03
10.15
0.99
1.97
3.22
10.5 10.0 9.5 9.0 8.5 8.0 7.5 7.0 6.5 6.0 5.5 5.0 4.5 4.0 3.5 3.0 2.5 2.0 1.5 1.0 0.5 0.0
f1 (ppm)

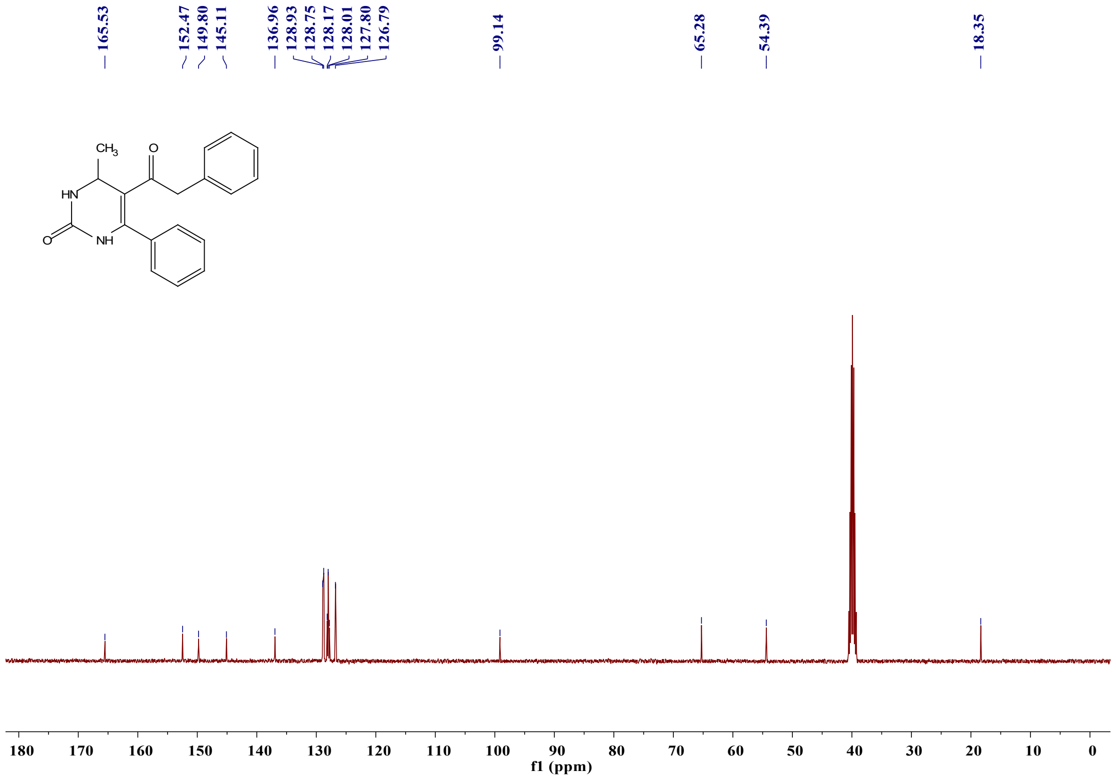
165.53
152.47 149.80 145.11
136.96 128.93 128.75 128.17 128.01 127.80 126.79
99.14
65.28
54.39
18.35
CH3
O
HN
NH
O
180 170 160 150 140 130 120 110 100 90 80 70 60 50 40 30 20 10 0
f1 (ppm)

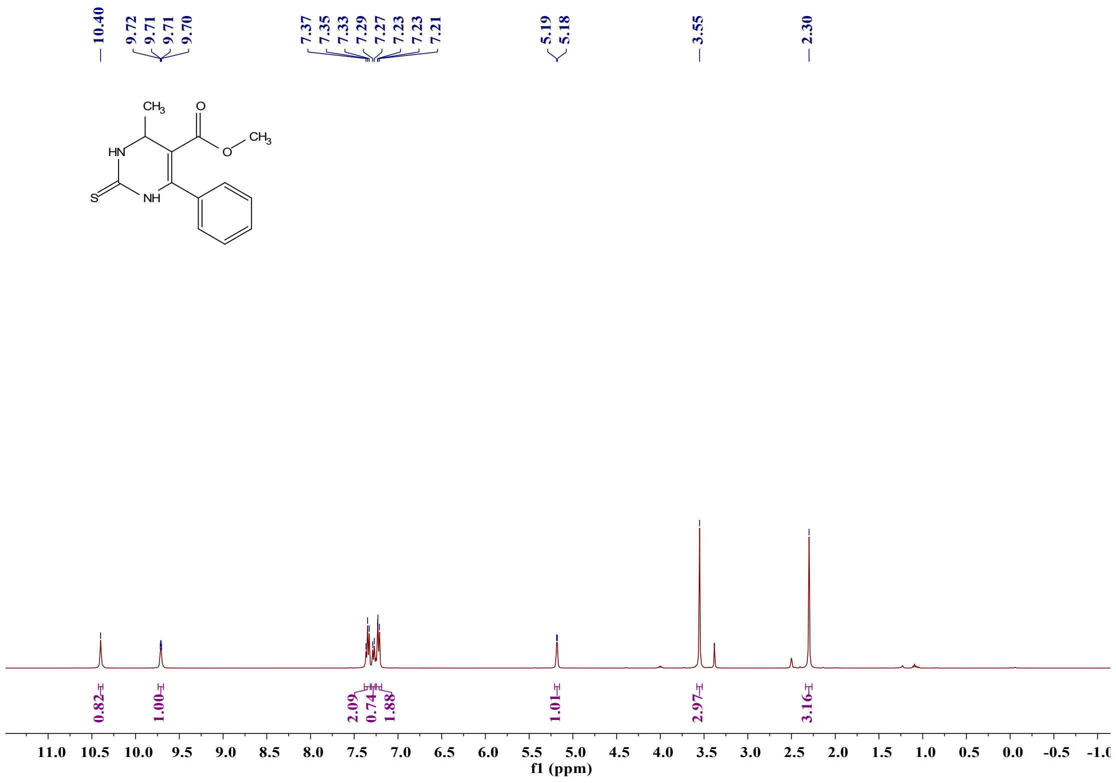
10.40
9.72 9.71 9.71 9.70
7.37 7.35 7.33 7.29 7.27 7.23 7.23 7.21
5.19 5.18
3.55
2.30
CH3
O
HN
O
CH3
S
NH
0.82
1.00
2.09 0.74 1.88
1.01
2.97
3.16
11.0 10.5 10.0 9.5 9.0 8.5 8.0 7.5 7.0 6.5 6.0 5.5 5.0 4.5 4.0 3.5 3.0 2.5 2.0 1.5 1.0 0.5 0.0 -0.5
f1 (ppm)

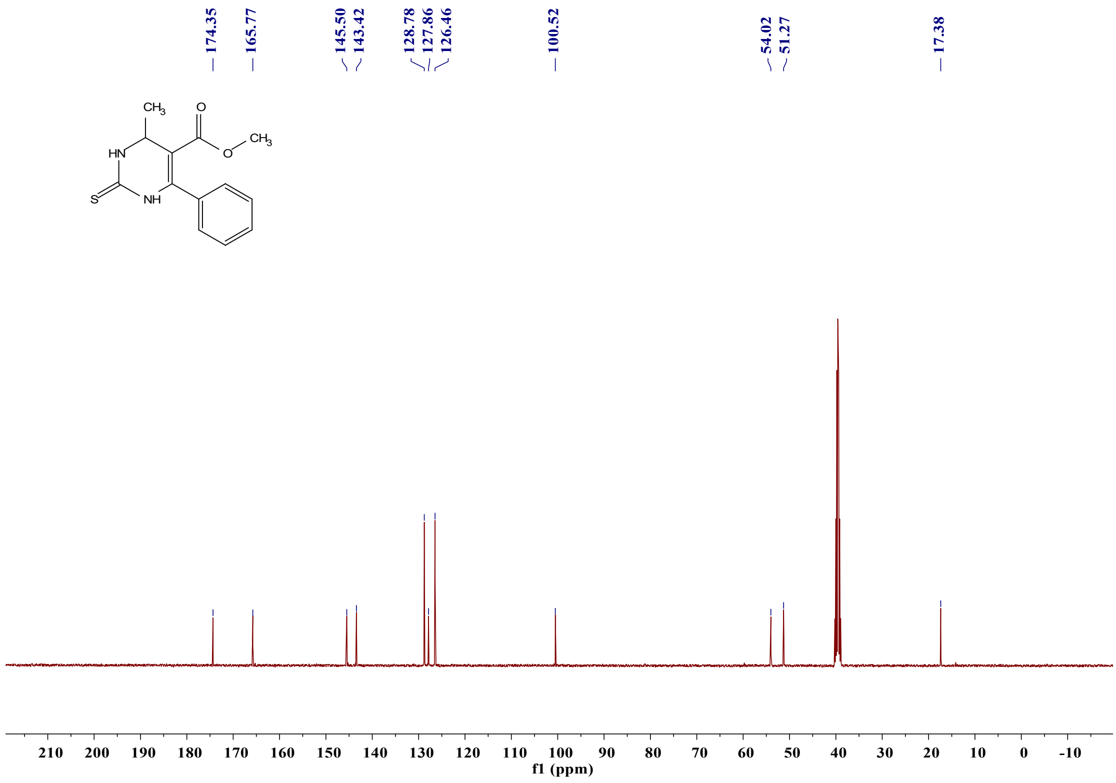
174.35
165.77
145.50 143.42
128.78 127.86 126.46
100.52
54.02 51.27
17.38
CH3
O
HN
O
CH3
S
NH
210 200 190 180 170 160 150 140 130 120 110 100 90 80 70 60 50 40 30 20 10 0 -10
f1 (ppm)

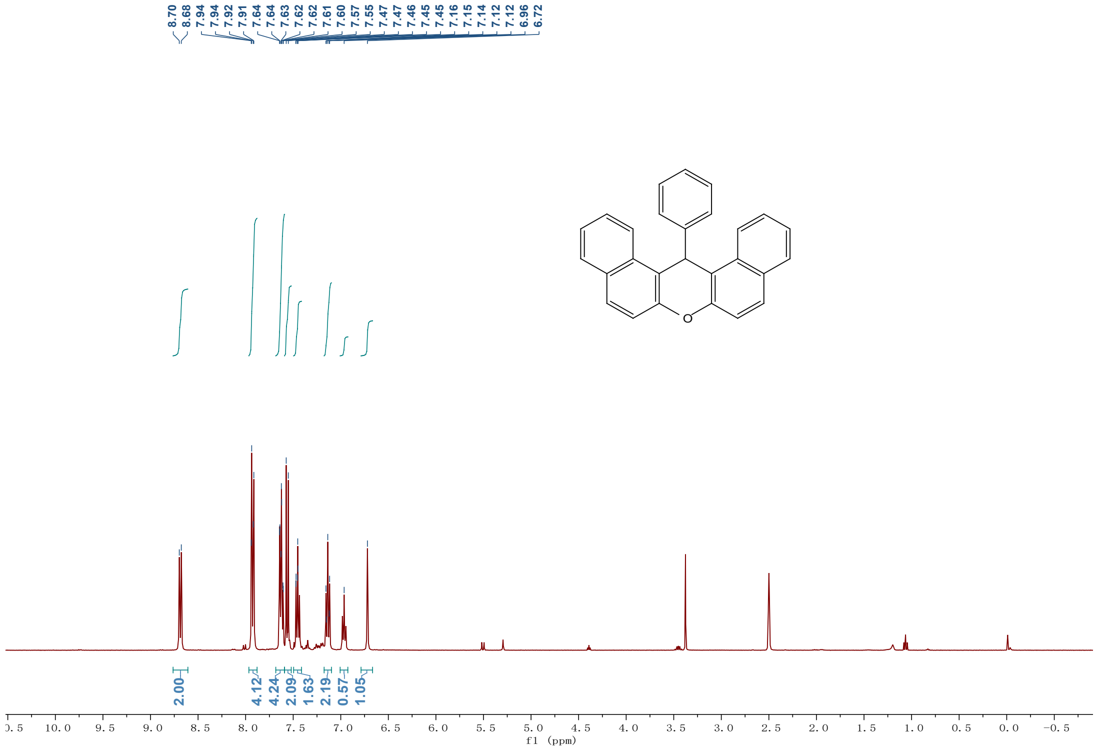

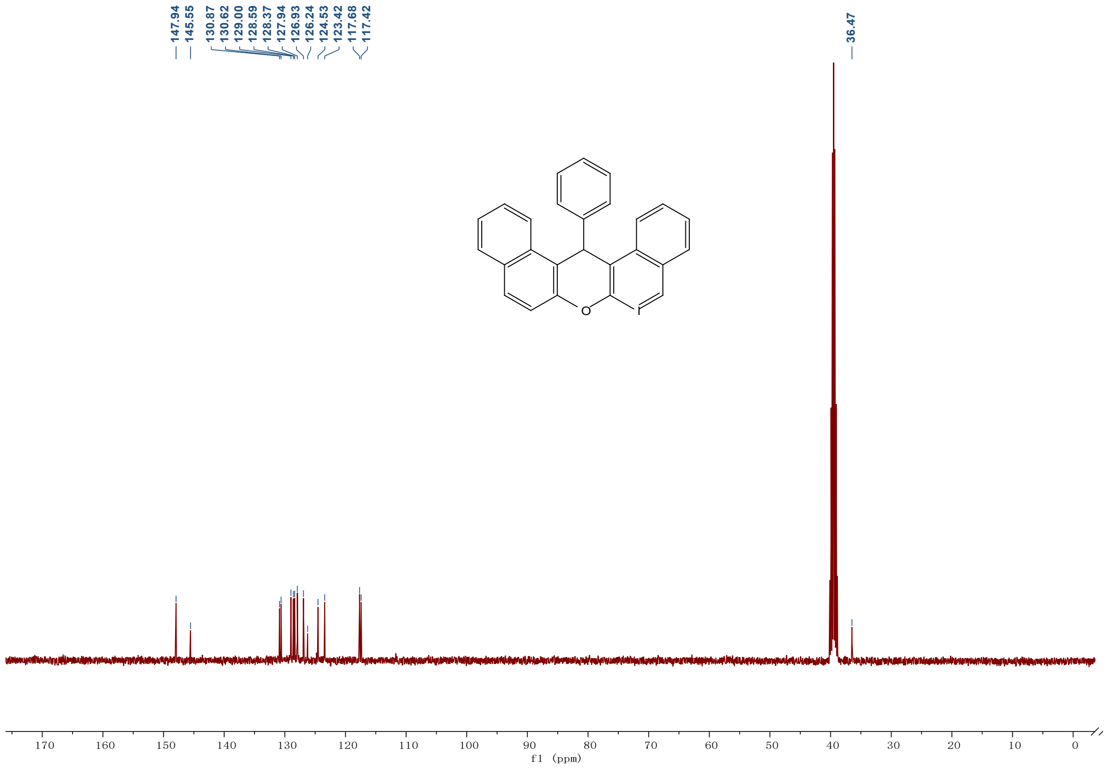

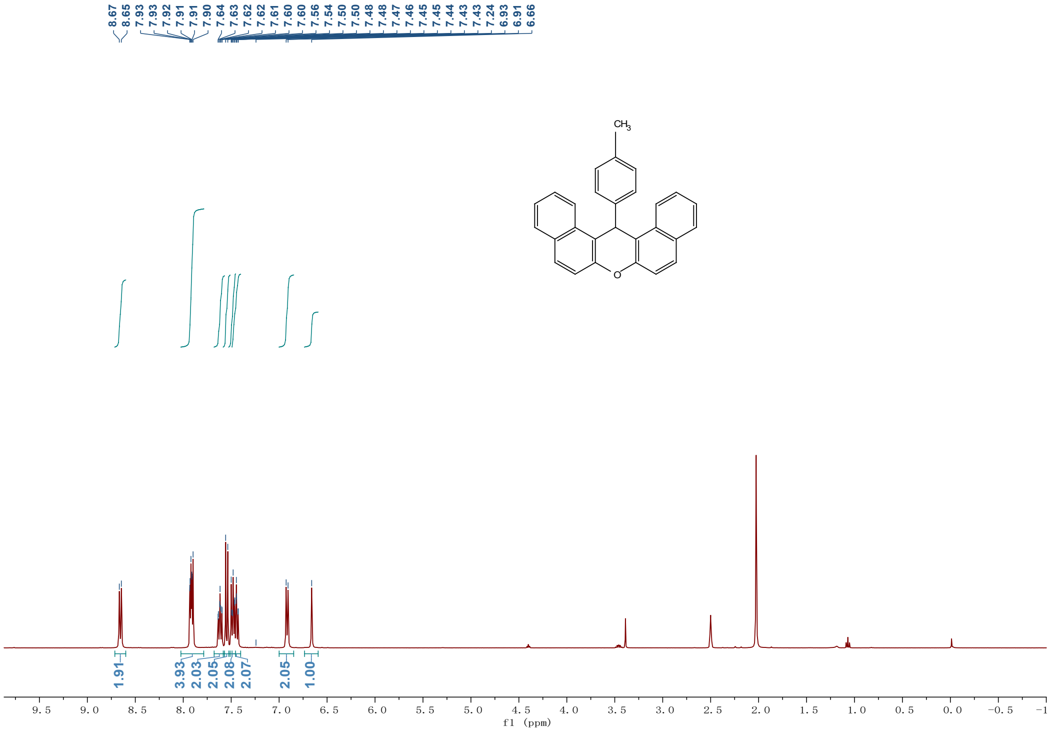
8.67
8.65
7.93
7.93
7.92
7.91
7.91
7.90
7.64
7.63
7.62
7.62
7.61
7.60
7.60
7.56
7.54
7.50
7.50
7.48
7.48
7.47
7.46
7.45
7.45
7.44
7.43
7.43
7.24
6.93
6.91
6.66
CH3
O
1.91
3.93
2.03
2.05
2.08
2.07
2.05
1.00
9.5 9.0 8.5 8.0 7.5 7.0 6.5 6.0 5.5 5.0 4.5 4.0 3.5 3.0 2.5 2.0 1.5 1.0 0.5 0.0 −0.5 −1
f1 (ppm)

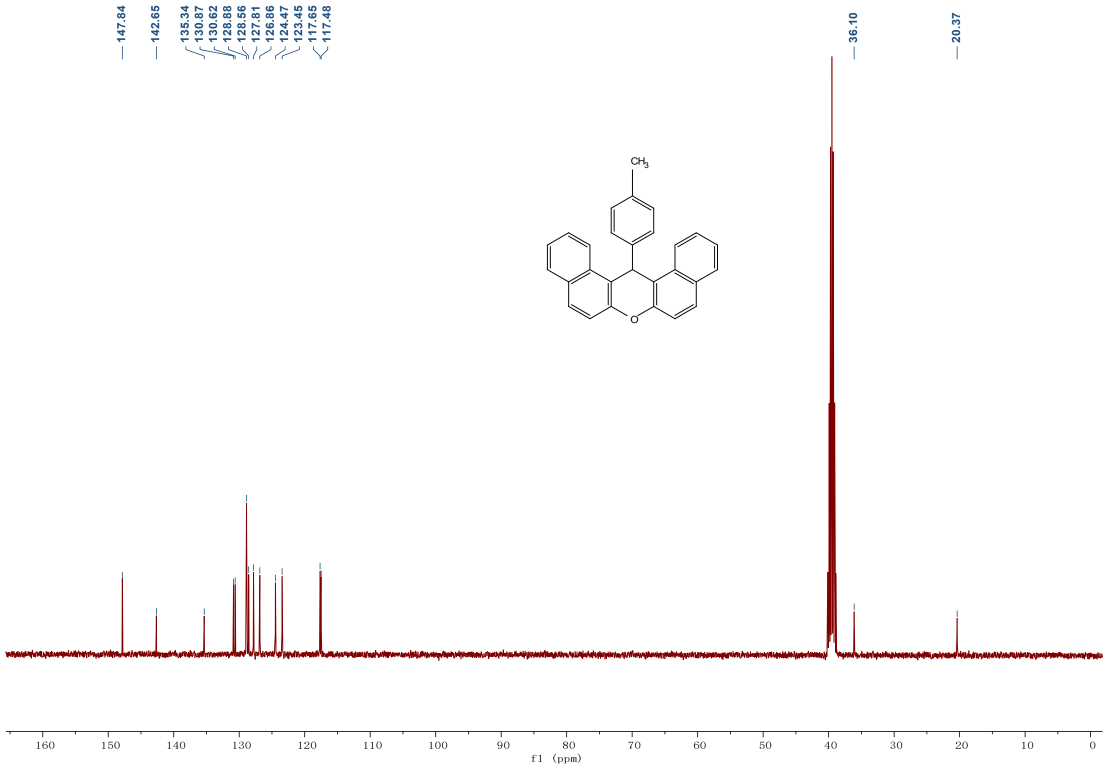
147.84
142.65
135.34
130.87
130.62
128.88
128.56
127.81
126.86
124.47
123.45
117.65
117.48
36.10
20.37
CH3
O
160 150 140 130 120 110 100 90 80 70 60 50 40 30 20 10 0
f1 (ppm)

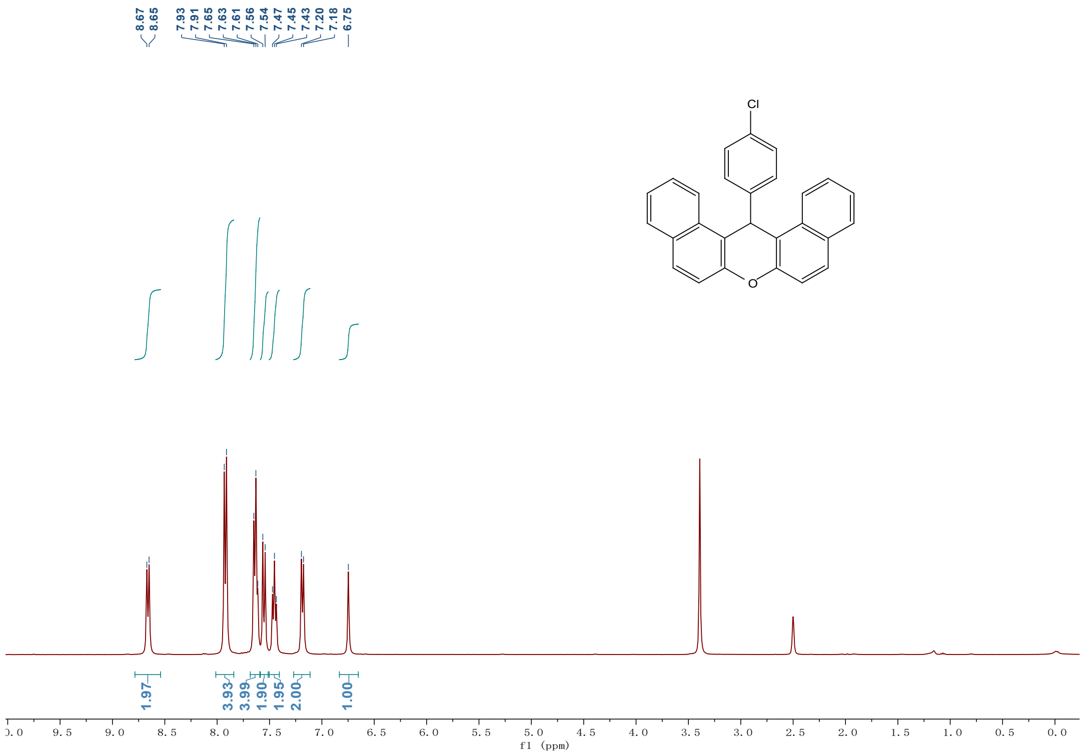

8.67
8.65
7.93
7.91
7.65
7.63
7.61
7.56
7.54
7.47
7.45
7.43
7.20
7.18
6.75
Cl
O
1.97
3.93
3.99
1.90
1.95
2.00
1.00
f1 (ppm)

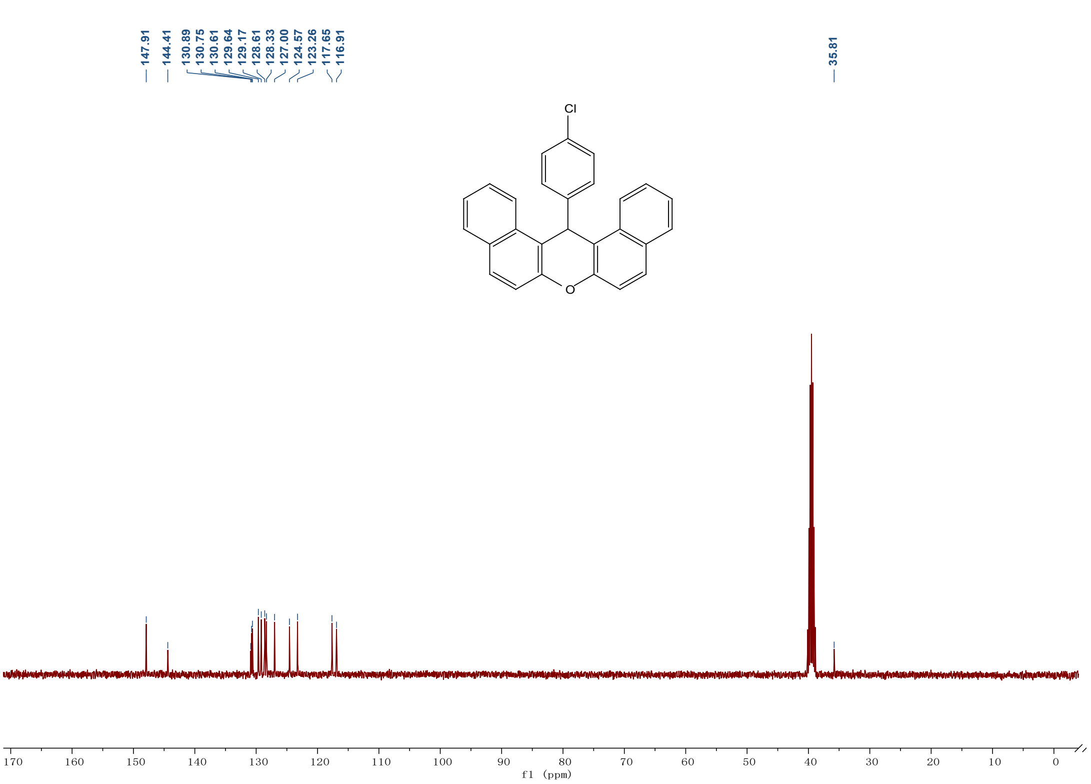

147.91
144.41
130.89
130.75
130.61
129.64
129.17
128.61
128.33
127.00
124.57
123.26
117.65
116.91
35.81
Cl
O
f1 (ppm)

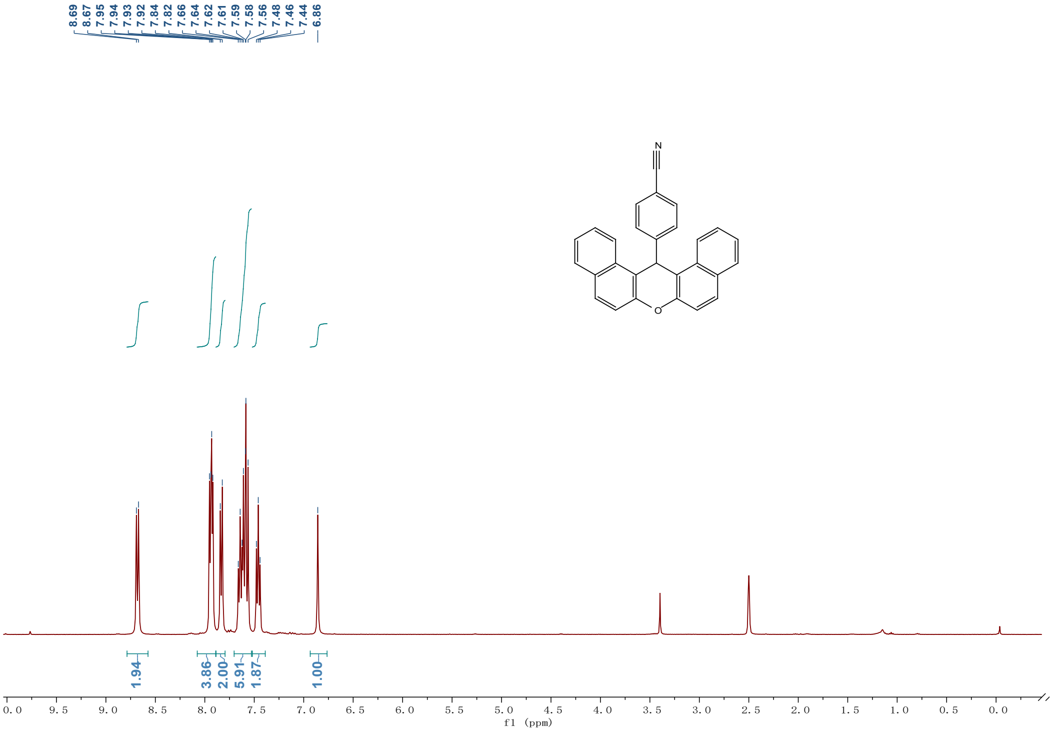
8.69
8.67
7.95
7.94
7.93
7.92
7.84
7.82
7.66
7.64
7.62
7.61
7.59
7.58
7.56
7.48
7.46
7.44
6.86
N
O
1.94
3.86
2.00
5.91
1.87
1.00
f1 (ppm)

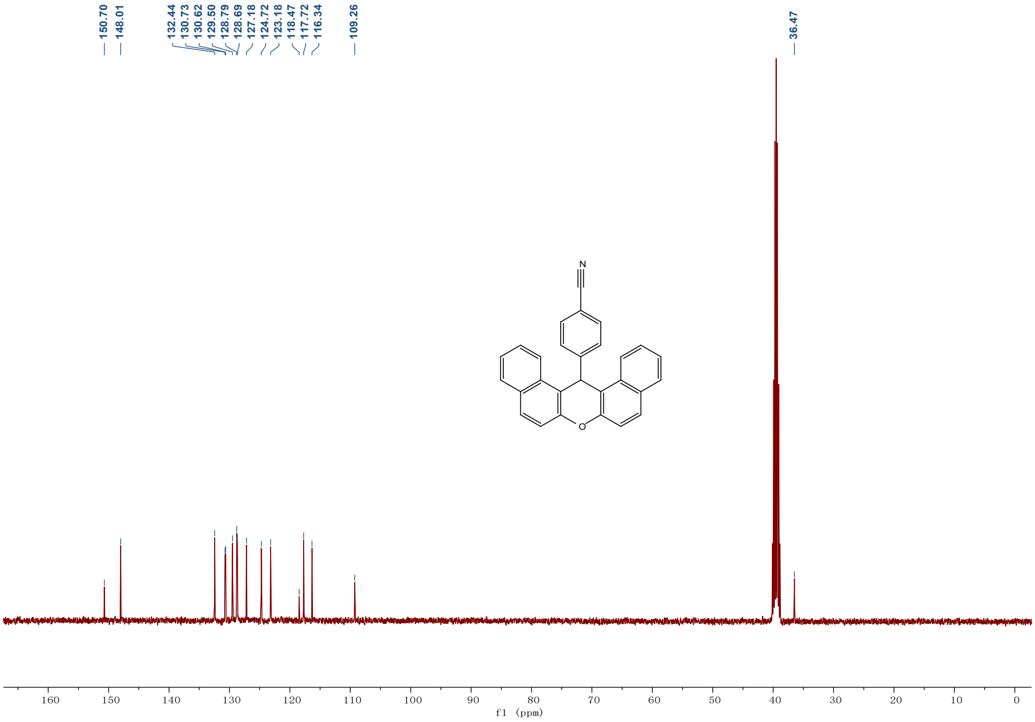
150.70
148.01
132.44
130.73
130.62
129.50
128.79
128.69
127.18
124.72
123.18
118.47
117.72
116.34
109.26
36.47
N
O
f1 (ppm)

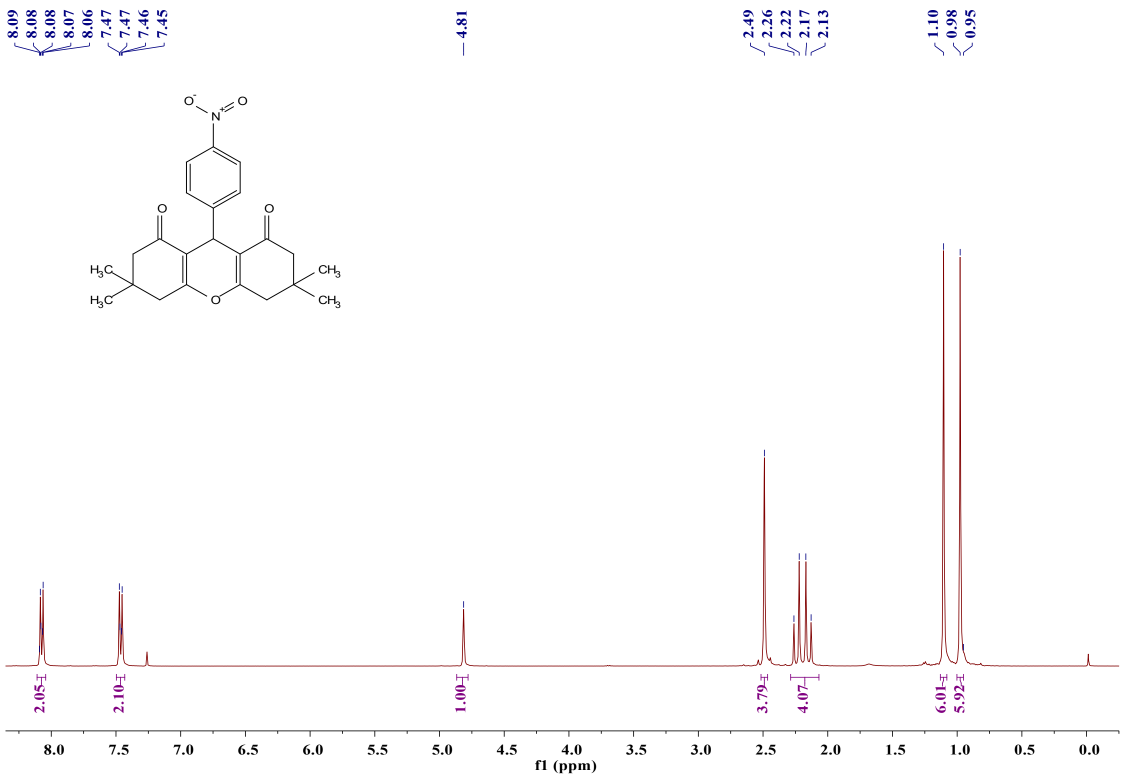
8.09
8.08
8.08
8.07
8.06
7.47
7.47
7.46
7.45
4.81
2.49
2.26
2.22
2.17
2.13
1.10
0.98
0.95
2.05
2.10
1.00
3.79
4.07
6.01
5.92
8.0 7.5 7.0 6.5 6.0 5.5 5.0 4.5 4.0 3.5 3.0 2.5 2.0 1.5 1.0 0.5 0.0
f1 (ppm)
O
N
O
O
O
H3C
H3C
CH3
CH3
O

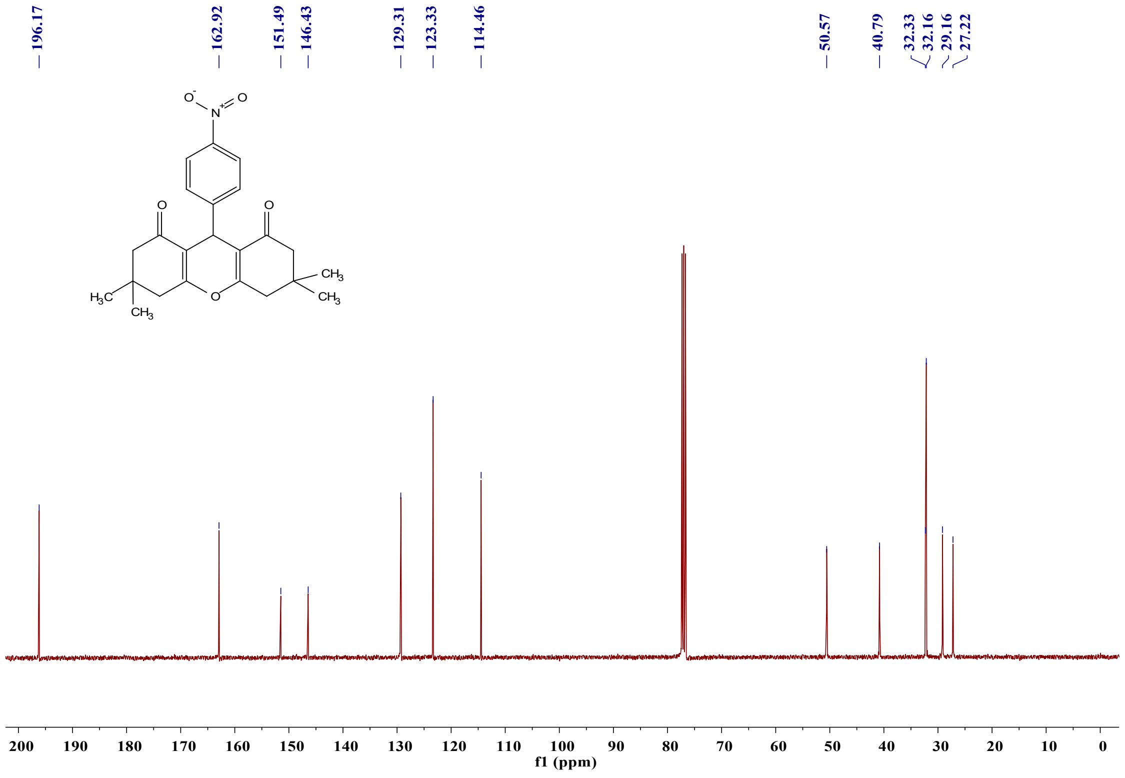
196.17
162.92
151.49
146.43
129.31
123.33
114.46
50.57
40.79
32.33
32.16
29.16
27.22
200 190 180 170 160 150 140 130 120 110 100 90 80 70 60 50 40 30 20 10 0
f1 (ppm)
O
N
O
O
O
H3C
CH3
CH3
CH3
O

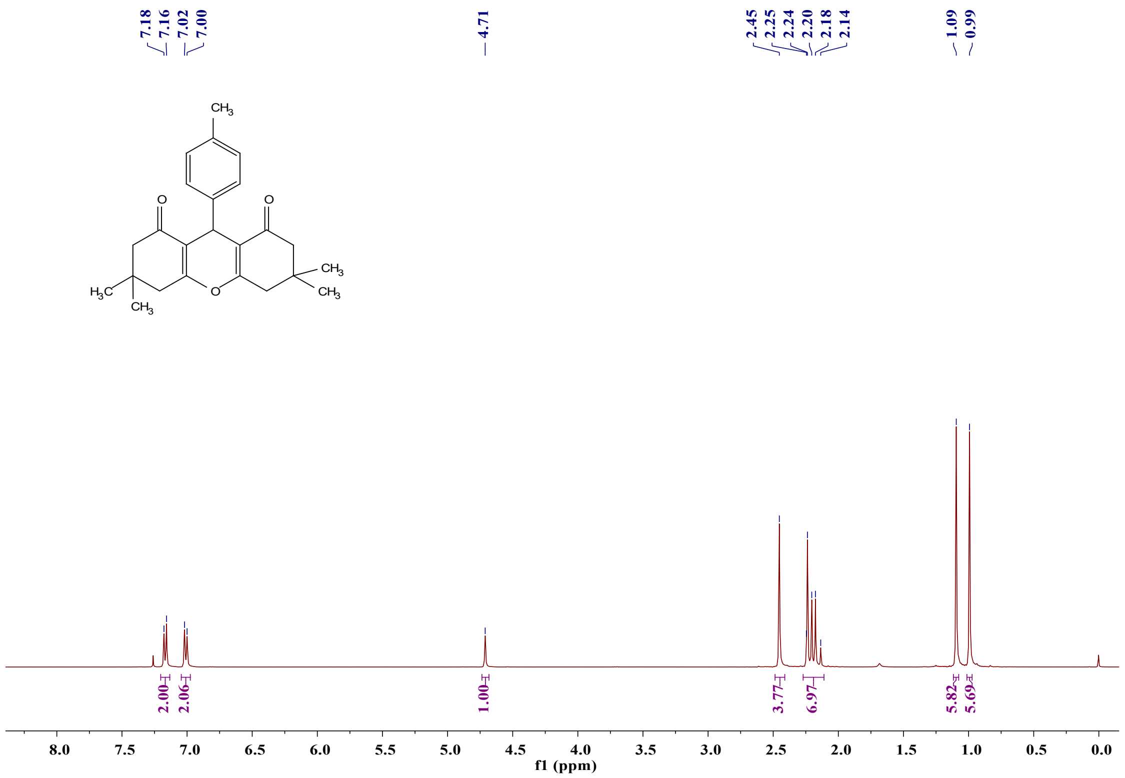
7.18
7.16
7.02
7.00
4.71
2.45
2.25
2.24
2.20
2.18
2.14
1.09
0.99
CH3
O
O
CH3
H3C
CH3
CH3
O
2.00
2.06
1.00
3.77
6.97
5.82
5.69
8.0 7.5 7.0 6.5 6.0 5.5 5.0 4.5 4.0 3.5 3.0 2.5 2.0 1.5 1.0 0.5 0.0
f1 (ppm)

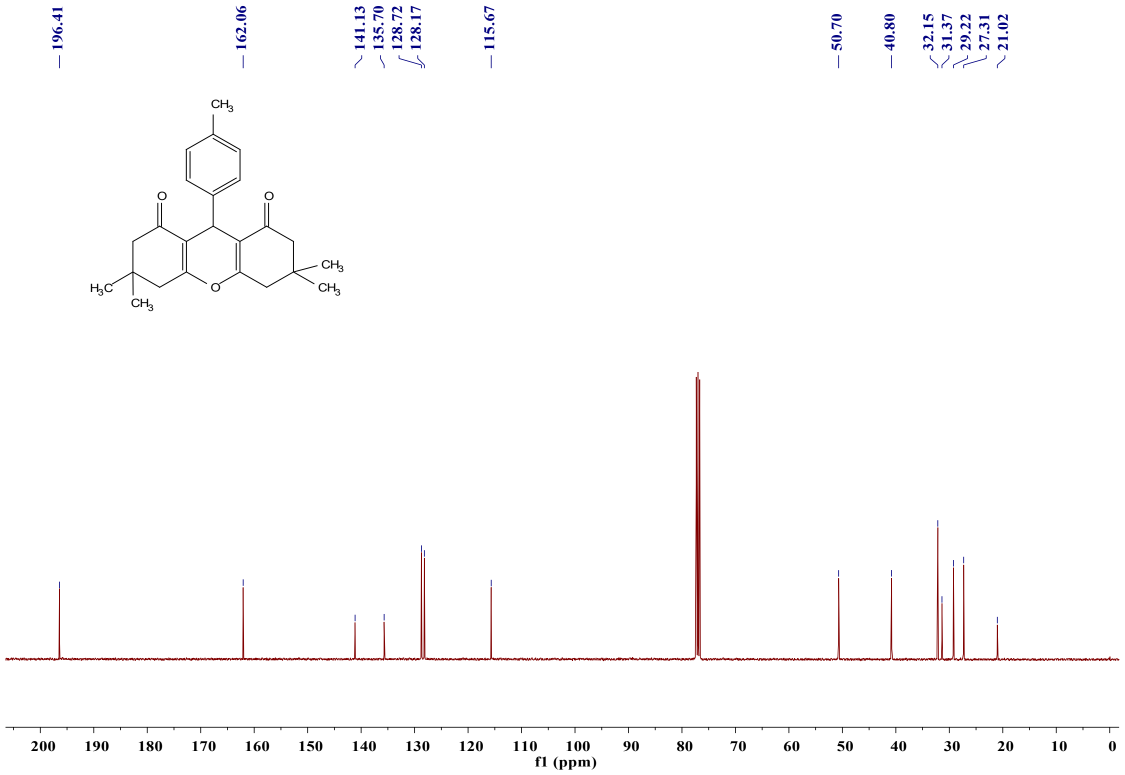
196.41
162.06
141.13
135.70
128.72
128.17
115.67
50.70
40.80
32.15
31.37
29.22
27.31
21.02
CH3
O
O
CH3
H3C
CH3
CH3
O
200 190 180 170 160 150 140 130 120 110 100 90 80 70 60 50 40 30 20 10 0
f1 (ppm)

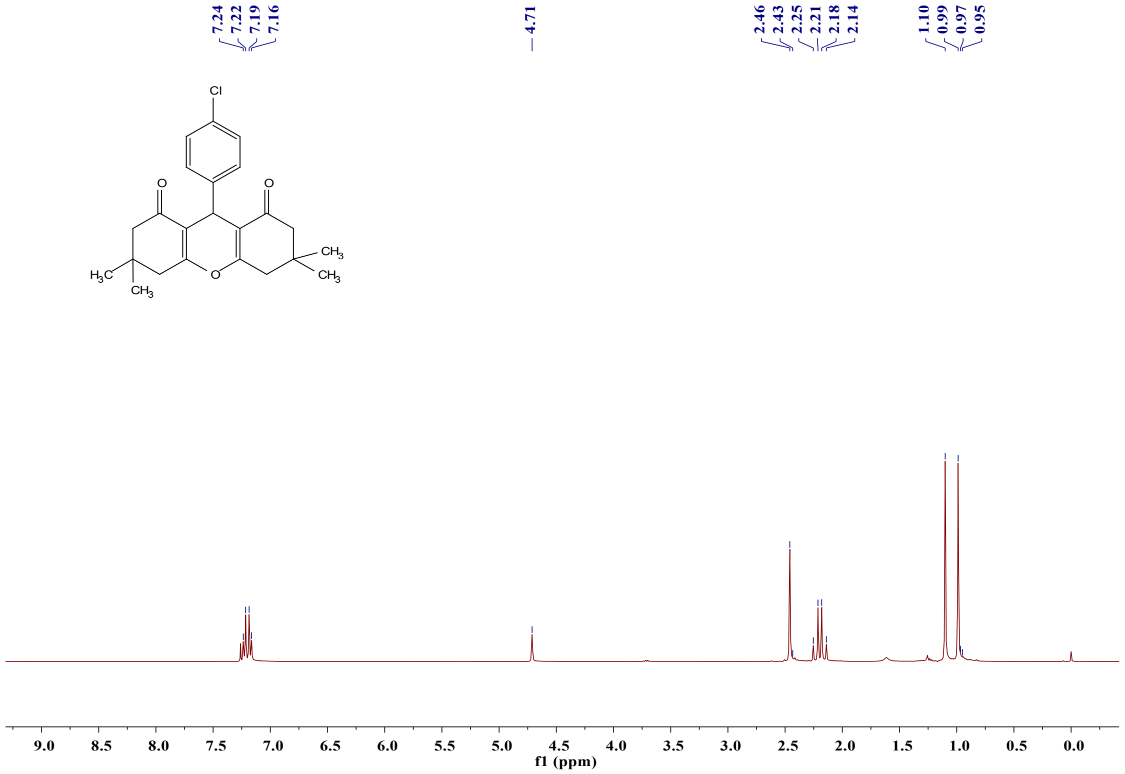
7.24
7.22
7.19
7.16
4.71
2.46
2.43
2.25
2.21
2.18
2.14
1.10
0.99
0.97
0.95
Cl
O
O
O
CH3
CH3
H3C
CH3
9.0 8.5 8.0 7.5 7.0 6.5 6.0 5.5 5.0 4.5 4.0 3.5 3.0 2.5 2.0 1.5 1.0 0.5 0.0
f1 (ppm)

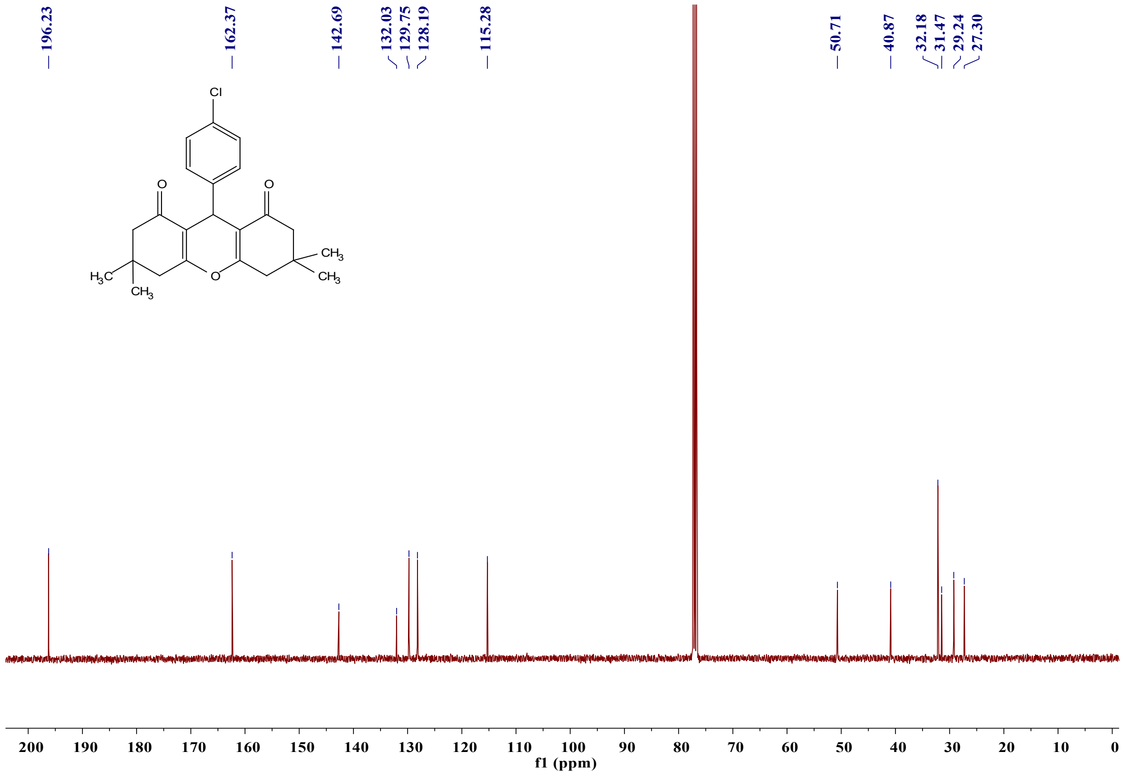
196.23
162.37
142.69
132.03
129.75
128.19
115.28
50.71
40.87
32.18
31.47
29.24
27.30
Cl
O
O
O
CH3
CH3
H3C
CH3
200 190 180 170 160 150 140 130 120 110 100 90 80 70 60 50 40 30 20 10 0
f1 (ppm)

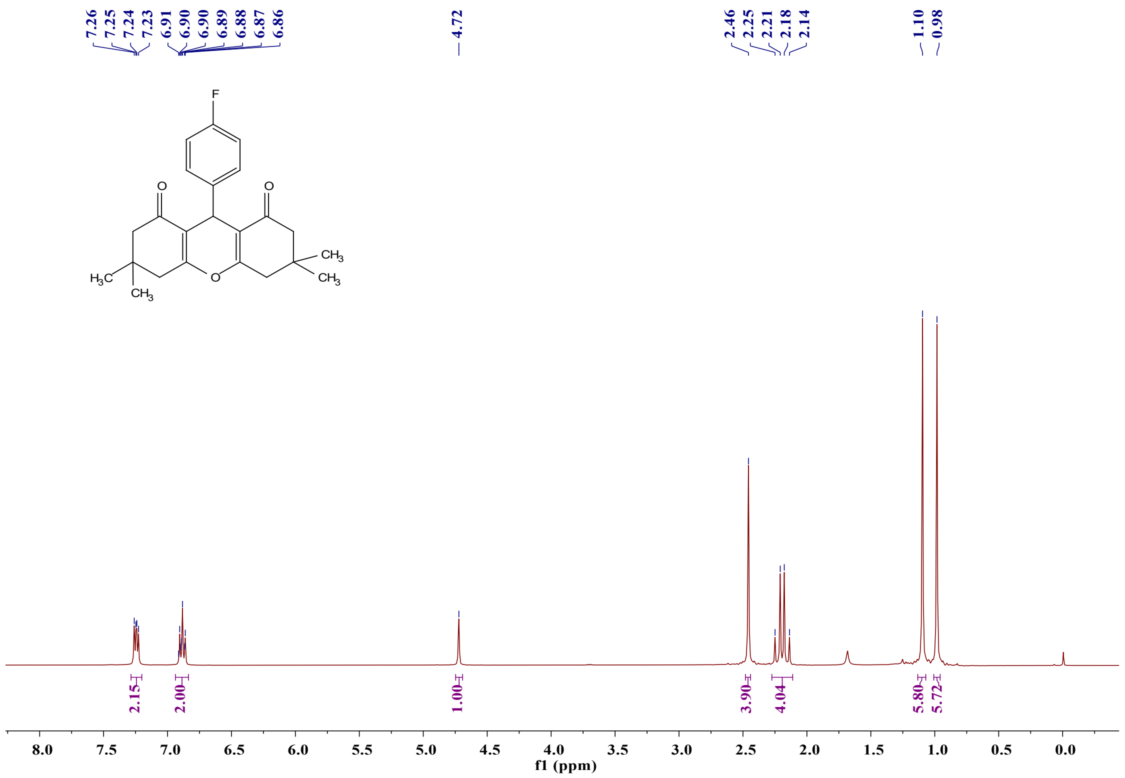
7.26
7.25
7.24
7.23
6.91
6.90
6.90
6.89
6.88
6.87
6.86
4.72
2.46
2.25
2.21
2.18
2.14
1.10
0.98
F
O
O
CH3
H3C
CH3
O
CH3
2.15
2.00
1.00
3.90
4.04
5.80
5.72
8.0
7.5
7.0
6.5
6.0
5.5
5.0
4.5
4.0
3.5
3.0
2.5
2.0
1.5
1.0
0.5
0.0
f1 (ppm)

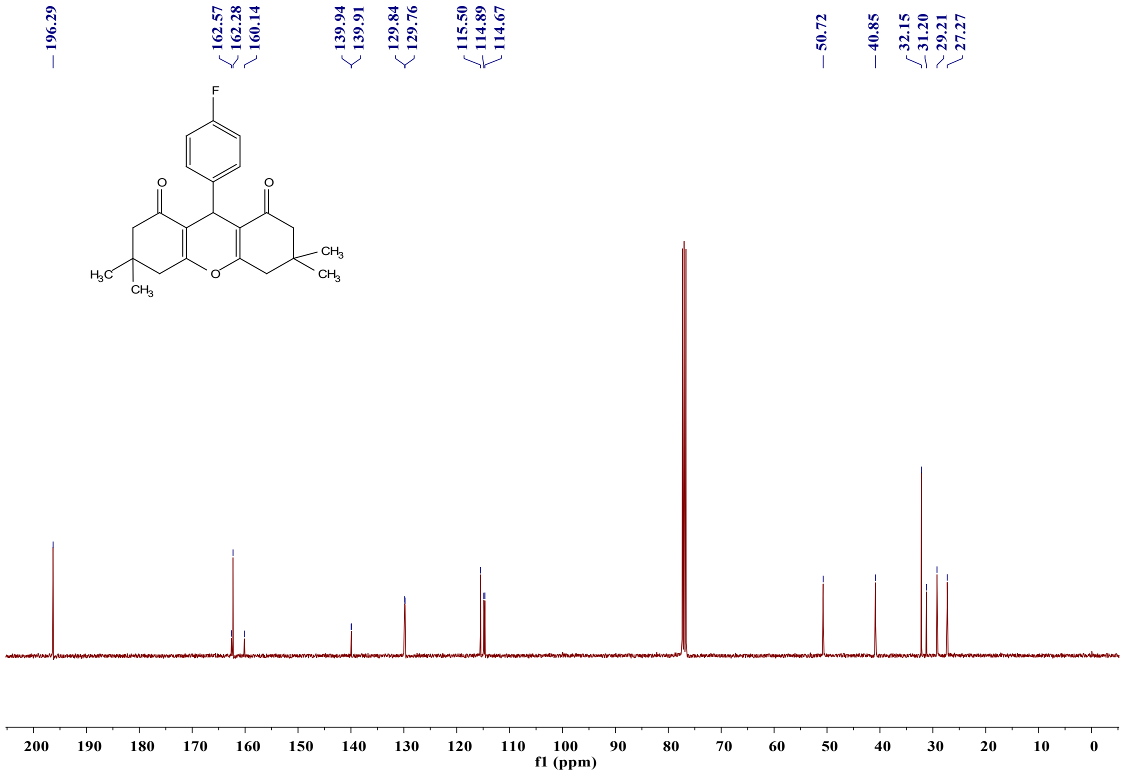
196.29
162.57
162.28
160.14
139.94
139.91
129.84
129.76
115.50
114.89
114.67
50.72
40.85
32.15
31.20
29.21
27.27
F
O
O
CH3
H3C
CH3
O
CH3
200
190
180
170
160
150
140
130
120
110
100
90
80
70
60
50
40
30
20
10
0
f1 (ppm)

References

1. H. S. Oboudatian, H. Naeimi and M. Moradian, *RSC Advances*, 2021, **11**, 7271-7279.
2. Z. Zhao, H. Dai and L. Shi, *Chemical Papers*, 2020, **75**, 583-590.
3. Z.-B. Xie, L.-H. Fu, J. Meng, J. Lan, Z.-Y. Hu and Z.-G. Le, *Bioorganic Chemistry*, 2020, **101**, 103949.
4. M. A. Douzandegi Fard, H. Ghafuri and A. Rashidizadeh, *Microporous and Mesoporous Materials*, 2019, **274**, 83-93.
5. L. G. do Nascimento, I. M. Dias, G. B. Meireles de Souza, I. Dancini-Pontes, N. R. C. Fernandes, P. S. de Souza, G. Roberto de Oliveira and C. G. Alonso, *The Journal of Organic Chemistry*, 2020, **85**, 11170-11180.
6. X. Yue, Z. Wu, G. Wang, Y. Liang, Y. Sun, M. Song, H. Zhan, S. Bi and W. Liu, *RSC Advances*, 2019, **9**, 28718-28723.
7. D. Fang, K. Gong and Z.-L. Liu, *Catalysis Letters*, 2008, **127**, 291-295.
